# Anti-inflammatory effect of euphane- and tirucallane-type triterpenes isolated from the traditional herb *Euphorbia neriifolia* L

**DOI:** 10.3389/fchem.2023.1223335

**Published:** 2023-06-23

**Authors:** Stephen S. Chang, Hung-Tse Huang, Wen-Chi Wei, I-Wen Lo, Yu-Chi Lin, Chih-Hua Chao, Geng-You Liao, Yuh-Chiang Shen, Jih-Jung Chen, Tsung-Lin Li, Liang-Tzung Lin, Chen-Jei Tai, Yao-Haur Kuo, Chia-Ching Liaw

**Affiliations:** ^1^ National Research Institute of Chinese Medicine, Ministry of Health and Welfare, Taipei, Taiwan; ^2^ Ph.D. Program in Clinical Drug Development of Herbal Medicine, College of Pharmacy, Taipei Medical University, Taipei, Taiwan; ^3^ Genomics Research Center, Academia Sinica, Taipei, Taiwan; ^4^ Chinese Medicine Research and Development Center, China Medical University Hospital, Taichung, Taiwan; ^5^ School of Pharmacy, China Medical University, Taichung, Taiwan; ^6^ Institute of Physiology, School of Medicine, National Yang Ming Chiao Tung University, Taipei, Taiwan; ^7^ Department of Pharmacy, National Yang Ming Chiao Tung University, Taipei, Taiwan; ^8^ Department of Microbiology and Immunology, School of Medicine, College of Medicine, Taipei Medical University, Taipei, Taiwan; ^9^ Graduate Institute of Medical Sciences, College of Medicine, Taipei Medical University, Taipei, Taiwan; ^10^ Graduate Institute of Integrated Medicine, College of Chinese Medicine, China Medical University, Taichung, Taiwan; ^11^ Department of Biochemical Science and Technology, National Chiayi University, Chiayi, Taiwan

**Keywords:** *Euphorbia neriifolia* L., Euphorbiaceae, neritriterpenols, euphane, tirucallane, anti-inflammatory activity

## Abstract

The Euphorbiaceae plant *Euphorbia neriifolia* L. is distributed widely in India, Thailand, Southeastern China, and Taiwan and used as a carminative and expectorant to treat several inflammation-related diseases, such as gonorrhoea, asthma, and cancer. In the course of our search for potential anti-inflammatory agents from the titled plant, 11 triterpenes from the stem of *E. neriifolia* were isolated and reported in our previous endeavor. Given its rich abundance in triterpenoids, the ethanolic extract in this follow-up exploration has led to the isolation of additional eight triterpenes, including six new euphanes—neritriterpenols H and J–N (**1** and **3**–**7**)—one new tirucallane, neritriterpenol I (**2**), and a known compound, 11-oxo-kansenonol (**8**). Their chemical structures were elucidated on the basis of spectroscopic data, including 1D- and 2D NMR, and HRESIMS spectra. The absolute stereochemistry of neritriterpenols was determined by single-crystal X-ray diffraction analysis, ICD spectra, and DP4+ NMR data calculations. Compounds **1**–**8** were also evaluated for their anti-inflammatory activity by using lipopolysaccharide (LPS)-stimulated IL-6 and TNF-α on RAW 264.7 macrophage cells. Intriguingly, the euphane-type triterpenes (**1** and **3**–**8**) showed an inhibitory effect on LPS-induced IL-6 but not on TNF-α, while tirucallane-type triterpene **2** showed strong inhibition on both IL-6 and TNF-α.

## 1 Introduction

*Euphorbia neriifolia* L., commonly known as a hedge or an ornamental horticultural cactus-like fleshy plant, belongs to the spurge family (Euphorbiaceae), one of the largest families of flowering plants (Webster, 1994). *E. neriifolia* shows morphological characteristics of being a glabrous erect branched succulent, xerophytic shrub or tree, up to 1.8–4.5 m tall, with nodular cylindrical or fuzzy five-angled branches (Mali and Panchal, 2017). Like other *Euphorbia* plants, the white latex (a milky-sap-like fluid yielded when plants are injured) of *E. neriifolia* is poisonous and irritating to the skin and eyes (Sultana et al., 2022). As a double-edged sword, *E. neriifolia* is also remarkable as a medicinal plant being native to India that possesses various ethnomedicinal uses depending on different parts or the entirety of this plant. For example, the milky latex, which is traditionally used as a purgative, is known to be beneficial for tumors, abdominal troubles, and leukoderma therapeutics (Mali and Panchal, 2017). Although considerable phytochemical analyses and modern pharmacological studies of *E. neriifolia* were reported, the extracts or various constituents of the toxic milky latex/sap, leaves, stems, or whole plants of *E. neriifolia* remain attractive because additional bioactivities, including anti-infective (Sultana et al., 2022) and anti-human coronavirus (Pramanik et al., 2022), antioxidant (Kumar et al., 2021), anti-inflammatory (Palit et al., 2016), anti-carcinogenic (Sharma et al., 2011; Pracheta, 2013), hepatocarcinogenesis (Sharma and Janmeda, 2013) and antiangiogenic (Qi et al., 2020) activities, were found from time to time.

**FIGURE 1:**
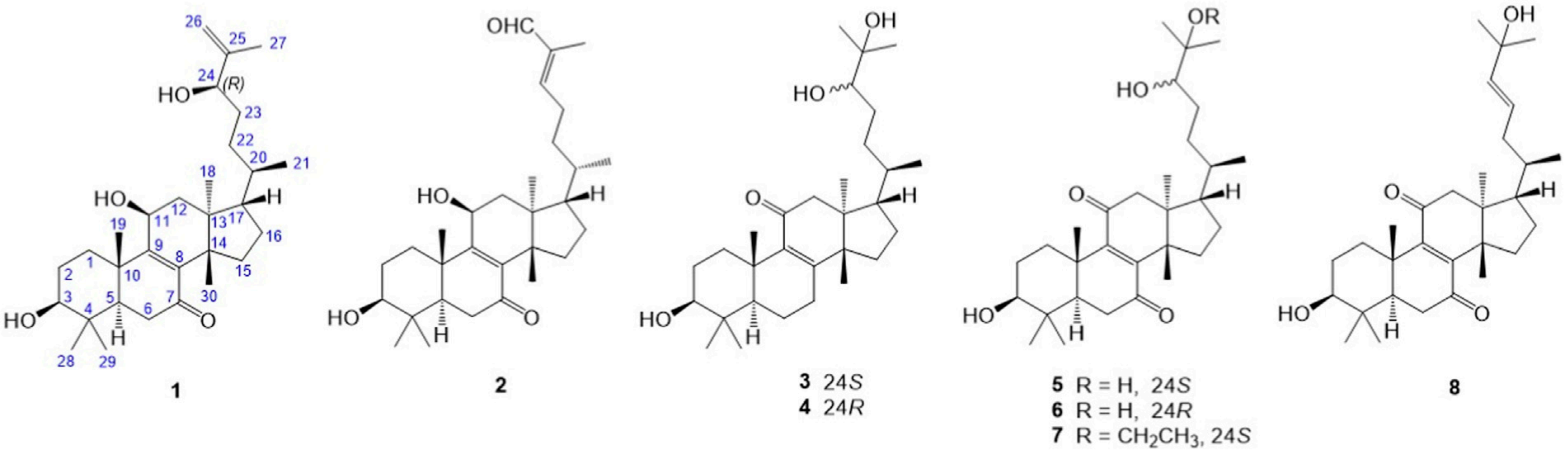
Chemical structures of compounds **1**–**8** from the *E. neriifolia* stem.

Over the last decade, several *Euphorbia* terpenes with favorable pharmacological effects were reported, in which the most well-known example is ingenol-3-angelate, a diterpenoid from *Euphorbia peplus* effective against precancerous actinic keratosis, which has received approval from FDA in 2012 and EMA in 2013 (Fidler and Goldberg, 2014). Most of the reported *Euphorbia* triterpenoids belong to tirucallane, cycloartane, lupane, oleanane, ursane, and taraxane subclasses (Kemboi et al., 2020). Recently, cycloartane triterpenes (euphonerins A‒G) and ingol diterpenes from *E. neriifolia* have been found to exhibit a death-receptor expression-enhancing activity (death receptors are expressed on many cell types, especially in the immune system), indicating that *Euphorbia* triterpenes have potential applications in immunomodulatory, anti-inflammatory, and analgesic treatments (Bigoniya and Rana, 2010; Toume et al., 2012; Mali and Panchal, 2017). In our previous study on bioactive constituents of *E. neriifolia*, 11 triterpenoids possessing anti-inflammatory and anti-cancer properties were isolated and identified as neritriterpenols A‒G, (23*E*)-eupha-8,23-diene-3β,25-diol-7-one, (+)-(24*S*)-eupha-8,25-diene-3β,24-diol-7-one, (24*R*)-eupha-8,25-diene-3β,24-diol-7-one, and sooneuphanone B (Chang et al., 2022). To elucidate the relationship between *E. neriifolia* triterpenoids and anti-inflammatory and anticancer activities, we continue to isolate and purify these components from the 95% ethanolic extract of *E. neriifolia* stems, and evaluate their anti-inflammatory activity by using LPS-stimulated RAW 264.7 macrophage cells. In the present article, we report the isolation and structure elucidation of eight triterpenes, including six new euphane-type triterpenes **1** and **3**–**7** (neritriterpenols H and J–N) and a new tirucallane-type triterpene **2** (neritriterpenol I), as well as a known compound, 11-oxo-kansenonol (**8**) (Figure 1). Compounds **1**‒**8** were purified by sequential column chromatography, and all the structures were elucidated from the 1D- and 2D-NMR, IR, and MS analyses; meanwhile, the configurations of their chiral hydroxyl groups were determined by optical dispersion (OD), induced circular dichroism (ICD), DP4+ NMR data calculation analysis, and single-crystal X-ray diffraction data, as well as a spectroscopic comparison with the reported literature data. In addition, we also evaluated each anti-inflammatory activity of these isolated compounds by measuring the effect of **1**‒**8** on the suppression of IL-6 and TNF-α in LPS-stimulated RAW 264.7 macrophages.

## 2 Materials and methods

### 2.1 General experimental procedures

Optical rotations were recorded using a JASCO P-2000 polarimeter. Infrared (IR) spectra were obtained using a Thermo Scientific Nicolet iS5 FTIR Spectrometer. The circular dichroism (CD) spectra were measured using a JASCO J-715 spectropolarimeter. Nuclear magnetic resonance (NMR) spectra were recorded using a Varian Unity Inova 500-MHz instrument with a 5-mm SWPFG/TRPFG probe. High-resolution electrospray ionization mass spectrometry (HRESIMS) data were measured using a Thermo Scientific Q Exactive Focus Orbitrap LC-MS/MS instrument with an UltiMate 3000 UHPLC System. Silica gel 60 (Merck, 70–230 and 230–400 mesh), C_18_ gel (Chromatorex, 40–75 mesh), and Sephadex LH-20 (GE) were used for open-column chromatography. Thin-layer chromatography (TLC) analyses were conducted on pre-coated silica gel plates (Merck, Kieselgel 60 F_254_, 1 mm), sprayed with anisaldehyde–sulfuric acid reagent, and then heated at 100°C. Preparative HPLC was performed using a Shimadzu LC-8A pump and an SPD-20A UV detector, equipped with a COSMOSIL 5C_18_ AR-II column (i.d. 250 × 20 mm, Nacalai Tesque Inc.). A Bruker D8 VENTURE single-crystal XRD diffractometer equipped with Mo–Kα radiation sources was used to record single-crystal X-ray diffraction.

### 2.2 Plant material

The fleshy stems of *E. neriifolia* L. were purchased from the traditional herbal market, Tianshun Ginseng Medicine and Herbal Shop, Taipei, Taiwan. The material was identified by one of the authors Dr. Chia-Ching Liaw, the curator of the Herbarium of National Institute Research of Chinese Medicine, Taiwan. A voucher specimen (EN-2018–10-001) was deposited at the Herbarium of NRICM, Taipei, Taiwan.

### 2.3 Extraction, isolation, and purification

The fleshy stems of *E. neriifolia* L. (45 kg) were air-dried and chopped. The dry material (12.0 kg) was extracted with 95% EtOH (80 L) at 50°С/24 h three times, and the extracts were combined and concentrated under reduced pressure to obtain the crude extract. The EtOH extract (*ca.*1.9 kg) suspended in H_2_O was sequentially partitioned with hexanes and CH_2_Cl_2_ to yield three partition layers. The CH_2_Cl_2_ layer (END, 266.1 g, 14.0%) was chromatographed by flash column chromatography on silica gel and eluted with CH_2_Cl_2_/MeOH (97:3–3:1, v/v) to yield three fractions (ENDD1∼3). The fraction ENDD2 (97.3 g, 5.12%) was fractionated using the HPLC system and eluted with MeOH in H_2_O (60–100%) to obtain four subfractions (ENDD2.1∼2.4). The subfraction ENDD2.2 (23.2 g, 1.22%) was chromatographed using the Sephadex LH-20 column and eluted with 100% methanol to obtain seven parts (ENDD2.2.1∼7). ENDD2.2.4 was subjected to RP-HPLC using a C_18_ column detected at 210 nm, and then, the subfractions were obtained (ENDD.2.2.4.1∼6). Compounds **5** (5.0 mg, 0.00026%, and R_t_: 67.9 min) and **6** (16.9 mg, 0.00088%, and R_t_: 65.4 min) were purified from the subfraction ENDD2.2.4.2 by HPLC and eluted with 40% acetonitrile (flow rate: 10.0 mL/min). The subfraction ENDD2.2.4.3 was purified by HPLC and eluted with 45% acetonitrile to obtain compounds **1** (4.1 mg, 0.00022%, and R_t_: 56.7 min) and **2** (5.5 mg, 0.00029%, and R_t_: 88.4 min). The subfraction ENDD2.2.4.4 was also purified by HPLC and eluted with 50% acetonitrile to obtain **8** (14.2 mg, 0.00075%, and R_t_: 50.4 min). Subfractions ENDD2.2.4.5 and ENDD2.2.4.6 were further purified by HPLC and eluted with 50% acetonitrile to yield **3** (5.1 mg, 0.00027%, and R_t_: 62.4 min), **4** (11.4 mg, 0.0006%, and R_t_: 60.3 min), and **7** (5.0 mg, 0.00026%, and R_t_: 108.2 min).

### 2.4 Spectroscopic data

#### 2.4.1 Neritriterpenol H (**1**)

White amorphous powder; [*α*] +28.9 (*c* 0.1, MeOH); ν_max_ (KBr) 3429, 2927, 1672, 1465, 1376, 1270, 1183, and 1037 cm^−1^; CD (*c* 0.02, MeOH) 216 (+10.67), 265 (−11.65), and 349 (+5.26) nm; ^1^H- (500 MHz) and ^13^C- (125 MHz) NMR spectroscopic data (CDCl_3_) in Tables 1, 2, respectively; HRESIMS at *m/z* 495.3451 [M + Na]^+^ (calcd. for C_30_H_48_O_4_Na, 495.3445).

**TABLE 1:** ^1^H-NMR spectroscopic data on compounds **1**–**8** **(CDCl_3_ and 500 MHz)**.

No	1	2	3	4	5	6	7	8
1	1.58 m	1.59 m	0.99 m	0.97 dd (5.0, 13.5)	1.07 m	1.12 m	1.13 m	1.12 m
	2.47 m	2.45 m	2.61 dt (3.5, 13.5)	2.61 td (3.5, 13.5)	2.49 m	2.54 m	2.55 m	2.55 m
2	1.78 m (2H)	1.71 m	1.41 m	1.41 m	1.72 m	1.73 m	1.75 m	1.73 m
		1.79 m	1.68 m	1.68 m	1.75 m	1.75 m		1.75 m
3	3.34 m	3.34 m	3.27 dd (5.5, 11.0)	3.26 dd (5.5, 11.0)	3.28 dd (4.5, 12.0)	3.31 dd (4.0, 11.5)	3.32 dd (6.0, 10.5)	3.32 dd (4.0, 11.5)
5	1.68 dd (6.0, 12.5)	1.68 dd (6.0, 12.0)	1.03 dd (2.0, 12.5)	1.03 dd (1.5, 12.5)	1.62 m	1.66 dd (4.5, 13.5)	1.66 m	1.66 m
6	2.44 m	2.47 m (2H)	1.47 m	1.46 m	2.44 dd (13.5, 18.5)	2.49 dd (13.5, 18.5)	2.47 dd (13.5, 18.0)	2.47 dd (13.0, 18.0)
	2.50 m		1.79 m	1.79 m	2.50 m	2.55 dd (4.5, 18.5)	2.55 m	2.55 m
7			2.19 m	2.18 ddd (7.0, 11.5, 19.5)				
			2.36 dd (5.5, 20.0)	2.35 dd (7.5, 19.5)				
11	4.72 t (8.5)	4.73 t (8.0)						
12	1.83 m	1.83 m	2.46 d (18.5)	2.45 d (18.5)	2.48 d (20.0)	2.56 d (19.5)	2.59 d (19.0)	2.48 dd (19.0)
	2.43 m	2.38 m	2.57 d (18.5)	2.58 d (18.5)	2.63 d (20.0)	2.70 d (19.5)	2.70 d (19.0)	2.69 d (19.0)
15	1.46 m	1.46 m	1.39 m	1.39 m	1.62 m	1.67 m	1.66 m	1.67 m
	2.12 ddd (2.5, 10.0, 12.5)	2.14 m	1.75 m	1.74 m	2.12 m	2.16 m	2.16 ddd (2.5, 9.5, 13.0)	2.17 ddd (2.5, 9.5, 12.5)
16	1.35 m	1.38 m	1.42 m	1.42 m	1.37 m	1.41 m (2H)	1.41 m	1.42 m
	1.96 m	1.99 m	2.02 m	2.02 m	2.00 m		2.04 m	2.04 m
17	1.62 m	1.64 m	1.72 m	1.72 m	1.64 m	1.69 m	1.69 m	1.70 m
18	0.74 s	0.75 s	0.93 s	0.93 s	0.92 s	0.96 s	0.97 s	0.96 s
19	1.28 s	1.29 s	1.22 s	1.22 s	1.28 s	1.32 s	1.33 s	1.33 s
20	1.51 m	1.53 m	1.50 m	1.51 m	1.43 m	1.52 m	1.53 m	1.54 m
21	0.90 d (6.5)	0.95 d (6.5)	0.90 d (6.5)	0.89 d (6.5)	0.87 d (6.0)	0.90 d (7.5)	0.90 d (6.0)	0.89 d (6.5)
22	1.24 m	1.34 m	1.01 m	1.27 m	0.95 m	1.28 m	1.26 m	1.74 m
	1.60 m	1.80 m	1.86 m	1.59 m	1.81 m	1.60 m	1.65 m	2.25 ddd (3.5, 5.0, 13.0)
23	1.49 m	2.31 m	1.16 m	1.40 m	1.10 m	2.06 m (2H)	1.42 m	5.59 ddd (5.5, 7.0, 15.5)
	1.61 m	2.43 m	1.60 m	1.68 m	1.57 m			
24	4.06 t (7.0)	6.51 t (7.5)	3.30 dd (2.0, 10.5)	3.34 dd (6.0, 8.0)	3.25 dd (2.0, 10.0)	3.35 dd (4.0, 9.0)	3.42 dd (2.5, 9.0)	5.62 d (15.5)
26	4.88 brs	9.43 s	1.18 s	1.18 s	1.14 s	1.18 s	1.11 s	1.33 s
	4.97 brs							
27	1.79 s	1.78 s	1.24 s	1.23 s	1.20 s	1.23 s	1.15 s	1.33 s
28	0.94 s	0.94 s	0.85 s	0.85 s	0.88 s	0.92 s	0.92 s	0.92 s
29	1.02 s	1.02 s	1.06 s	1.05 s	1.06 s	1.04 s	1.05 s	1.04 s
30	1.17 s	1.18 s	1.04 brs	1.03 s	1.10 s	1.10 s	1.10 s	1.10 s
1′							3.45 q (7.0)	
2′							1.18 t (7.0)	

**TABLE 2:** ^13^C-NMR spectroscopic data on compounds **1**−**8** **(CDCl_3_ and 125 MHz)**.

No	1	2	3	4	5	6	7	8
1	33.7	33.7	34.1	34.1	33.9	33.9	33.9	33.9
2	27.4	27.4	27.9	27.9	27.4	27.3	27.4	27.4
3	78.3	78.2	78.9	78.9	77.9	77.9	78.0	77.9
4	39.1	39.1	39.0	39.0	38.6	38.6	38.6	38.6
5	49.3	49.3	51.7	51.7	48.5	48.5	48.5	48.5
6	35.9	35.8	18.1	18.1	35.8	35.8	35.8	35.8
7	200.1	200.0	29.7	29.7	199.9	200.0	200.0	199.9
8	140.4	140.4	161.5	161.6	149.8	149.8	149.7	149.7
9	161.2	161.0	139.6	139.6	154.8	154.8	154.9	154.8
10	39.6	39.6	37.1	37.1	38.0	38.0	38.0	38.1
11	68.1	68.1	199.1	199.2	202.0	202.2	202.1	201.8
12	42.8	43.0	51.1	51.1	51.5	51.5	51.2	51.4
13	46.2	48.1	44.7	44.6	45.1	45.1	45.2	45.2
14	48.0	46.2	51.2	51.2	47.9	47.9	47.9	47.9
15	31.8	31.8	30.0	30.0	31.8	31.8	31.8	31.8
16	27.8	27.8	27.5	27.5	28.1	28.0	28.3	27.9
17	48.5	48.7	50.3	50.5	49.4	49.4	49.6	49.0
18	16.3	16.4	17.7	17.7	18.6	18.5	18.4	18.7
19	19.7	19.7	19.8	19.8	17.8	17.7	17.7	17.7
20	35.6	35.7	36.5	35.6	36.5	35.4	35.2	36.1
21	18.9	18.7	18.7	18.4	18.7	18.4	18.3	18.7
22	31.0	34.0	32.2	31.8	32.3	31.6	31.6	37.8
23	31.3	26.0	28.6	28.2	28.8	28.2	27.6	124.8
24	76.2	154.9	79.3	78.4	79.3	78.2	76.3	139.8
25	147.8	139.3	73.2	73.1	73.2	73.2	77.4	70.7
26	111.0	195.3	23.4	23.4	23.3	23.3	19.2	29.9
27	17.6	9.3	26.5	26.6	26.6	26.6	21.5	29.9
28	15.2	15.2	15.7	15.7	15.1	15.1	15.1	15.1
29	27.6	27.6	28.3	28.3	27.6	27.6	27.6	27.6
30	25.7	25.7	24.2	24.2	24.0	24.0	24.0	24.0
1′							56.4	
2′							16.2	

#### 2.4.2 Neritriterpenol I (**2**)

White amorphous powder; [*α*] −8.3 (*c* 0.3, MeOH); ν_max_ (KBr) 3442, 2967, 2932, 2871, 1716, 1660, 1458, 1376, 1272, 1183, 1121, 1077, and 1040 cm^−1^; CD (*c* 0.02, MeOH) 216 (+11.40) and 249 (+3.62) nm; ^1^H- (500 MHz) and ^13^C- (125 MHz) NMR spectroscopic data (CDCl_3_) in Tables 1, 2, respectively; HRESIMS at *m/z* 493.3287 [M + Na]^+^ (calcd. for C_30_H_46_O_4_Na, 493.3288).

#### 2.4.3 Neritriterpenol J (**3**)

Colorless crystal; [*α*] +22.7 (*c* 0.3, MeOH); ν_max_ (KBr) 3417, 2967, 2925, 1640, 1462, 1378, 1292, 1240, 1173, and 1072 cm^−1^; CD (*c* 0.02, MeOH) 215 (+11.94), 261 (+4.06), and 313 (−0.55) nm; ^1^H- (500 MHz) and ^13^C- (125 MHz) NMR spectroscopic data (CDCl_3_) in Tables 1, 2, respectively; HRESIMS at *m/z* 497.3618 [M + Na]^+^ (calcd. for C_30_H_50_O_4_Na, 497.3601).

#### 2.4.4 Neritriterpenol K (**4**)

White amorphous powder; [*α*] +12.9 (*c* 0.3, MeOH); ν_max_ (KBr) 3419, 2970, 2871, 1643, 1596, 1460, 1381, 1267, 1173, 1075, and 1025 cm^−1^; CD (*c* 0.02, MeOH) 215 (+11.73), 257 (+8.26), and 313 (−2.17) nm; ^1^H- (500 MHz) and ^13^C- (125 MHz) NMR spectroscopic data (CDCl_3_) in Tables 1, 2, respectively; HRESIMS *m/z* 473.3638 [M − H]^−^ (calcd. for C_30_H_49_O_4_, 473.3265).

#### 2.4.5 Neritriterpenol L (**5**)

Colorless crystal; [*α*] +5.8 (*c* 0.2, MeOH); ν_max_ (KBr) 3432, 2923, 2854, 1672, 1467, 1378, 1270, 1235, 1183, and 1037 cm^−1^; CD (*c* 0.02, MeOH) 216 (+11.49) and 270 (+19.50) nm; ^1^H- (500 MHz) and ^13^C- (125 MHz) NMR spectroscopic data (CDCl_3_) in Tables 1, 2, respectively; HRESIMS at *m/z* 511.3397 [M + Na]^+^ (calcd. for C_30_H_48_O_5_Na, 511.3394).

#### 2.4.6 Neritriterpenol M (**6**)

White amorphous powder; [*α*] +9.2 (*c* 0.3, MeOH); ν_max_ (KBr) 3439, 2975, 2873, 1670, 1462, 1416, 1381, 1267, 1233, and 1183 cm^−1^; CD (*c* 0.02, MeOH) 215 (+12.08) and 273 (+10.23) nm; ^1^H- (500 MHz) and ^13^C- (125 MHz) NMR spectroscopic data (CDCl_3_) in Tables 1, 2, respectively; HRESIMS at *m/z* 511.3399 [M + Na]^+^ (calcd. for C_30_H_48_O_5_Na, 511.3394).

#### 2.4.7 Neritriterpenol N (**7**)

White amorphous powder; [*α*] +10.5 (*c* 0.3, MeOH); ν_max_ (KBr) 3454, 2975, 2933, 2871, 1670, 1645, 1383, 1267, 1233, and 1188 cm^−1^; CD (*c* 0.02, MeOH) 219 (+10.54) and 271 (+16.17) nm; ^1^H- (500 MHz) and ^13^C- (125 MHz) NMR spectroscopic data (CDCl_3_) in Tables 1, 2, respectively; HRESIMS at *m/z* 515.3734 [M − H]^−^ (calcd. for C_32_H_51_O_5_, 515.3742).

#### 2.4.8 11-Oxo-kansenonol (**8**)

White amorphous powder; [*α*] +18.5 (*c* 0.4, MeOH); ν_max_ (KBr) 3412, 2970, 2360, 2341, 1668, and 1034 cm^−1^; CD (*c* 0.02, MeOH) 215 (+11.96) and 276 (+21.4) nm; ^1^H- (500 MHz) and ^13^C- (125 MHz) NMR spectroscopic data (CDCl_3_) in Tables 1, 2, respectively; HRESIMS *m/z* 469.3323 [M - H]^-^ (calcd. for C_30_H_45_O_4_, 469.3312).

### 2.5 X-ray crystallographic data for compounds 3 and 5

The colorless crystals of neritriterpenols J (**3**) and L (**5**) were measured using a Brucker D8 VENTURE single-crystal X-ray diffractometer equipped with a dual microfocus X-ray source on Mo–Kα radiation.

#### 2.5.1 Neritriterpenol J (**3**)

Crystallographic data on neritriterpenol J (**3**): C_30_H_52_O_5_; crystal size: 0.56 × 0.10 × 0.02 mm^3^; wavelength (*λ*) = 0.71073 Å; crystal system: monoclinic; space group P21; unit cell dimensions, *a* = 12.0641 (5) Å [α = 90°], *b* = 7.0406 (2) Å [β = 94.7800 (10)°], and *c* = 17.1863 (7) Å [γ = 90°]; volume = 1,454.70 (9) Å^3^; *Z* = 2; density (calculated) = 1.125 Mg/m^3^; absorption coefficient = 0.074 mm^-1^; F (000) = 544 and T = 200 (2)K. A total of 27,930 reflections were collected, of which 5,130 independent reflections [R_int_ = 0.0678] with *I* > *2σ* (I) were used for the analysis. The final indices were *R*1 = 0.0392 and *wR*2 = 0.0878 with goodness-of-fit = 1.059. The absolute structure parameter is 0.1 (10). All data are shown in Supplementary Table S3. The crystallographic data were deposited in the Cambridge Crystallographic Data Centre (CCDC) under the deposition number 2208741.

#### 2.5.2 Neritriterpenol L (**5**)

Crystallographic data on neritriterpenol L (**5**): C_30_H_50_O_6_; crystal size: 0.60 × 0.08 × 0.03 mm^3^; wavelength (*λ*) = 0.71073 Å; crystal system: orthorhombic; space group P212121; unit cell dimensions, *a* = 6.9896 (3) Å [α = 90°], *b* = 12.3010 (6) Å [β = 90°], and *c* = 33.8198 (17) Å [γ = 90°]; volume = 2,907.8 (2) Å^3^; *Z* = 4; density (calculated) = 1.157 Mg/m^3^; absorption coefficient = 0.079 mm^-1^; F (000) = 1,112 and T = 200 (2)K. A total of 15,236 reflections were collected, of which 5,135 independent reflections [R_int_ = 0.0676] with *I* > *2σ* (I) were used for the analysis. The final indices were *R*1 = 0.0513 and *wR*2 = 0.0963 with the goodness-of-fit = 1.054. The absolute structure parameter is −1.0 (14). All data are shown in Supplementary Table S4. The crystallographic data were deposited in the Cambridge Crystallographic Data Centre (CCDC) under the deposition number 2208742.

### 2.6 Mo_2_(OAc)_4_-modified circular dichroism analysis

The determination of the absolute configuration of cyclic and acyclic *vic*-diols was achieved by using a transition metal-chelating reagent, dimolybdenum tetraacetate [Mo_2_(OAc)_4_]. The tested compounds (each compound, 0.5 mg) were directly dissolved in a solution of Mo_2_(OAc)_4_ complex in DMSO (5 mg/10 mL) in a molar ratio of Mo_2_(OAc)_4_/compound of about 1:0.5, and the mixture was subsequently measured for the induced CD spectra without the preparation and isolation of the complexes.

### 2.7 DP4+ probability analysis of computational analysis

The conformers discovered utilizing the GMMX package implemented in Gaussian 16 (Frisch et al., 2019) with the MMFF94 force field and conformers with an energy window of 5 kcal/mol were subjected for geometry optimizations and frequency calculations in the gas phase at the B3LYP/6–31G(d) level. The NMR chemical shifts were computed in MeOH or CHCl_3_ using the gauge-independent atomic orbital (GIAO) method at the PCM/mPW1PW91/6–31G+(d,p)//B3LYP/6–31G(d) level with the Boltzmann population refined in the solvation model based on density (SMD) for MeOH or CHCl_3_ at a new level (M06-2X/6–31G+(d,p)//B3LYP/6–31G(d)) (Zanardi et al., 2020). The Excel sheet provided by Grimblat et al. was utilized for DP4+ probability analysis (Grimblat et al., 2015).

### 2.8 Cell culture and viability assay

RAW 264.7 macrophages were purchased from American Type Culture Collection (ATCC, Rockville, MD, United States). Cells were cultured in Dulbecco’s modified Eagle’s medium supplemented with 10% heat-inactivated fetal bovine serum (FBS) plus 1% penicillin and streptomycin at 37°C with 5% CO_2_ in a humidified incubator. Cells were plated at a density of 5 × 10^4^ cells per well in 96-well plates 24 h before treatment of lipopolysaccharides (LPS, 1 μg/mL) with a vehicle or indicated compounds with doses of 5, 10, or 20 μM. After 24 h of treatment, cell viability was evaluated by MTT assay.

### 2.9 Determination of TNF-α and IL-6 secretion

RAW 264.7 cells were seeded in 24-well plates at a density of 2 × 10^5^ cells/well and pre-incubated for 24 h. Then, cells were treated with LPS (1 μg/mL) alone, LPS plus dexamethasone (DEX, 0.25 µM), or LPS plus various doses of indicated compounds (5, 10, and 20 µM) for 24 h. The conditioned medium was collected for the determination of TNF-α and IL-6 levels using ELISA kits (IL-6 ELISA Kit, BD, Franklin Lakes, NJ, United States; TNF-α ELISA Kit, Invitrogen, Carlsbad, CA, United States), according to the manufacturer’s instructions.

### 2.10 Statistical analyses

The data presented are representative of at least three independent experiments and expressed as the mean ± standard deviations (SD). All statistical analyses were performed using Prism 7.0 (GraphPad Software, United States) for a one-way ANOVA, followed by Tukey’s multiple range tests if ANOVA was significant (*p < 0.05, **p < 0.01, and ***p < 0.001).

## 3 Results and discussion

The ethanolic extract of the *E. neriifolia* L. stem was suspended in H_2_O and then sequentially partitioned with hexanes and CH_2_Cl_2_ to obtain two organic layers and an aqueous layer. The CH_2_Cl_2_ extract was chromatographed on a C_18_ gel flash column and then on a Sephadex LH-20 column. The subfractions were further subjected to preparative reverse-phase HPLC to yield eight compounds (**1**–**8**) (Figure 1). All isolated compounds were screened for their anti-inflammatory activities on the suppression of IL-6 and TNF-α in LPS-stimulated RAW 264.7 macrophages.

### 3.1 Structural elucidation of the isolated compounds

**FIGURE 2:**
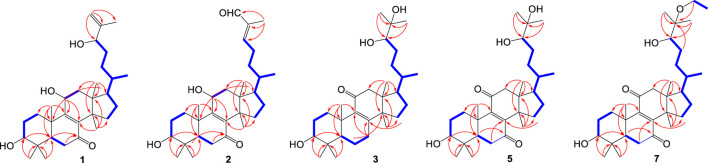
^1^H–^1^H COSY and key HMBC correlations of compounds **1**–**3**, **5**, and **7**.

Compound **1** was obtained as white amorphous powders with [*α*] +28.9 (*c* 0.1, MeOH), and its molecular formula C_30_H_48_O_4_ on par with seven degrees of unsaturation (DOU) was deduced from the HRESIMS pseudomolecular ion peak at *m/z* 495.3451 [M + Na]^+^ (calcd for C_30_H_48_O_4_Na^+^, 495.3445). The IR spectrum showed absorption bands, indicating the existence of hydroxyl (3429 cm-^1^), alkane (2927 cm^-1^), carbonyl (1672 cm^-1^), methyl (1465 and 1376 cm^-1^), and C‒O (1037 cm^-1^) functional groups. The ^13^C- and DEPT-NMR spectra of **1** separated the 30 carbons into seven methyl (*δ*_C_ 15.2, 16.3, 17.6, 18.9, 19.7, 25.7, and 27.6), eight methylene (*δ*_C_ 27.4, 27.8, 31.0, 31.3, 31.8, 33.7, 35.9, and 42.8), one sp2 methylene (*δ*_C_ 111.0), three methine (*δ*_C_ 35.6, 48.5, and 49.3), three oxymethine (*δ*_C_ 68.1, 76.2, and 78.3), four aliphatic quaternary carbon (*δ*_C_ 39.1, 39.6, 46.2, and 48.0), and three sp2 quaternary carbon (*δ*_C_ 140.4, 161.2, and 147.8) groups, and one carbonyl (*δ*_C_ 200.1) group, while the ^1^H-NMR spectrum demonstrated obviously seven methyl [*δ*_H_ 0.74 (s), 0.90 (d, *J* = 6.5 Hz), 0.94 (s), 1.02 (s), 1.17 (s), 1.28 (s), and 1.79 (s)] and three oxymethylene [*δ*_H_ 3.34 (m), 4.06 (t, *J* = 7.0 Hz), and 4.72 (t, *J* = 8.5 Hz)] groups, and one olefinic methylene [*δ*_H_ 4.88 (brs) and 4.97 (brs)] group. The aforementioned data indicated that **1** possesses a symbolic tetracyclic-fused ring moiety containing seven methyl groups, three hydroxyl groups, a conjugated double-ketone system, and a terminal olefin. According to the C‒H single-bond correlations of **1** by the HSQC spectrum, the ^1^H- and ^13^C-NMR assignments of **1** were determined (Tables 1, 2). The planar structure of **1** was elucidated by ^1^H–^1^H COSY and HMBC spectra (Figure 2): a 3,11-diol-8-en-7-one-containing 6/6/6/5-fused ring was established by the ^1^H–^1^H COSY correlations of H_2_-1 (*δ*_H_ 1.58 and 2.47)/H_2_-2 (*δ*_H_ 1.78, 2H)/oxymethine H-3 (*δ*_H_ 3.34), H-5 (*δ*_H_ 1.68)/H_2_-6 (*δ*_H_ 2.44 and 2.50), oxymethine H-11 (*δ*_H_ 4.72)/H_2_-12 (*δ*_H_ 1.83 and 2.43), and H_2_-15 (*δ*_H_ 1.46 and 2.12)/H_2_-16 (*δ*_H_ 1.35 and 1.96)/H-17 (*δ*_H_ 1.62), as well as the HMBC correlations from Me-28 (*δ*_H_ 0.94) and Me-29 (*δ*_H_ 1.02) to oxymethine C-3 (*δ*_C_ 78.3), C-4 (*δ*_C_ 39.1), and C-5 (*δ*_C_ 49.3); from Me-19 (*δ*_H_ 1.28) to C-1 (*δ*_C_ C 33.7), C-5, olefinic quaternary carbon C-9 (*δ*_C_ 161.2), and C-10 (*δ*_C_ 39.6); from Me-18 (*δ*_H_ 0.74) to C-12 (*δ*_C_ 42.8), C-13 (*δ*_C_ 46.2), C-14 (*δ*_C_ 48.0), and C-17 (*δ*_C_ 48.5); from Me-30 (*δ*_H_ 1.17) to olefinic quaternary carbon C-8 (*δ*_C_ 140.4), and C-13, C-14, and C-15 (*δ*_C_ 31.8); and from H-5 (*δ*_H_ 1.68) and H_2_-6 (*δ*_H_ 2.44 and 2.50) to carbonyl C-7 (*δ*_C_ 200.1). Then, a 24-diol-25-ene-contained C8-side chain attached at C-17 was connected by the ^1^H–^1^H COSY correlations of H-17 (*δ*_H_ 1.62)/H-20 (δ_H_ 1.51)/Me-21 (*δ*_H_ 0.90)/H_2_-22 (*δ*_H_ 1.24 and 1.60)/H_2_-23 (*δ*_H_ 1.49 and 1.61)/oxymethine H-24 (*δ*_H_ 4.06) and the HMBC correlations from olefinic methylene H2–26 (*δ*_H_ 4.88 and 4.97) and Me-27 (*δ*_H_ 1.79) to oxymethine C-24 (*δ*_C_ 76.2) and olefinic quaternary carbon C-25 (*δ*_C_ 147.8).

**FIGURE 3:**
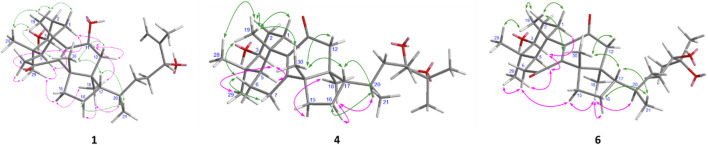
Selected NOESY correlations of compounds **1**, **4**, and **6**. (green arrow: α-orientation; pink arrow: *ß*-orientation).

**FIGURE 4:**
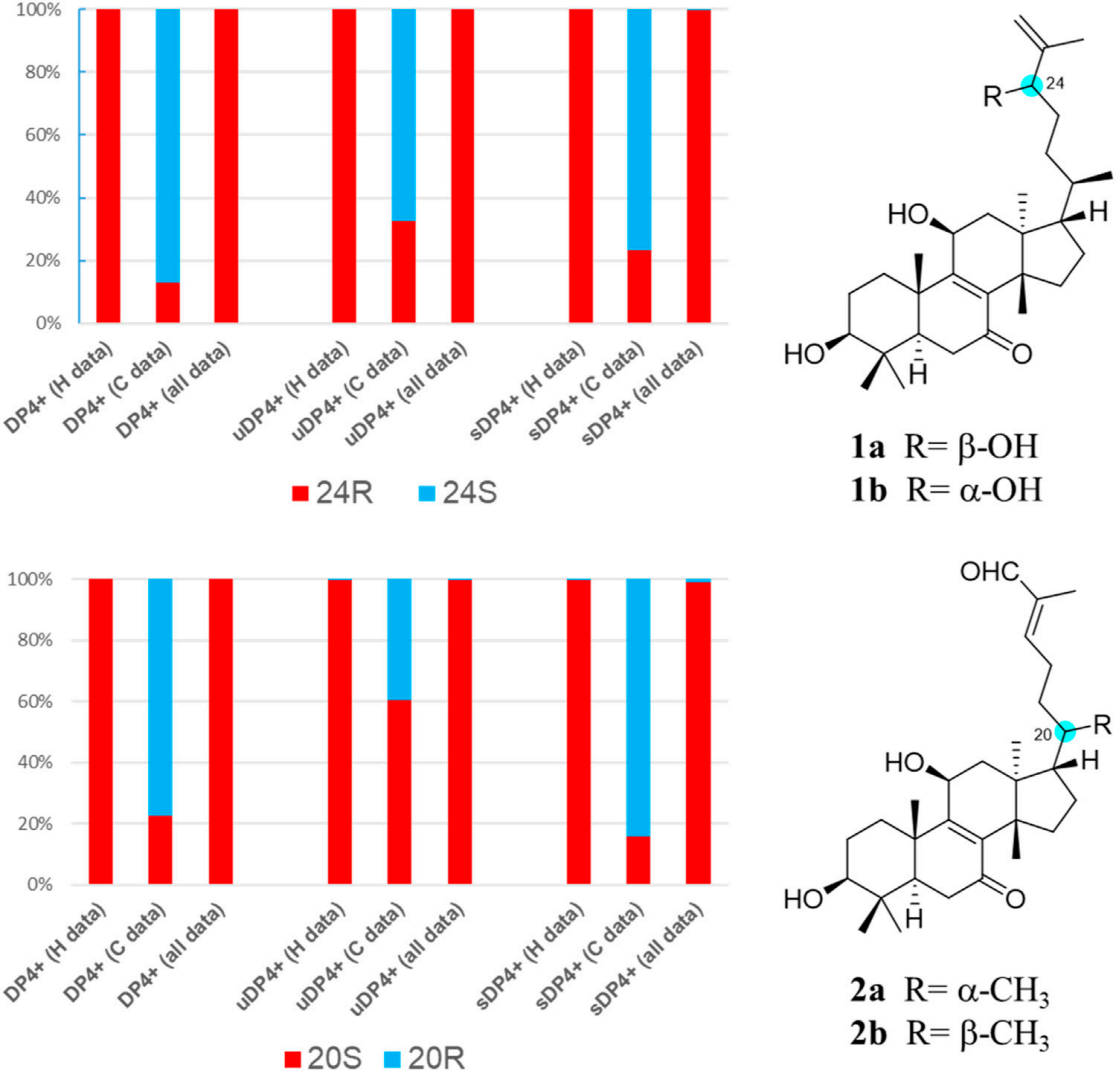
DP4+ probability for compounds **1** and **2** at the mPW1PW91/6–31G+(d,p) level.

The planar framework of **1** indicates that compound **1** belongs to the lanostane- or euphane-type triterpene, a pair of stereoisomers having two different conformations at C-13 and C-14 in a rigid Δ^8,24^–6/6/6/5-tetracyclic triterpene (lanostane has a 13*R*,14*R*-orientation, while euphane is the opposite 13*S*,14*S*-direction), and the chirality of these two chiral carbons cannot be determined solely by the NOE correlation between Me-18 and Me-30. Based on the regular conformations of lanostane and euphane triterpenoids, the anti-parallel relationship between H-5 (α-oriented) and Me-19 (β-oriented) was established, whereas the *ß*-configuration of H-17 was assigned unequivocally. In the NOESY spectrum of 1 (Figure 3), the *ß*-orientations for H_eq_-1, H_ax_-2, H_ax_-12, H-17, H_eq_-16, Me-19, Me-21, Me-28, and Me-30 were determined by the corrections of Me-19/Me-28, Me-19/Me-30, Me-19/H_eq_-1 (*δ*_H_ 2.47), Me-19/H_ax_-2 (*δ*_H_ 1.78), Me-28/H_ax_-2, H_eq_-1/H_ax_-2, Me-30/H_ax_-12 (*δ*_H_ 2.43), Me-30/H-17, H_eq_-16 (*δ*_H_ 1.96)/Me-21, and H-17/Me-21. Meanwhile, the NOE correlations of Me-29/H-3, Me-29/H-5, Me-29/H_eq_-6 (*δ*_H_ 2.44), H-3/H-5, H-3/H_ax_-1 (*δ*_H_ 1.58), H_ax_-1/H-11, H-11/H_eq_-12 (*δ*_H_ 1.83), H-11/Me-18, Me-18/H_ax_-15 (*δ*_H_ 1.46), Me-18/H_ax_-16 (*δ*_H_ 1.35), Me-18/H-20, and H_ax_-15/Me-29 indicated their inverse α-orientations for H_ax_-1, H-3, H-5, H_eq_-6, H-11, H_eq_-12, Me-18, H_ax_-15, H_ax_-16, H-20, and Me-29. Of the complete configuration assignment, the *ß*-form of Me-30 and the α-form of Me-18 illustrated the 13*S*,14*S*-chirality of **1**, and thus, compound **1** was recognized as a euphane-type triterpene. Likewise, the *ß*-orientations of OH-3 and OH-11 in **1** were decided by the downward orientation of both H-3 and H-11 on the rigid tetracyclic moiety. The spatial configuration of 24-OH at C-24 was resolved as an *R*-form configuration by comparison of chemical shifts with those of known compounds, namely, (24*S*/*R*)-eupha-8,25-dien-3β,24-diol-7-one (Xu et al., 2009; Chang et al., 2022) and 3β,11α,24*S*/*R*-trihydroxylanosta-8,25-dien-7-one (Shi et al., 2020) (SupplementaryTable S1). Furthermore, the configuration of C-24 was further determined by *in silico* GIAO NMR calculations (Grimblat et al., 2015; Zanardi et al., 2020) at the mPW1PW91/6–31G+(d,p) level on two possible forms of **2a** (24*R*) and **2b** (24*S*). The DP4+ calculated results showed that the probability of C-24*R* is 100% (Figure 4). Taken together, compound **1** was determined as a structure of (24*R*)-3β,11β,24β-trihydroxy-eupha-8,25-dien-7-one, named neritriterpenol H.

Neritriterpenol I (**2**) has a molecular formula C_30_H_46_O_4_ with eight DOU established from its HRESIMS ion peak at *m/z* 493.3287 [M + Na]^+^ (calcd. for C_30_H_46_O_4_Na^+^, 493.3288), and the ring carbon signals in ^13^C-NMR data (Table 2) were almost identical compared with those of **1**, implying that **2** had the same planar tetracarbocyclic framework of **1**. The IR absorption bands of **2** expressed the functional groups of hydroxyl (3442 cm^-1^), carbonyl (1716 cm^-1^), and olefin (1660 cm^-1^) groups, and the ^1^H–^1^H COSY and HMBC correlations (Figure 2) revealed that **2** has the same planar 3,11-dihydroxyl-8-en-7-one tetracyclic moiety as **1**, and the only difference is on the side-chain. According to the ^13^C- and DEPT-NMR spectra of **2**, the C8-side chain of **2** was assembled by two methyl (*δ*_C_ 9.3 and 18.7) and two methylene (*δ*_C_ 26.0 and 34.0) groups, and one aliphatic methine (*δ*_C_ 35.6), one sp2 methine (*δ*_C_ 154.9), one sp2 quaternary carbon (*δ*_C_ 139.3), and one aldehyde (*δ*_C_ 195.3) group. The combination of the COSY fragment of H-17/Me-21/H-20/H_2_-22/H_2_-23/H-24 and the HMBC correlations from Me-27 to olefinic methine C-24, olefinic quaternary carbon C-25, and aldehyde C-26 (Figure 2) provided this C8-side chain a 2-methyl-6λ^3^-hept-2-enal unit, attached at C-17. Noteworthily, the ^1^H-NMR chemical shift (*δ*_H_ 0.95, d, *J* = 6.5 Hz) of Me-21, the negative optical rotation (−8.3°), and the NOESY correlations of **2** (Supplementary Figure S1) showed a different geometry of the tetracyclic moiety as **1**, indicating that **2** belongs to the tirucallane skeleton, a 20*S*-stereoisomer of the euphane triterpenoid. The critical direction of Me-21 was further confirmed by DP4+ probability calculations at the mPW1PW91/6–31G+(d,p) level on two possible poses, the isomers of C-20, **2a** (20*S*) and **2b** (20*R*), and the calculated results suggested that **2a** is the favorite structure by its almost 100% probability distribution term (Figure 4). Thus, the structure of compound **2** was elucidated to be (5*R*,10*S*,13*S*,14*S*,17*S*,20*S*)-3β,11β-dihydroxytirucalla-8,24*E*-dien-7,26-dione.

As far as **3** (neritriterpenol J) and **4** (neritriterpenol K) were concerned, these two compounds were considered to be a pair of stereoisomers because of their extreme resemblance of ^1^H- and ^13^C-NMR data (Tables 1, 2), and the same molecular formula C_30_H_50_O_4_ (six DOU) deduced from the HRESIMS ion peaks of **3** and **4** at *m/z* 497.3618 [M + Na]^+^ (calcd. for C_30_H_50_O_4_Na, 497.3601) and 473.3638 [M − H]^−^ (calcd. for C_30_H_49_O_4_, 473.3265), respectively. Analyzing the ^13^C- and DEPT-NMR spectra of **3** and **4**, both of their 30 carbons were found to be assembled by eight methyl, nine methylene, three aliphatic methine, two oxymethine, four aliphatic quaternary carbon, one oxygenated quaternary carbon, and two olefinic quaternary carbon groups, as well as one carbonyl group, revealing that **3** and **4** are also triterpenoids. Then, the juxtaposition of the ^1^H- and ^13^C-NMR data on **3** and **1** revealed several common features in these two compounds, a hydroxyl group [*δ*_H_ 3.27 (dd, *J* = 11.0, 5.5 Hz); *δ*_C_ 78.9], a tetrasubstituted double bond (*δ*_C_ 161.5 and 139.6), a conjugated ketone (*δ*_C_ 199.1), and a multi-methyl group, suggesting that compound **3** has a similar unsaturated conjugated 6/6/6/5-fused ring moiety to **1**. The 6/6/6/5-fused tetracyclic ring of **3** is estimated to be an eupha-8-en-3-ol-11-one moiety through the HMBC correlations (Figure 2) Me-18 (*δ*_H_ 0.93) to C-12 (*δ*_C_ 51.1), C-13 (*δ*_C_ 44.7), C-14 (*δ*_C_ 51.2), and C-17 (*δ*_C_ 50.3); from Me-19 (*δ*_H_ 1.22) to C-1 (*δ*_C_ 34.1), C-5 (*δ*_C_ 51.7), C-9 (*δ*_C_ 139.6), and C-10 (*δ*_C_ 37.1); from Me-28 (*δ*_H_ 0.85) and Me-29 (*δ*_H_ 1.06) to oxymethine C-3 (*δ*_C_ 78.9), C-4 (*δ*_C_ 39.0), and C-5; and from Me-30 (*δ*_H_ 1.04) to C-8 (*δ*_C_ 161.5), C-13, C-14, and C-15 (*δ*_C_ 30.0). Moreover, the 24,25-diol-containing side chain attached at C-17 was illustrated by the HMBC correlations from Me-26 (*δ*_H_ 1.18) and Me-27 (*δ*_H_ 1.24) to oxymethine C-24 (*δ*_C_ 79.3) and oxygenated quaternary carbon C-25 (*δ*_C_ 73.2). Similar ^1^H–^1^H COSY and HMBC correlations for **4** (Figure 2) illustrated the same planar structure as **3**, confirming their stereoisomeric relationships.

**FIGURE 5:**
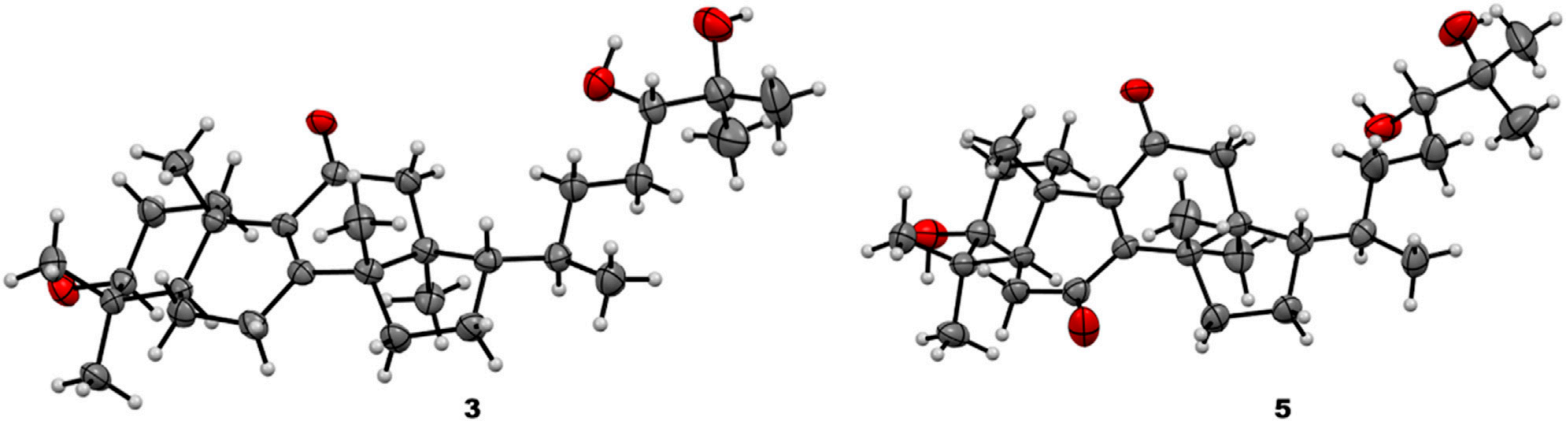
X-ray crystallographic diagrams of compounds **3** and **5**.

**FIGURE 6:**
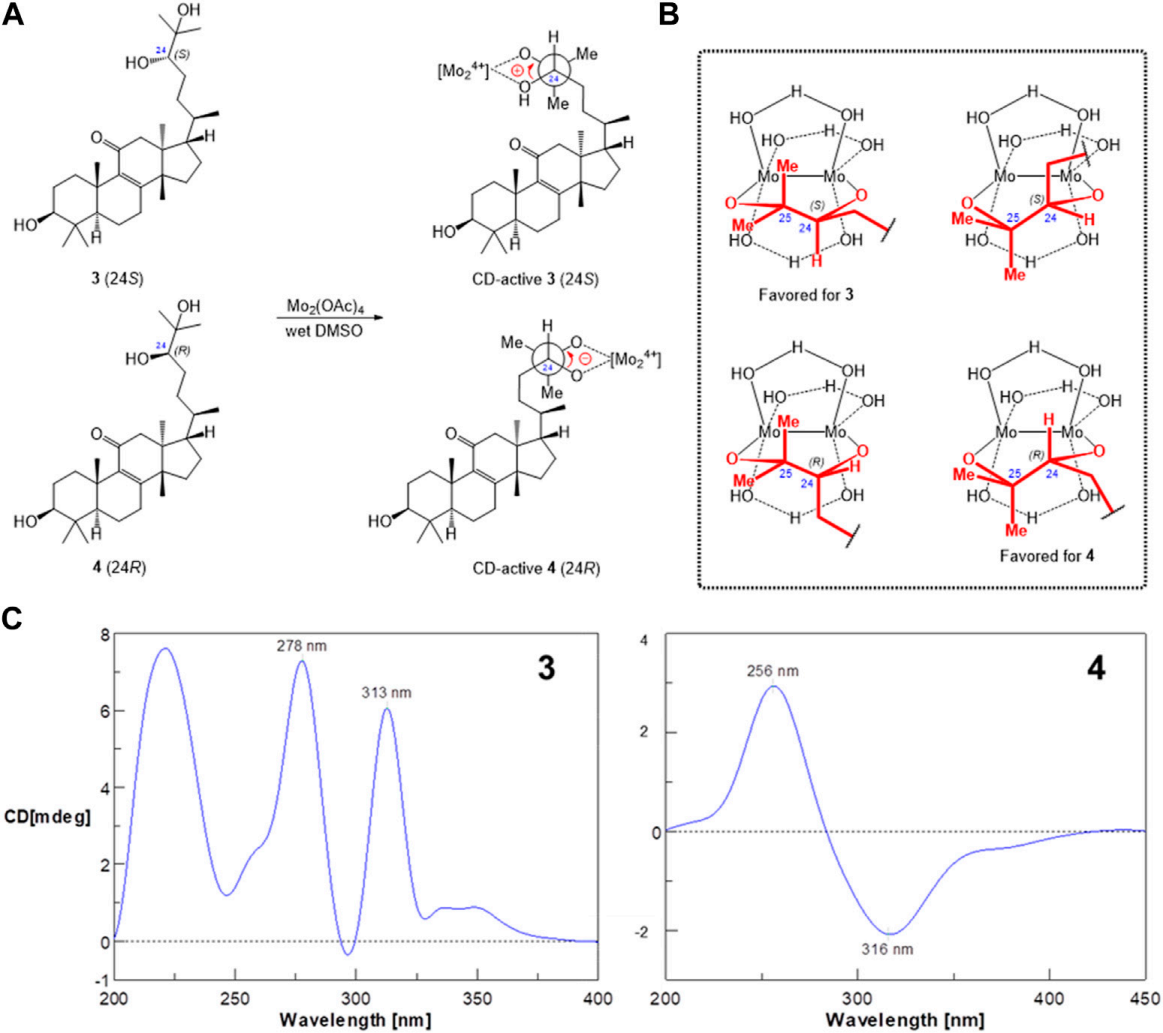
[Mo_2_(OAc)_4_]-induced circular dichroism spectra of the CD active complexes of compounds **3** and **4**. **(A)** Reaction of [Mo_2_(OAc)_4_] in DMSO solution. **(B)** Model structures of the [Mo_2_(OAc)_4_] complex. **(C)** CD spectra of the [Mo_2_(OAc)_4_] complex.

Since **3** and **4** were considered a pair of stereoisomers, some obviously different chemical shifts were found in C-21 to C-24 by the alignment of their ^1^H-NMR data (Supplementary Figure S2), indicating that the substitution of the OH group makes C-24 the only different chiral center. There was no recognizable NOE correlation to identify the 24*R* and 24*S* configurations. Fortunately, colorless crystals of **3** were successfully yielded from natural evaporation of **3**-dissolved methanol/chloroform (1:1) solution for the configuration determination by X-ray crystallographic analysis (Figure 5), and compound **3** is perspicuously identified as (5*R*,10*S*,13*S*,14*S*,17*S*,20*R*,24*S*)-eupha-8-en-3β,24β,25-triol-11-one, whereas compound **4** is thus a 24*R*-epimer having a 24β-hydroxyl group. The 24*R*/*S* configuration was further confirmed by the dimolybdenum tetraacetate [Mo_2_(OAc)_4_]-induced circular dichroism (CD) method for the rapid configuration diagnosis of the acyclic 24,25-*sec,tert*-diols (Di Bari et al., 2004) in euphane-type triterpenoids (Figure 6A). Figure 6A shows both *R*/*S*-epimers **3** and **4** can be chelated by Mo_2_(OAc)_4_ in the aqueous DMSO solution to form complexes of Mo_2_(OAc)_4_ with 1,2-diol-containing molecules, and one complex has two envisaged ligation poses, the “equatorial” (major) and “axial” (minor) positions of bulk with side-chain residues in the diol−dimolybdenum adducts (Figure 6B). Importantly, the absorption region of the dimolybdenum chromophore in the CD spectrum is obtained above 250 nm, while the CD band around 305 nm correlates with the absolute configuration of the diol substrate. Therefore, the positive (313 nm) and negative (316 nm) Cotton effects observed in the ICDs (Figure 6C) of **3** and **4**, respectively, indicated the absolute configurations of C-24 in **3** and **4**, which were 24*S* and 24*R*, respectively.

Similarly, the sodium adduct ion peaks at *m/z* 511.3397 [M + Na]^+^ and *m/z* 511.3399 [M + Na]^+^ (calcd. for C_30_H_48_O_5_Na, 511.3394) in the HRESIMS spectra of **5** and **6** deduced the same molecular formula, C_30_H_48_O_5_ (7 DOU), indicating that compounds **5** and **6** are a couple of stereoisomers. Additionally, their ^1^H- and ^13^C-NMR data (Tables 1, 2) were extremely close to those of **3** and **4** (Supplementary Figure S2), indicating that the relationship between **5** and **6** is the same as **3** and **4**, and these two pairs are homologs. In the ^13^C-NMR data on **5**/**6** and **3**/**4**, the most prominent difference is that a methylene (*δ*_C_ 29.7) is oxidized into a ketone group (*δ*_C_ 199.9/200.0) in **5** and **6**. Furthermore, the HMBC corrections from H_2_-6 (**5**: *δ*_H_ 2.44 and 2.50; **6**: *δ*_H_ 2.45 and 2.55) to ketone C-7 (*δ*_C_ 199.9/200.0) illustrated the planar eupha-8-en-3,24,25-triol-7,11-dione of **5** and **6**. The same chirality of the rigid ring moiety of **5** and **6** was decided by their NOESY corrections, whereas the orientations of 24,25-*sec,tert*-diols in **5**/**6** were recognized by the X-ray crystallographic analysis of **5** (Figure 5). The chirality of C-24 of **6** was decided to be in the *R* form by the comparison of chemical shifts with those of known compounds, neritriterpenols A, B, and G (Chang et al., 2022) and ganodermanondiol (Fujita et al., 1986; Arisawa et al., 1988) (Supplementary Table S2), as well as the similar [Mo_2_(OAc)_4_]-induced CD results of **5** and **6** to those of **3** and **4**. Finally, the NMR comparison allocated compound **5** in 24*S*-chirality, while **6** in 24*R*-distribution. Compounds **5** and **6** were named neritriterpenols L and M, respectively.

Compound **7**, obtained as white amorphous powder, possesses a molecular formula C_32_H_52_O_4_ (7 DOU), according to the HRESIMS pseudomolecular ion at *m/z* 515.3734 [M − H]^−^ (calcd. for C_32_H_51_O_5_, 515.3742). This compound was presumed to be an alkylated derivative of **5** and **6**, an ethanoxylated C-25 of **5** and **6**, by the observation of the ^1^H–^1^H COSY correlations of H_2_-1' (*δ*_H_ 3.45)/Me-2' (*δ*_H_ 1.18) and the HMBC correlations from H_2_-1′ to the C-25 (*δ*_C_ 77.4) (Figure 2). Moreover, the *S* configuration of the chiral center C-24 of compound **7** was decided by the coupling constant of H-24 (*δ*_H_ 3.42, dd, *J* = 2.5 and 9.0 Hz) that is close to those of 3 (*δ*_H_ 3.30, dd, *J* = 2.0, and 10.5 Hz) and 5 (*δ*_H_ 3.25, dd, *J* = 2.0, and 10.0 Hz), revealing that **7** has the same stereochemistry as neritriterpenol L (**5**) and named neritriterpenol N.

As for compound **8**, it was considered a known triterpenoid, 11-oxo-kansenonol, determined by the comparison of previously reported data including NMR, HRESIMS, UV, and optical rotation (Wang et al., 2003).

### 3.2 Evaluation of the anti-inflammatory activity of the isolated triterpenes

**FIGURE 7:**
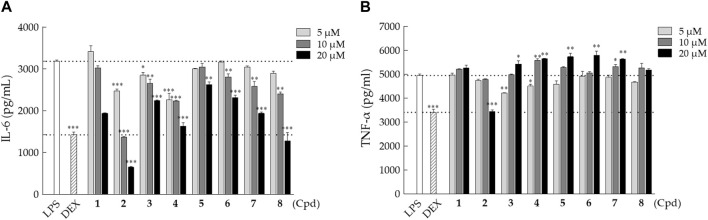
Inhibitory effects of compounds **1**–**8** on IL-6 and TNF-α secretion in LPS-stimulated RAW 264.7 macrophages. RAW 264.7 cells were co-treated with LPS (1 μg/mL) and various doses of indicated compounds (5, 10, or 20 µM) for 24 h. **(A)** IL-6 and **(B)** TNF-α concentrations were measured using the ELISA kit. The values presented are the mean ± SD of three independent experiments and were analyzed using one-way ANOVA with Tukey’s *post hoc* test (*p < 0.05, **p < 0.01, and ***p < 0.001).

The anti-inflammatory activity of the isolated triterpenes **1**‒**8** was tested by evaluating the secretion of pro-inflammatory cytokines, IL-6 and TNF-α, in the lipopolysaccharide (LPS)-stimulated macrophages. RAW 264.7 macrophages were treated with LPS and different concentrations of compounds **1**–**8** (5, 10, and 20 μM) for 24 h, followed by the conditioned medium collection. Cytotoxicity analysis showed that the isolated compounds have little to no effect on cell viability with the doses of 5, 10, and 20 μM in macrophages. All isolated compounds (**1**–**8**) exhibited inhibitory activity against IL-6 secretion in a dose-dependent manner in LPS-stimulated macrophages (Figure 7A). Among them, compound **2** showed the strongest inhibitory activity on the suppression of LPS-induced IL-6 secretion. On the other hand, most isolated compounds (**1** and **3**–**8**), except compound **2**, showed weak or no activity on the suppression of LPS-stimulated TNF-α secretion in RAW 264.7 macrophages (Figure 7B). Only 20 μM of compound **2** showed a remarkable inhibitory effect on TNF-α secretion in LPS-stimulated cells similar to the DEX group.

Although it has been reported that there is a close correlation between TNF-α and IL-6 in several inflammation-related diseases, IL-6 and TNF-α play their individual roles and functions under a given circumstance (Kany et al., 2019; Wang and He, 2020). Therefore, the discovery of specific IL-6 inhibitors that effectively diminish the level of IL-6 but not TNF-α should be useful to develop into drugs with less unfavorable side effects. The currently available IL-6-specific inhibitors are monoclonal antibodies, such as tocilizumab that targets the IL-6 receptor blocking IL-6 signaling (Kaye and Siegel, 2020), while small-molecule IL-6 inhibitors are few. In the present study, we isolated and elucidated euphane-type triterpenes (**1** and **3**–**8**) from the titled plant that possess potent pharmacological activity specifically on the suppression of IL-6 but not TNF-α, an encouragement to develop into more effective IL-6-specific inhibitors. On the other hand, javamide-II identified from coffee also showed a similar inhibitory activity against IL-6 but not TNF-α and IL-1β in macrophage-like THP-1 cells (Park et al., 2020). It is known that dysregulated IL-6 could be the major cause of several diseases, for example, rheumatoid arthritis and COVID-19. Moreover, several lines of evidence have revealed that elevated IL-6 is correlated with the duration and/or severity of COVID-19 in light of the fact that FDA approved the use of IL-6 inhibitory monoclonal antibodies (ex. tocilizumab) for COVID-19 patients, who have systemic inflammation. As a result, searching and developing new and more effective IL-6-specific inhibitors are critical as it meets the unmet medical need (Stenvinkel et al., 2005; Zhou et al., 2020; Rubin et al., 2021). Our results should have opened up a new avenue toward developing new medicines that are in a position to modulate IL-6 and/or TNF-α.

## 4 Conclusion

In our continuous search for anti-inflammatory components from the ethanolic extract of *E. neriifolia* L., we successfully isolated and identified eight 6/6/6/5-fused triterpenes, including six new euphane-type triterpenes (compounds **1** and **3**–**7**) and a tirucallane-type triterpene (compound **2**), as well as a known compound, 11-oxo-kansenonol (**8**). Based on the anti-inflammatory assay, euphanes **3**–**8** showed selective inhibition on IL-6 in a dose-dependent manner with little or no effect on TNF-α in contrast to tirucallane **2** that exhibited strong inhibition against both IL-6 and TNF-α. It has been well-documented that dysregulated IL-6 may lead to over-reacted immune responses and thus results in unfavorable consequences. Suppressing IL-6 apparently is a good strategy to cope with dysregulated IL-6-associated diseases such as rheumatoid arthritis. Given that TNF-α and IL-6 each has its own independent role, they, however, are often regulated together and function co-operatively in many aspects of immune responses. It would not be surprising that most IL-6 inhibitors reported up until now suppress IL-6 and TNF-α as a whole, as tirucallane **2** exemplifies here (Figures 7A,B). Despite the predicament, the newly isolated triterpenes from *E. neriifolia* L., especially compounds **4** and **8**, selectively inhibit the IL-6 dysregulated immune responses. In conclusion, our study contributes to the expanding knowledge of anti-inflammatory compounds derived from *E. neriifolia* L.

## Data Availability

The datasets presented in this study can be found in online repositories. The names of the repository/repositories and accession number(s) can be found at: https://www.ccdc.cam.ac.uk/-, 2208741 and 2208742.
