# Supplementary material for: Anti-inflammatory effect of euphane- and tirucallane-type triterpenes isolated from the traditional herb Euphorbia neriifolia L
Source: Front Chem. 2023 Jun 23;11:1223335. doi: 10.3389/fchem.2023.1223335 (PMC10326319; doi:10.3389/fchem.2023.1223335)
Supplement: Supplementary file 1 [file DataSheet1.PDF]

Supplementary Material

Table S1. Comparison of the ^{13}C-NMR data of C-20–C-27 between **a**, **b**, **c**, **d**, and **1**.
Table S2. Comparison of the ^{13}C-NMR data of C-20–C-27 between **e**, **f**, **g**, **h**, and **4–6**.
Table S3. Crystal data and structure refinement for neritriterpenol J (**3**).
Table S4. Crystal data and structure refinement for neritriterpenol L (**5**).
Figure S1. NOESY of correlations of compounds **2**, **3**, **5**, and **7**.
Figure S2. ^{1}H-NMR alignment of compounds **3–6** (A–D).
Figure S3. ^{1}H-NMR spectrum of neritriterpenol H (**1**) in $CDCl_3$ (500 MHz)
Figure S4. ^{13}C-NMR spectrum of neritriterpenol H (**1**) in $CDCl_3$ (125 MHz)
Figure S5. ^{1}H-^{1}H COSY spectrum of neritriterpenol H (**1**) in $CDCl_3$
Figure S6. HSQC spectrum of neritriterpenol H (**1**) in $CDCl_3$
Figure S7. HMBC spectrum of neritriterpenol H (**1**) in $CDCl_3$
Figure S8. NOESY spectrum of neritriterpenol H (**1**) in $CDCl_3$
Figure S9. HRESIMS spectrum of neritriterpenol H (**1**)
Figure S10. ^{1}H-NMR of neritriterpenol I (**2**) in $CDCl_3$ (500 MHz)
Figure S11. ^{13}C NMR of neritriterpenol I (**2**) in $CDCl_3$ (125 MHz)
Figure S12. ^{1}H-^{1}H COSY spectrum of neritriterpenol I (**2**) in $CDCl_3$
Figure S13. HSQC spectrum of neritriterpenol I (**2**) in $CDCl_3$
Figure S14. HMBC spectrum of neritriterpenol I (**2**) in $CDCl_3$
Figure S15. NOESY spectrum of neritriterpenol I (**2**) in $CDCl_3$
Figure S16. HRESIMS spectrum of neritriterpenol I (**2**)
Figure S17. ^{1}H-NMR spectrum of neritriterpenol J (**3**) in $CDCl_3$ (500 MHz)
Figure S18. ^{13}C-NMR spectrum of neritriterpenol J (**3**) in $CDCl_3$ (125 MHz)
Figure S19. ^{1}H-^{1}H COSY spectrum of neritriterpenol J (**3**) in $CDCl_3$
Figure S20. HSQC spectrum of neritriterpenol J (**3**) in $CDCl_3$
Figure S21. HMBC spectrum of neritriterpenol J (**3**) in $CDCl_3$
Figure S22. NOESY spectrum of neritriterpenol J (**3**) in $CDCl_3$
Figure S23. HRESIMS spectrum of neritriterpenol J (**3**)
Figure S24. ^{1}H-NMR spectrum of neritriterpenol K (**4**) in $CDCl_3$ (500 MHz)
Figure S25. ^{13}C-NMR spectrum of neritriterpenol K (**4**) in $CDCl_3$ (125 MHz)
Figure S26. ^{1}H-^{1}H COSY spectrum of neritriterpenol K (**4**) in $CDCl_3$
Figure S27. HSQC spectrum of neritriterpenol K (**4**) in $CDCl_3$
Figure S28. HMBC spectrum of neritriterpenol K (**4**) in $CDCl_3$
Figure S29. NOESY spectrum of neritriterpenol K (**4**) in $CDCl_3$
Figure S30. HRESIMS spectrum of neritriterpenol K (**4**)
Figure S31. ^{1}H-NMR spectrum of neritriterpenol L (**5**) in $CDCl_3$ (500 MHz)

Figure S32. ^{13}C-NMR spectrum of neritriterpenol L (**5**) in $CDCl_3$ (125 MHz)
Figure S33. ^{1}H-^{1}H COSY spectrum of neritriterpenol L (**5**) in $CDCl_3$
Figure S34. HSQC spectrum of neritriterpenol L (**5**) in $CDCl_3$
Figure S35. HMBC spectrum of neritriterpenol L (**5**) in $CDCl_3$
Figure S36. NOESY spectrum of neritriterpenol L (**5**) in $CDCl_3$
Figure S37. HRESIMS spectrum of neritriterpenol L (**5**)
Figure S38. ^{1}H-NMR spectrum of neritriterpenol M (**6**) in $CDCl_3$ (500 MHz)
Figure S39. ^{13}C-NMR spectrum of neritriterpenol M (**6**) in $CDCl_3$ (125 MHz)
Figure S40. ^{1}H-^{1}H COSY spectrum of neritriterpenol M (**6**) in $CDCl_3$
Figure S41. HSQC spectrum of neritriterpenol M (**6**) in $CDCl_3$
Figure S42. HMBC spectrum of neritriterpenol M (**6**) in $CDCl_3$
Figure S43. NOESY spectrum of neritriterpenol M (**6**) in $CDCl_3$
Figure S44. HRESIMS spectrum of neritriterpenol M (**6**)
Figure S45. ^{1}H-NMR spectrum of neritriterpenol N (**7**) in $CDCl_3$ (500 MHz)
Figure S46. ^{13}C-NMR spectrum of neritriterpenol N (**7**) in $CDCl_3$ (125 MHz)
Figure S47. ^{1}H-^{1}H COSY spectrum of neritriterpenol N (**7**) in $CDCl_3$
Figure S48. HSQC spectrum of neritriterpenol N (**7**) in $CDCl_3$
Figure S49. HMBC spectrum of neritriterpenol N (**7**) in $CDCl_3$
Figure S50. NOESY spectrum of neritriterpenol N (**7**) in $CDCl_3$
Figure S51. HRESIMS spectrum of neritriterpenol N (**7**)
Figure S52. ^{1}H-NMR spectrum of 11-oxo-kansenonol (**8**) in $CDCl_3$ (500 MHz)
Figure S53. ^{13}C-NMR spectrum of 11-oxo-kansenonol (**8**) in $CDCl_3$ (125 MHz)
Figure S54. ^{1}H-^{1}H COSY spectrum of 11-oxo-kansenonol (**8**) in $CDCl_3$
Figure S55. HSQC spectrum of 11-oxo-kansenonol (**8**) in $CDCl_3$
Figure S56. HMBC spectrum of 11-oxo-kansenonol (**8**) in $CDCl_3$
Figure S57. NOESY spectrum of 11-oxo-kansenonol (**8**) in $CDCl_3$
Figure S58. HRESIMS spectrum of 11-oxo-kansenonol (**8**) in $CDCl_3$

Table S1. Comparison of the ^{13}C-NMR data of C-20–C-27 between **a**, **b**, **c**, **d**, and **1**.

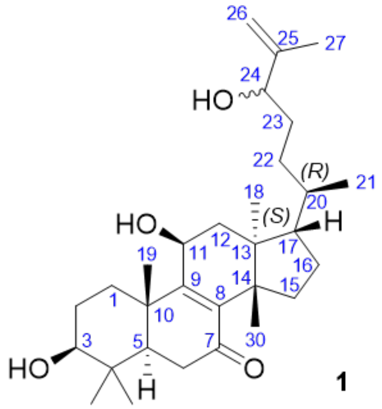

1

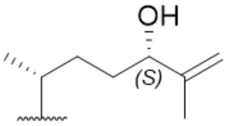

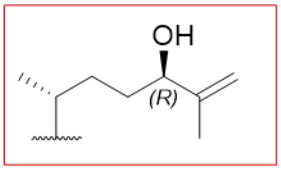

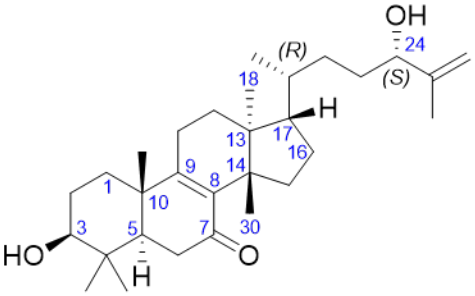

(24*S*)-eupha-8,25-dien-3*β*,24-diol-7-one (**a**)
J. Nat. Prod. **2009**, *72*, 1620-1626

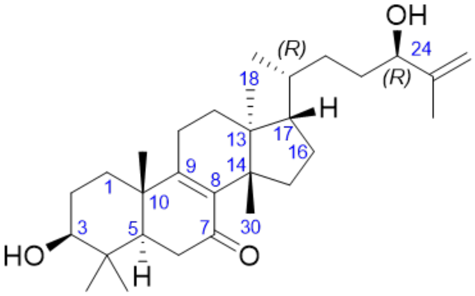

(24*R*)-eupha-8,25-dien-3£],24-diol-7-one (**b**)
J. Nat. Prod. **2017**, *80*, 1767-1775

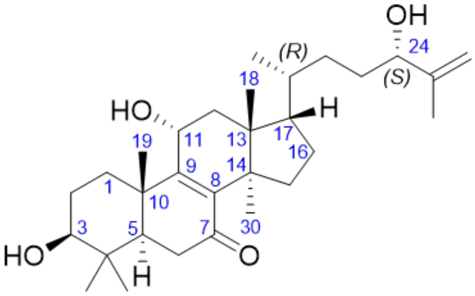

3*β*,11*α*,24*S*-trihydroxylanosta-8,25-dien-7-one (**c**)
Bioorg. Chem. **2020**, *100*, 103871

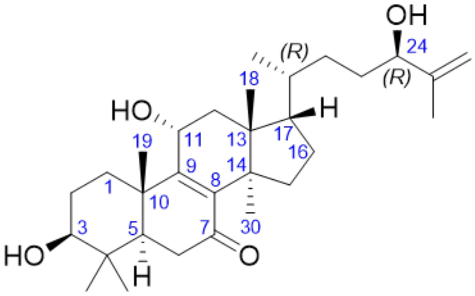

3*β*,11*α*,24*R*-trihydroxylanosta-8,25-dien-7-one (**d**)
Bioorg. Chem. **2020**, *100*, 103871

δ_C (ppm)	C-20	C-21	C-22	C-23	C-24	C-25	C-26	C-27
**a**	36.0	19.2	31.4	31.8	76.8	147.8	111.3	17.7
**b**	35.8	19.2	31.2	31.6	76.4	148.0	111.2	17.7
**c**	36.1	18.6	31.4	31.7	76.7	147.4	111.5	17.2
**d**	36.1	18.6	31.6	31.7	76.3	147.7	111.0	17.6
**1**	35.6	18.9	31.0	31.3	76.2	147.8	111.0	17.6

Table S2. Comparison of the ^{13}C-NMR data of C-20–C-27 between **e**, **f**, **g**, **h**, and **4–6**.

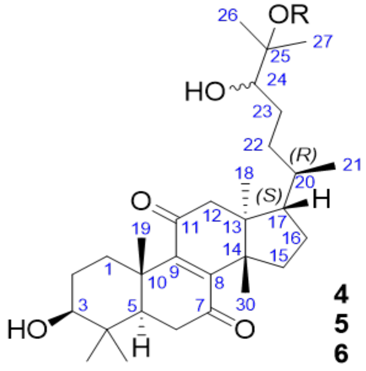

4 R = H
5 R = H
6 R = CH_2CH_3

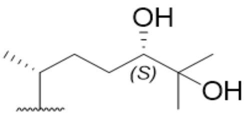

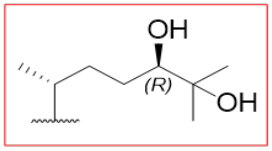

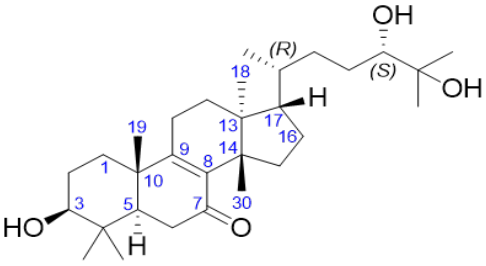

neritriterpenol A (**e**)
Phytochemistry **2022**, *199*, 113199

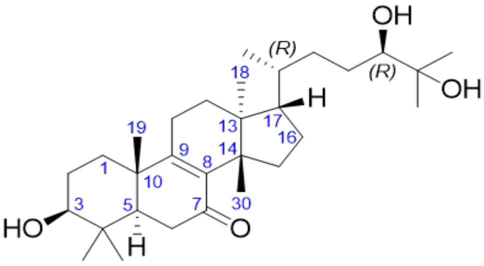

neritriterpenol B (**f**)
Phytochemistry **2022**, *199*, 113199

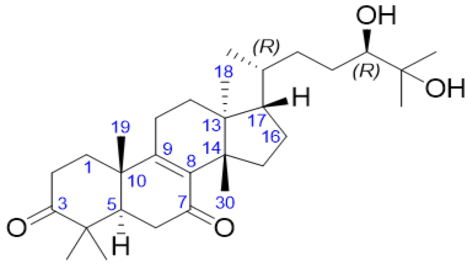

neritriterpenol G (**g**)
Phytochemistry **2022**, *199*, 113199

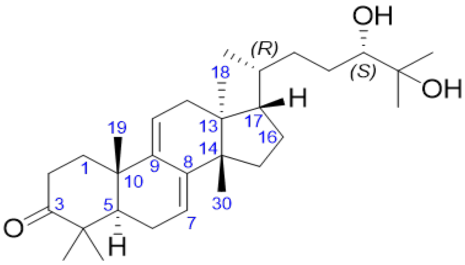

ganodermanondiol (**h**)
J. Nat. Prod. **1988**, *51*, 54¡V59

δ_C (ppm)	C-20	C-21	C-22	C-23	C-24	C-25	C-26	C-27
**e**	36.1	19.1	32.6	28.3	79.5	73.3	23.2	26.6
**f**	35.4	18.8	32.2	27.9	78.6	73.3	23.2	26.6
**g**	35.4	18.8	32.1	27.9	78.6	73.3	23.2	26.7
**h**	36.5	18.6	31.5	28.7	79.6	73.3	23.2	26.6
**3**	36.5	18.7	32.2	28.6	79.3	73.2	23.4	26.5
**4**	35.6	18.4	31.8	28.2	78.4	73.1	23.4	26.6
**5**	36.5	18.7	32.3	28.8	79.3	73.2	23.3	26.6
**6**	35.4	18.4	31.6	28.2	78.2	73.2	23.3	26.6

Table S3. Crystal data and structure refinement for neritriterpenol J (**3**).

Identification code	d21252	
Empirical formula	$C_{30} H_{52}O_5$	
Formula weight	492.72	
Temperature	200(2) K	
Wavelength	0.71073 Å	
Crystal system	Monoclinic	
Space group	P 21	
Unit cell dimensions	a = 12.0641(5) Å	α= 90°.
	b = 7.0406(2) Å	β= 94.7800(10)°.
	c = 17.1863(7) Å	γ = 90°.
Volume	1454.70(9) $Å^3$	
Z	2	
Density (calculated)	1.125 Mg/m^3	
Absorption coefficient	0.074 mm^{-1}	
F(000)	544	
Crystal size	0.56 x 0.10 x 0.02 mm^3	
Theta range for data collection	2.38 to 25.07°.	
Index ranges	-14<=h<=14, -8<=k<=8, -20<=l<=20	
Reflections collected	27930	
Independent reflections	5130 [R(int) = 0.0678]	
Completeness to theta = 25.07°	99.4 %	
Absorption correction	multi-scan	
Max. and min. transmission	0.9985 and 0.9596	
Refinement method	Full-matrix least-squares on F^2	
Data / restraints / parameters	5130 / 1 / 326	
Goodness-of-fit on F^2	1.059	
Final R indices [I>2sigma(I)]	R1 = 0.0392, wR2 = 0.0878	
R indices (all data)	R1 = 0.0547, wR2 = 0.0977	
Absolute structure parameter	0.9(10)	
Largest diff. peak and hole	0.137 and -0.147 e.$Å^{-3}$	

Table S4. Crystal data and structure refinement for neritriterpenol L (**5**).

Identification code	d21103	
Empirical formula	$C_{30}H_{50}O_6$	
Formula weight	506.70	
Temperature	200(2) K	
Wavelength	0.71073 Å	
Crystal system	Orthorhombic	
Space group	P 21 21 21	
Unit cell dimensions	a = 6.9896(3) Å	α= 90°.
	b = 12.3010(6) Å	β= 90°.
	c = 33.8198(17) Å	γ = 90°.
Volume	2907.8(2) Å^3	
Z	4	
Density (calculated)	1.157 Mg/m^3	
Absorption coefficient	0.079 mm^{-1}	
F(000)	1112	
Crystal size	0.60 x 0.08 x 0.03 mm^3	
Theta range for data collection	2.41 to 25.08°.	
Index ranges	-8<=h<=7, -13<=k<=14, -40<=l<=40	
Reflections collected	15236	
Independent reflections	5135 [R(int) = 0.0676]	
Completeness to theta = 25.08°	99.4 %	
Absorption correction	multi-scan	
Max. and min. transmission	0.9976 and 0.9544	
Refinement method	Full-matrix least-squares on F^2	
Data / restraints / parameters	5135 / 0 / 335	
Goodness-of-fit on F^2	1.054	
Final R indices [I>2sigma(I)]	R1 = 0.0513, wR2 = 0.0963	
R indices (all data)	R1 = 0.0989, wR2 = 0.1173	
Absolute structure parameter	-1.0(14)	
Largest diff. peak and hole	0.155 and -0.191 e.Å^{-3}	

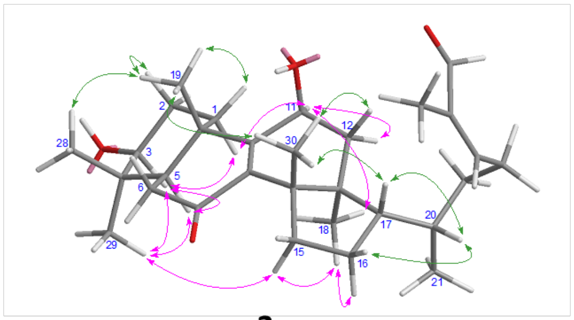

2

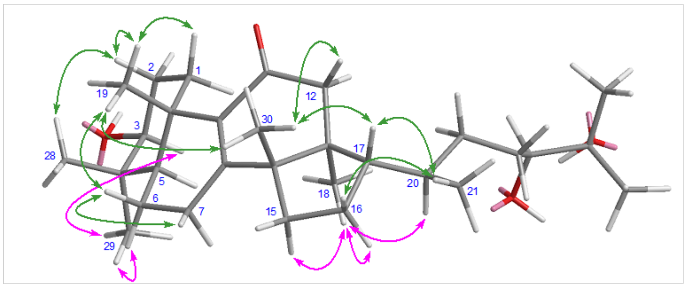

3

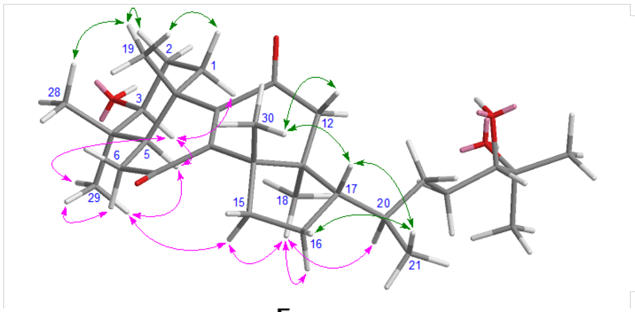

5

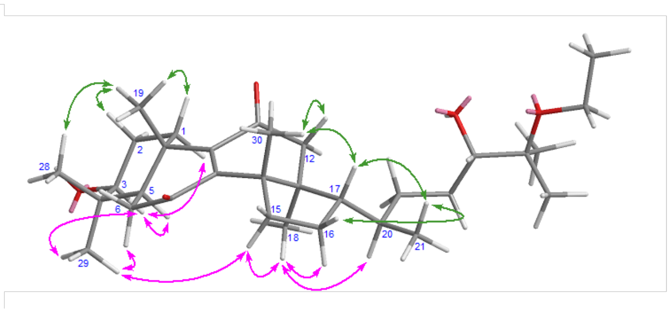

7

Figure S1. Selected NOESY correlations of compounds **2**, **3**, **5**, and **7**.

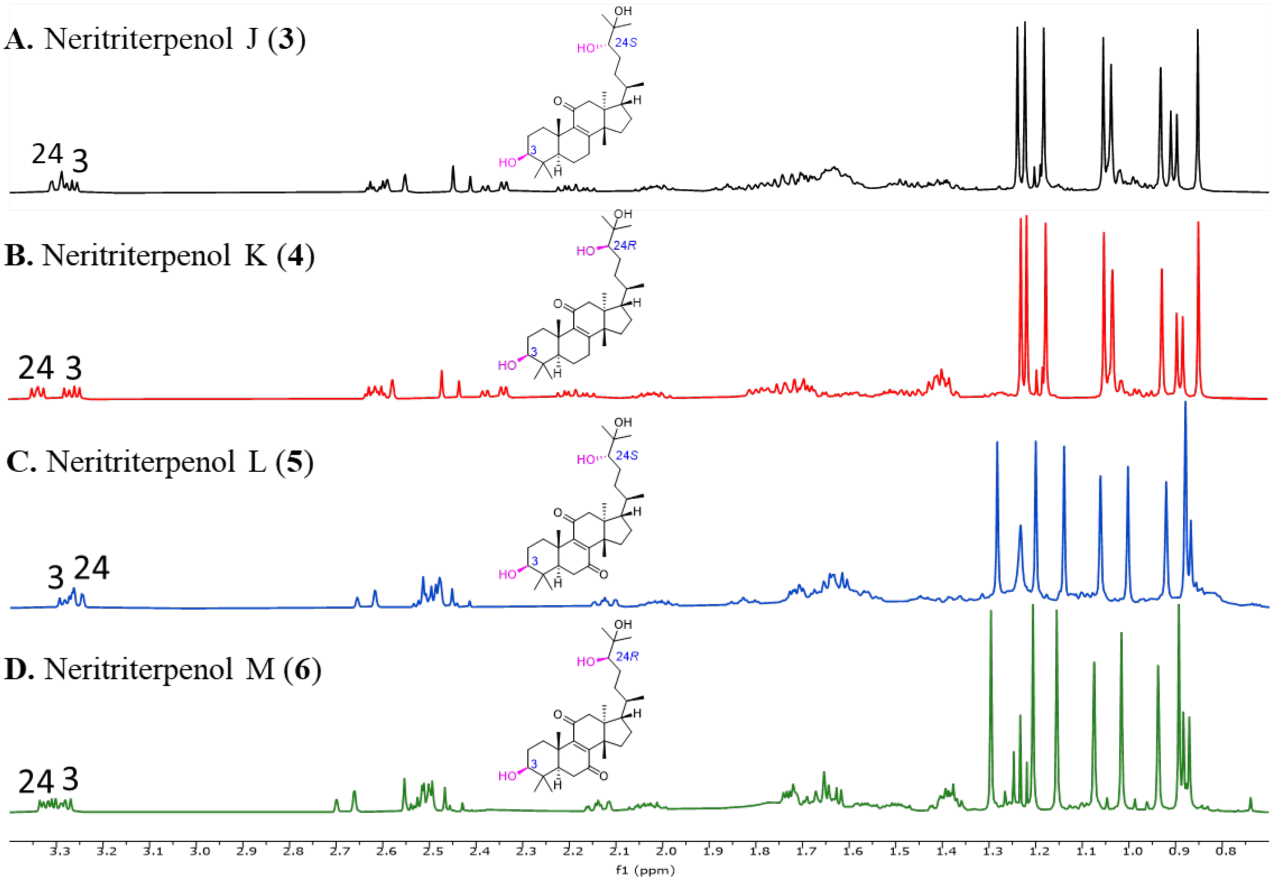

Figure S2. ^{1}H-NMR alignment of compounds **3**−**6** (**A**−**D**). The assignments of H-3 and H-24 in each compound were assigned.

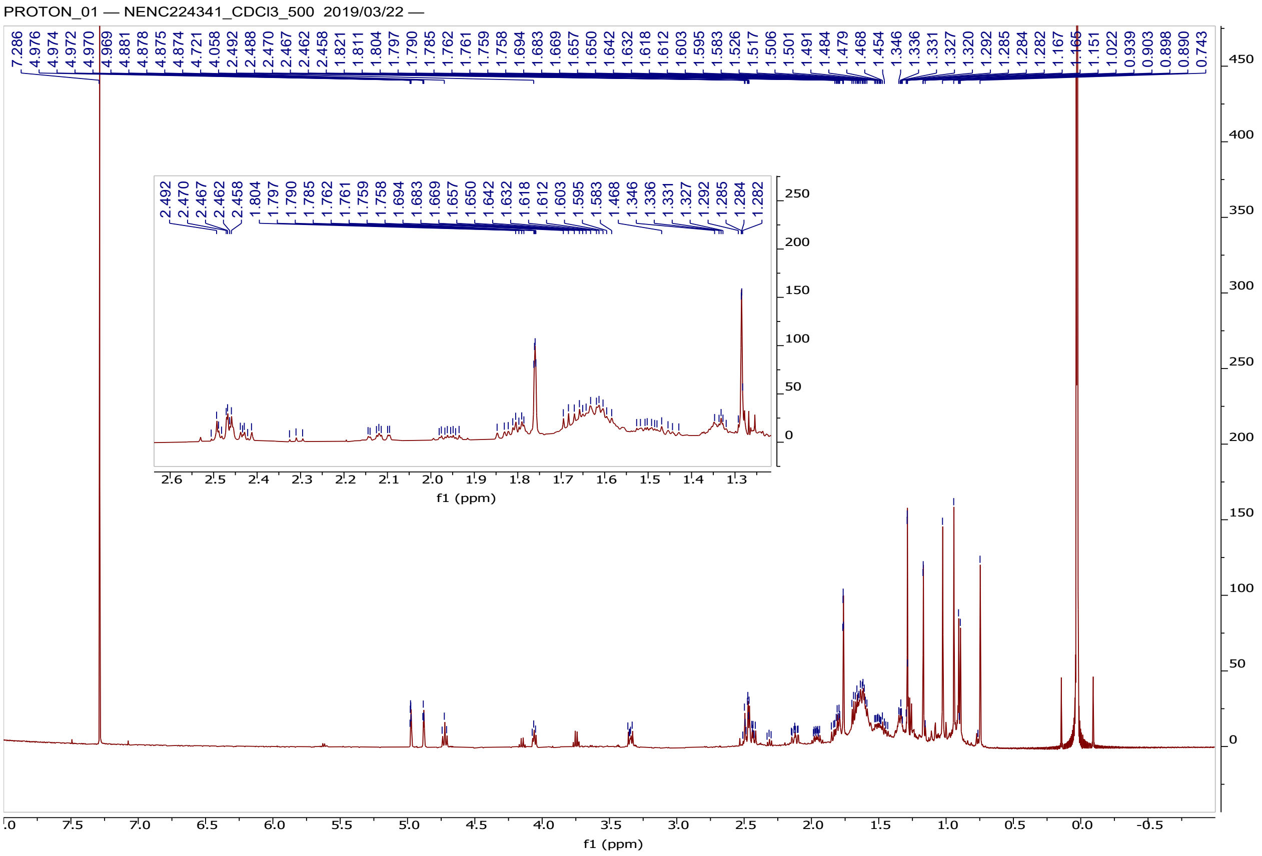

Figure S3. ^{1}H-NMR spectrum of neritriterpenol H (**1**) in $CDCl_3$ (500 MHz)

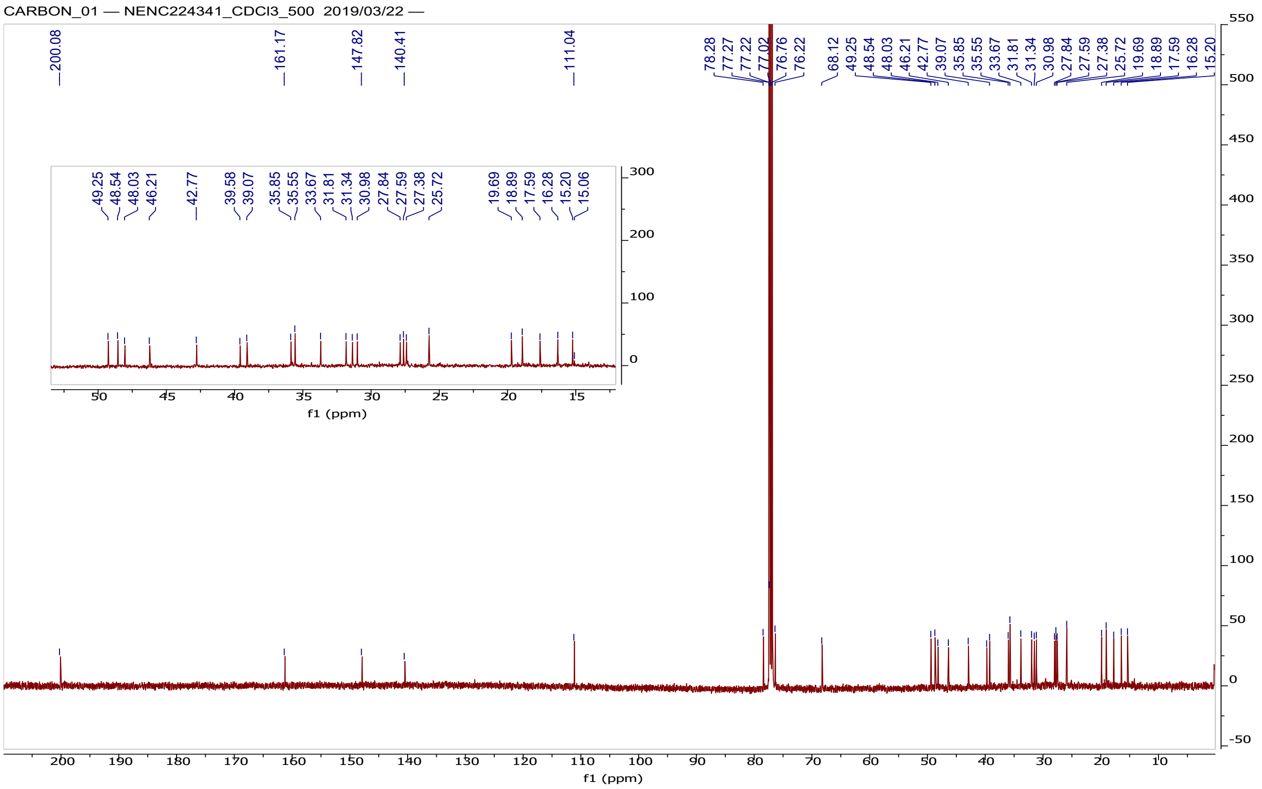

Figure S4. ^{13}C-NMR spectrum of neritriterpenol H (**1**) in $CDCl_3$ (125 MHz)

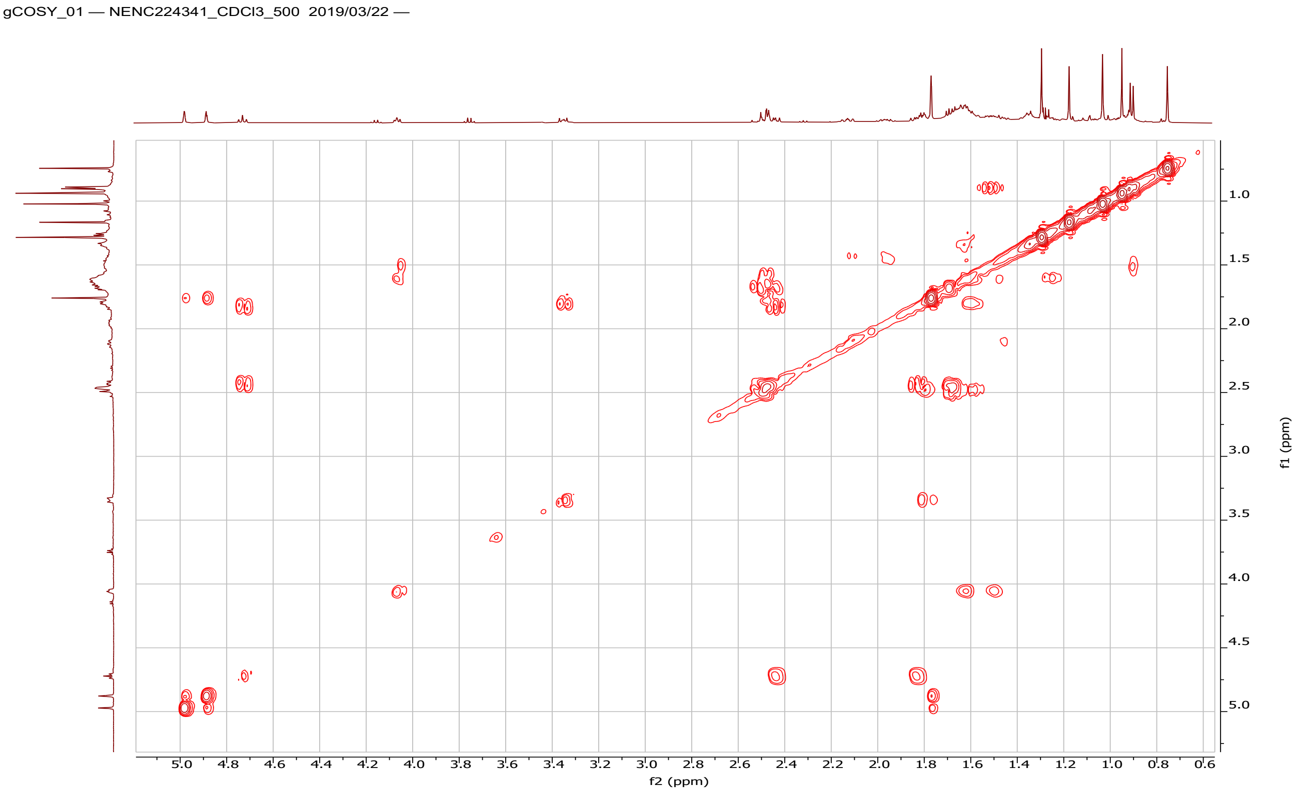

Figure S5. ¹H-¹H COSY spectrum of neritriterpenol H (**1**) in $CDCl_3$

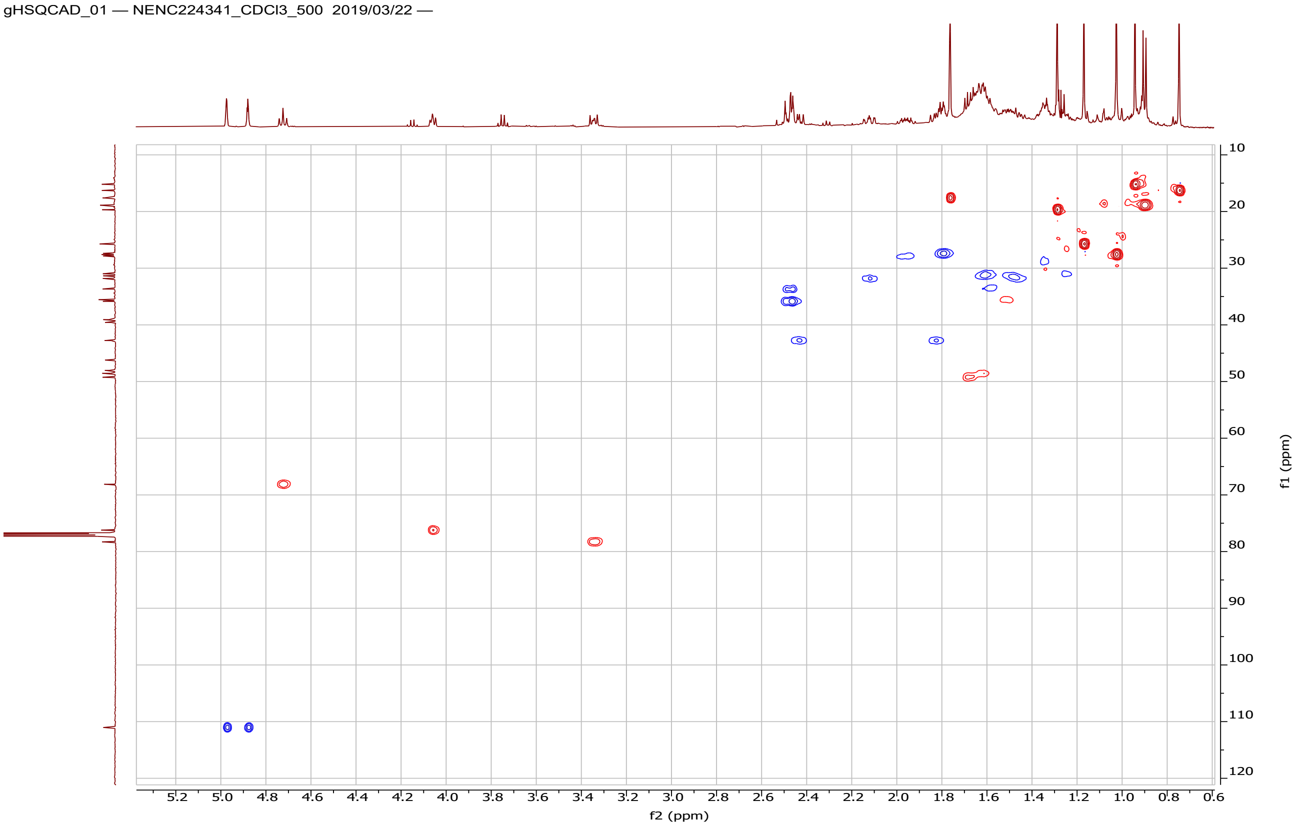

Figure S6. HSQC spectrum of neritriterpenol H (**1**) in $CDCl_3$

gHMBCAD_01 — NENC224341_CDCl3_500 2019/03/22 —

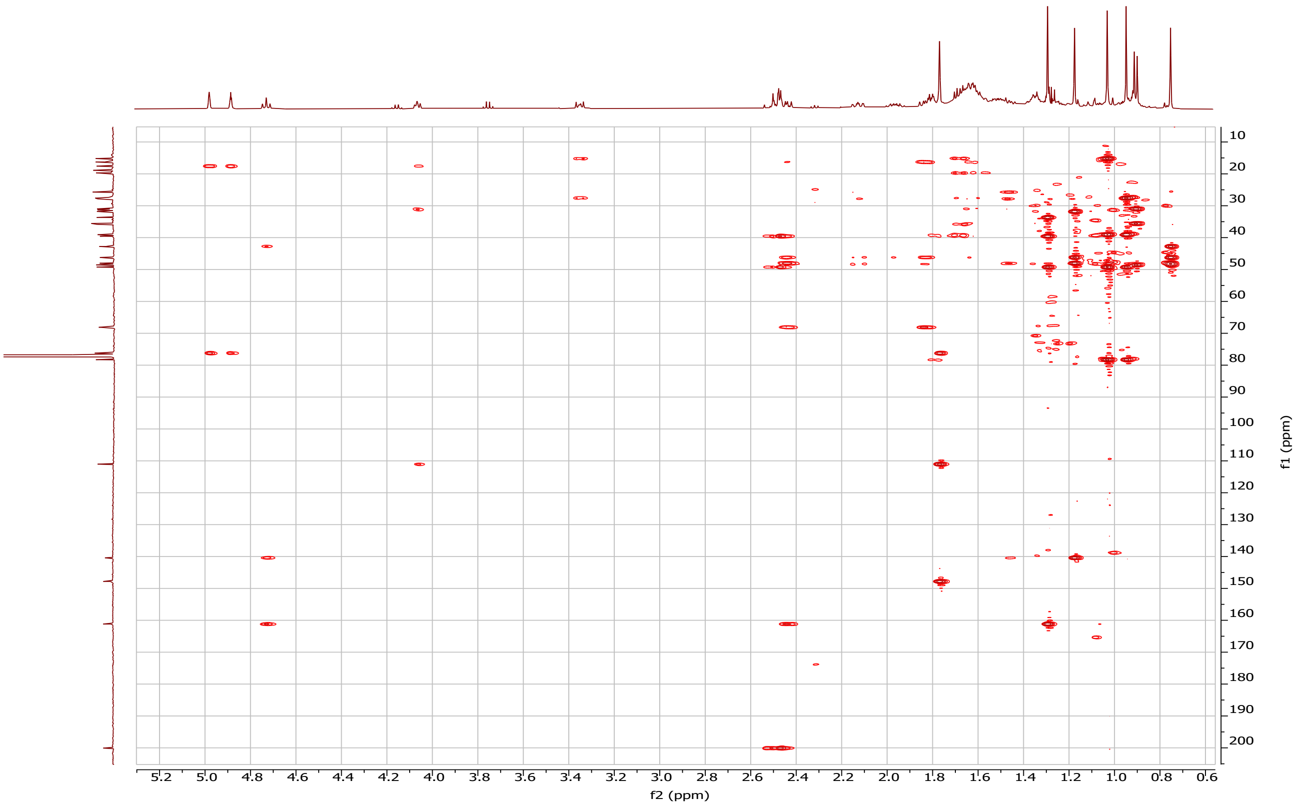

Figure S7. HMBC spectrum of neritriterpenol H (**1**) in $CDCl_3$

NOESY_01 — NENC224341_CDCl3_500 2019/03/27 —

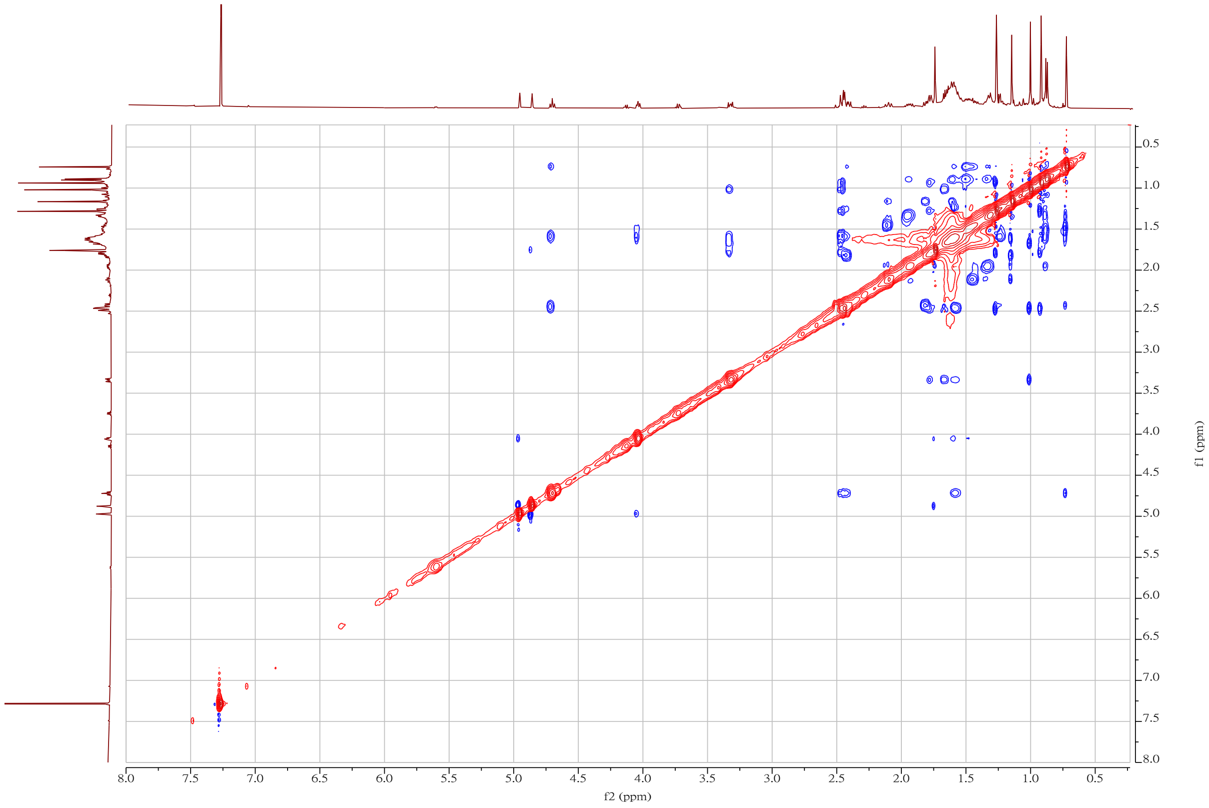

Figure S8. NOESY spectrum of neritriterpenol H (**1**) in $CDCl_3$

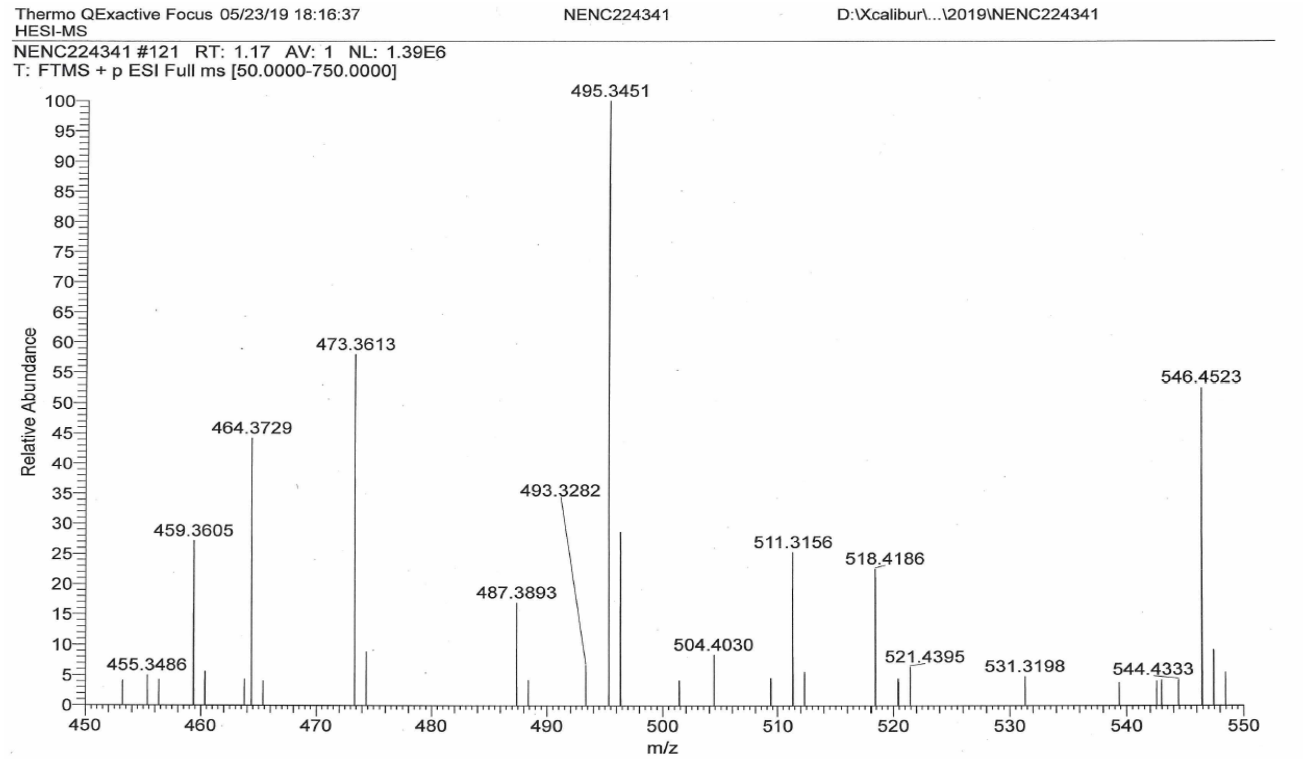

Figure S9. HRESIMS spectrum of neritriterpenol H (**1**)

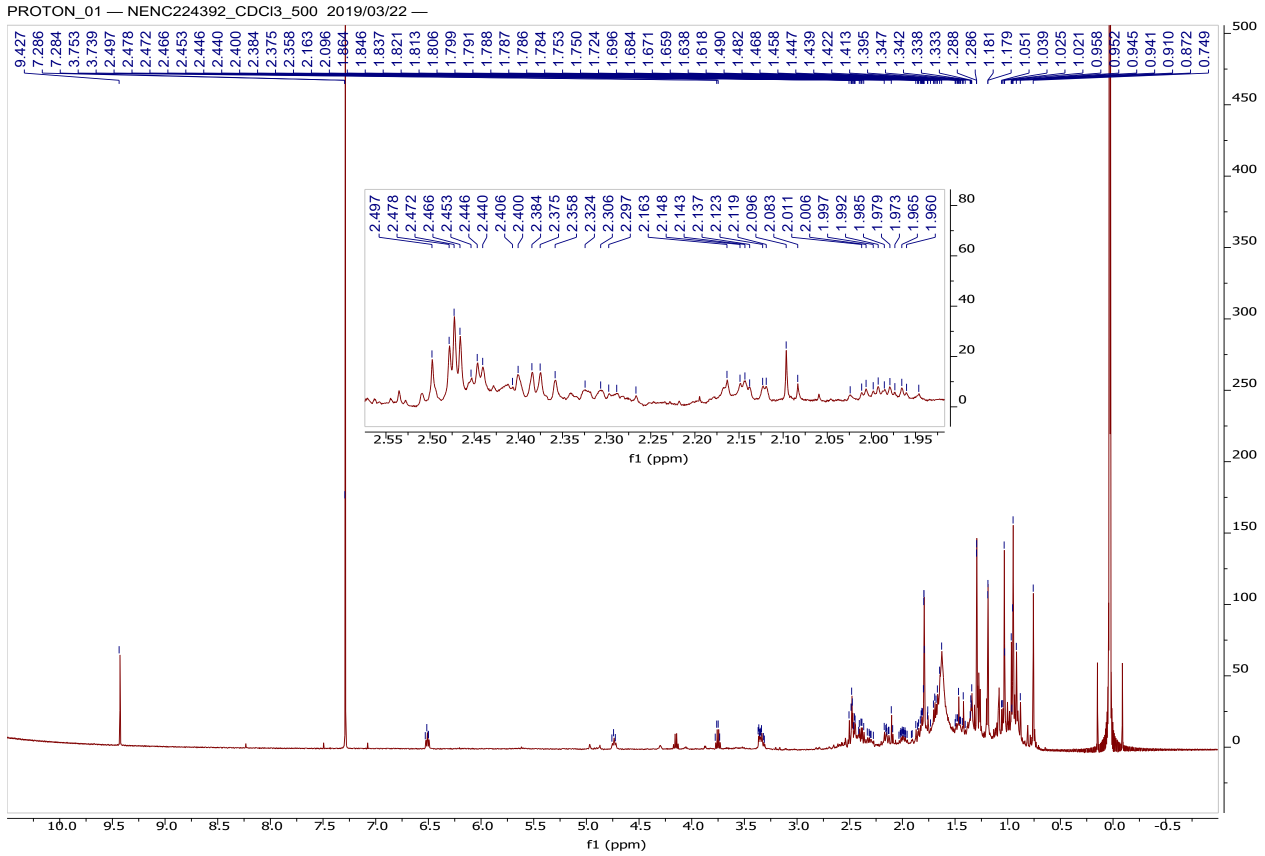

Figure S10. ^{1}H-NMR spectrum of neritriterpenol I (**2**) in $CDCl_3$ (500 MHz)

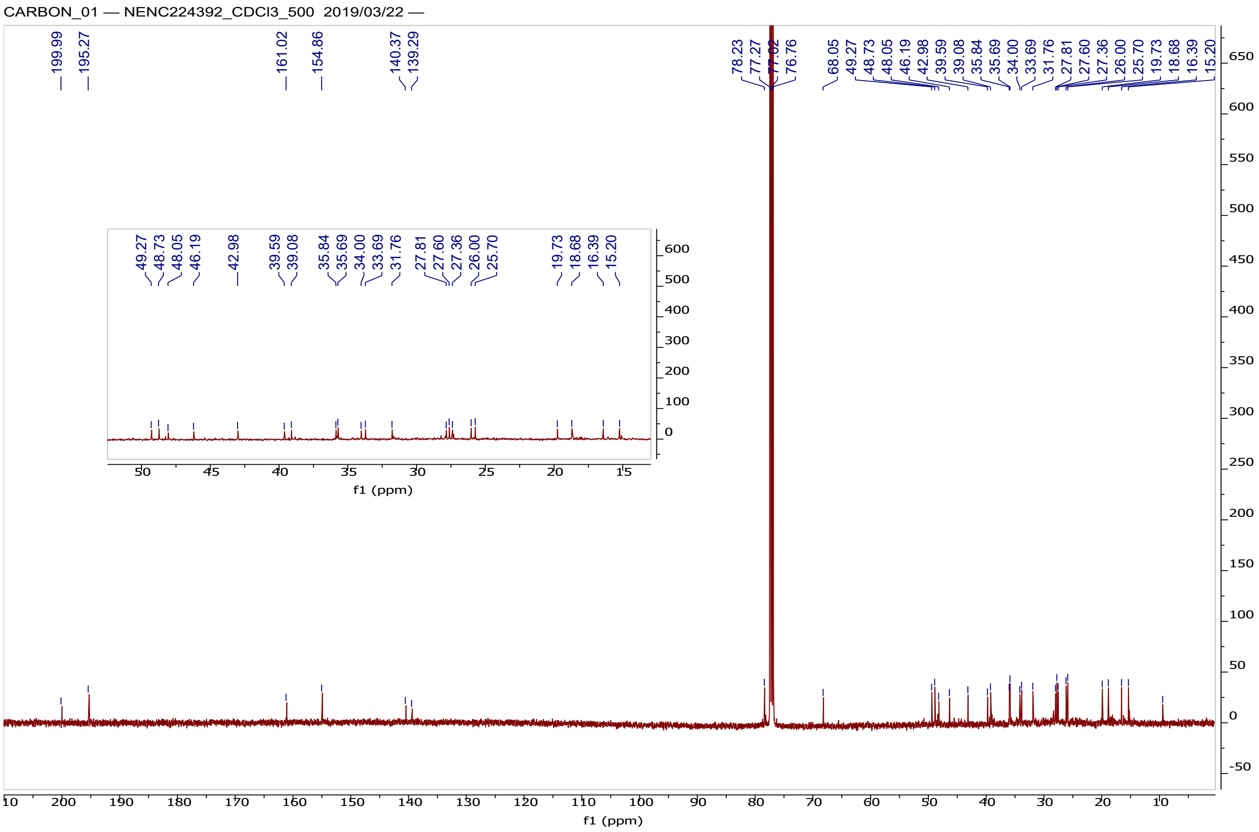

Figure S11. ^{13}C-NMR spectrum of neritriterpenol I (**2**) in $CDCl_3$ (125 MHz)

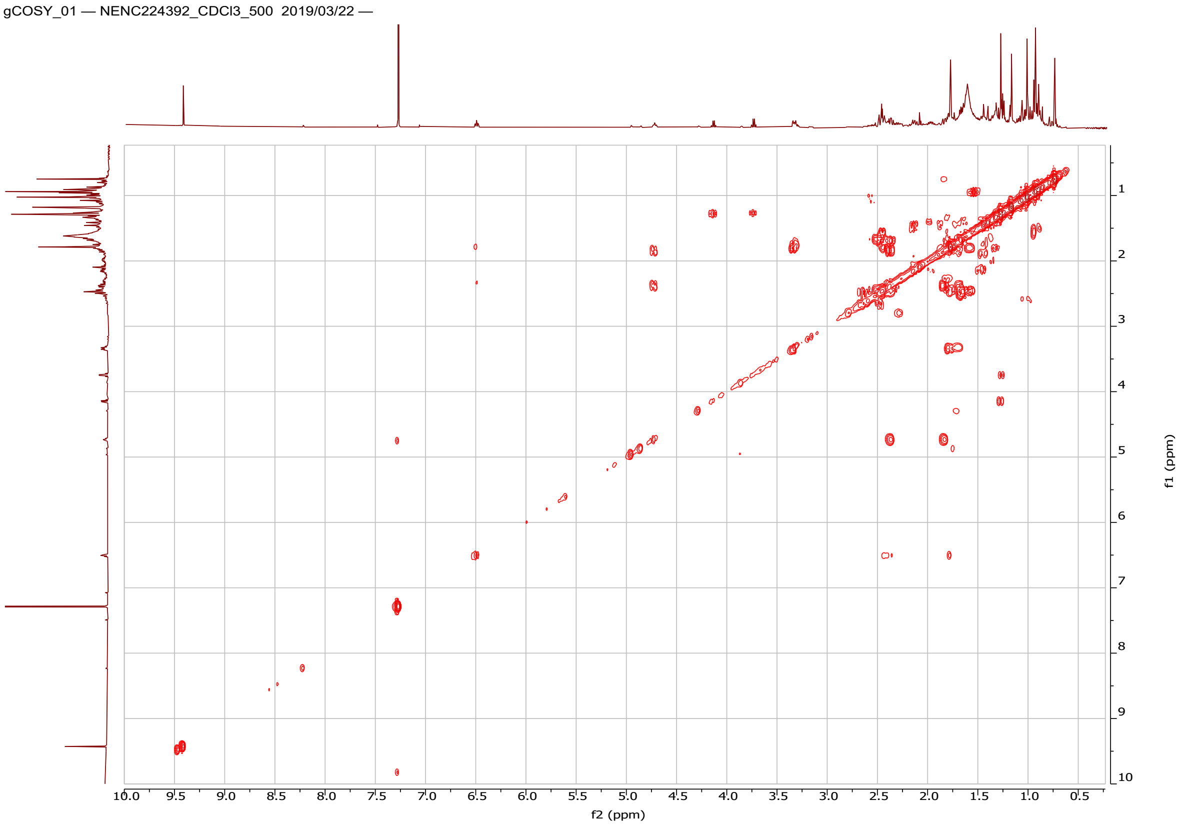

Figure S12. ^{1}H-^{1}H COSY spectrum of neritriterpenol I (**2**) in $CDCl_3$

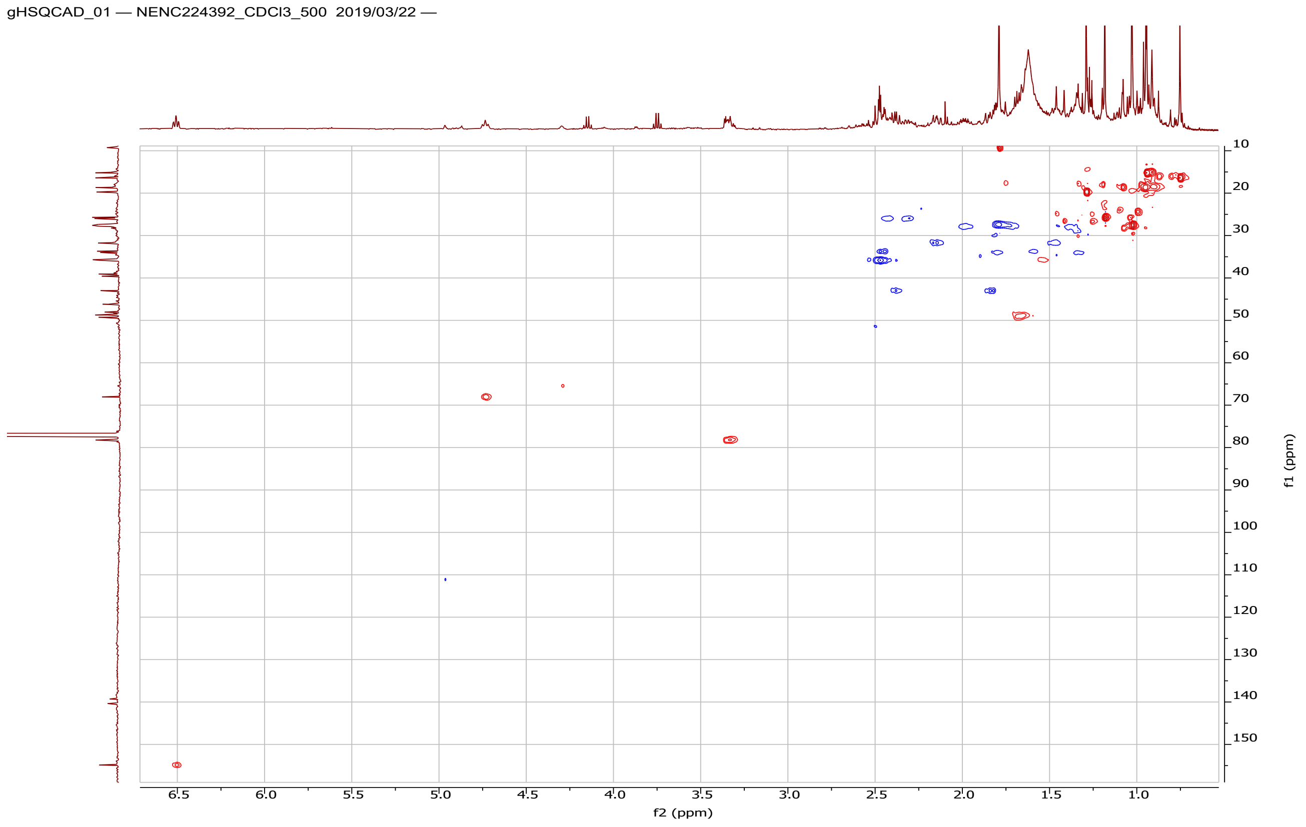

Figure S13. HSQC spectrum of neritriterpenol I (**2**) in $CDCl_3$

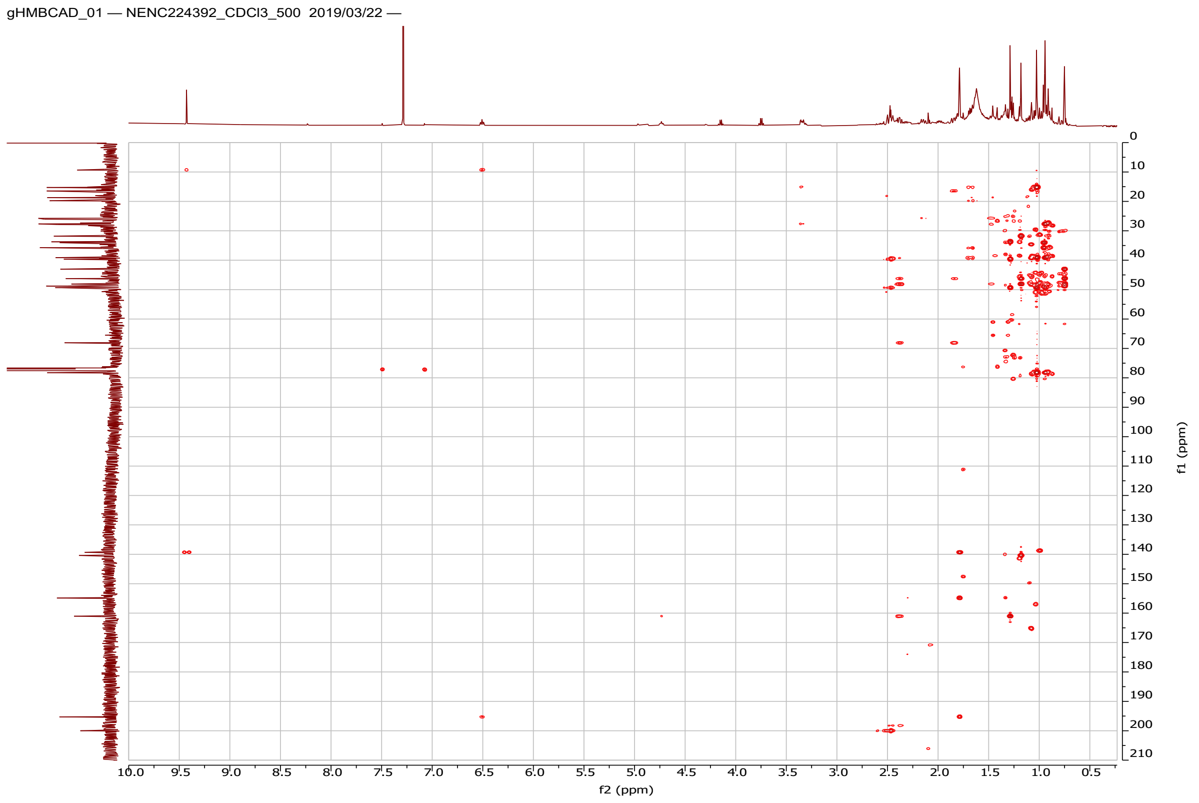

Figure S14. HMBC spectrum of neritriterpenol I (**2**) in $CDCl_3$

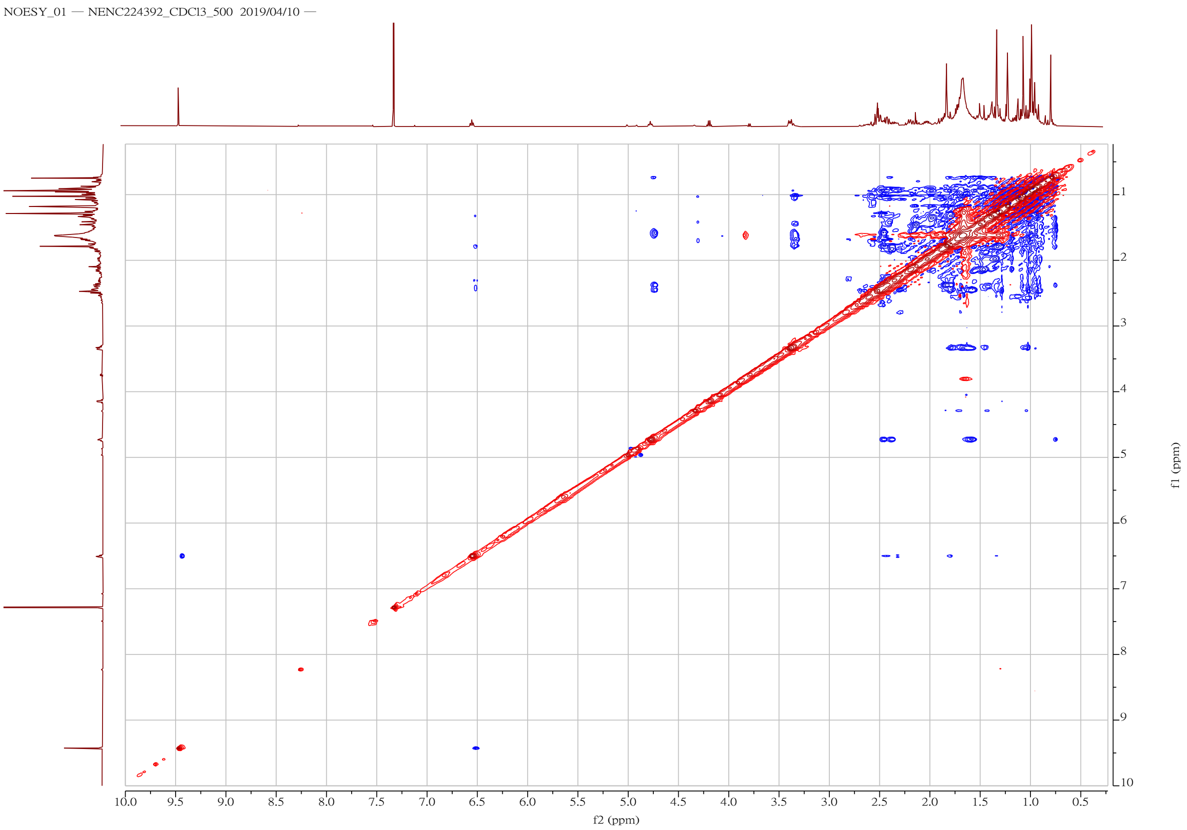

Figure S15. NOESY spectrum of neritriterpenol I (**2**) in $CDCl_3$

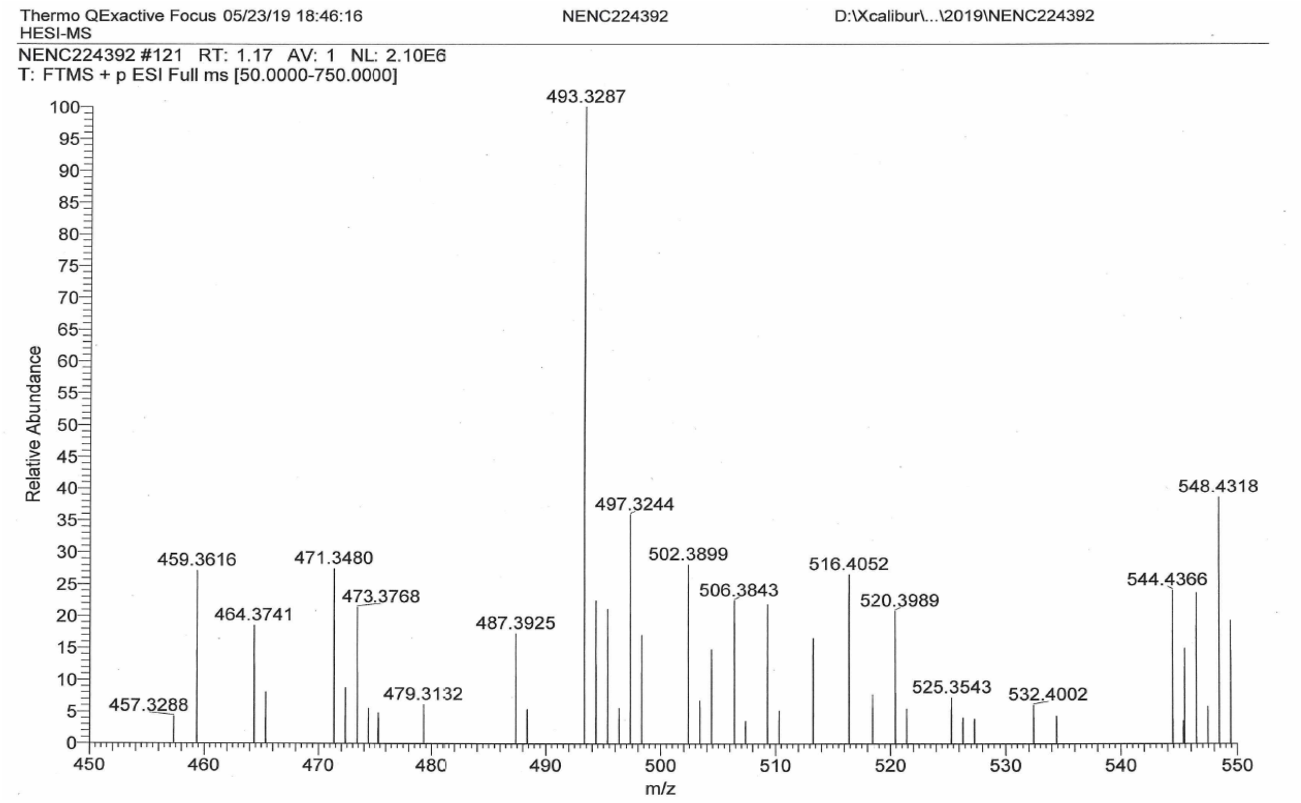

Figure S16. HRESIMS spectrum of neritriterpenol I (**2**)

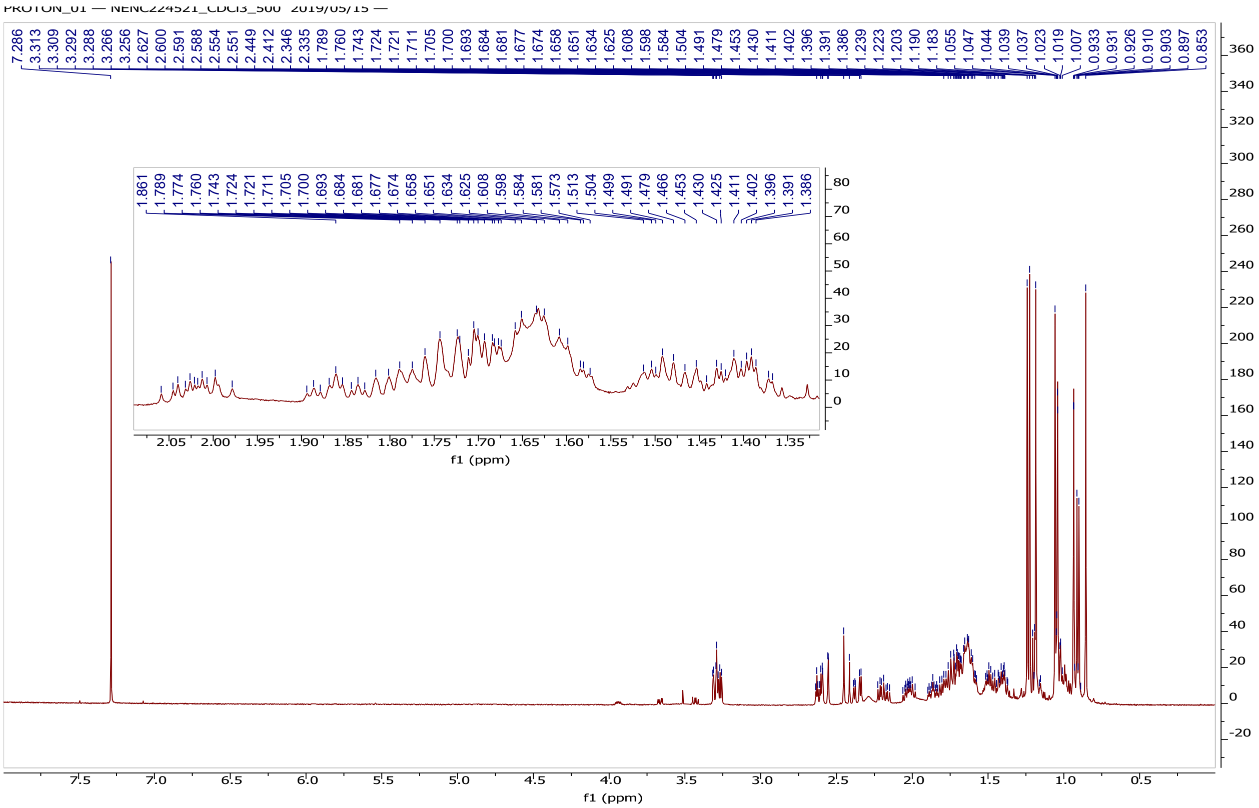

Figure S17. ^{1}H-NMR spectrum of neritriterpenol J (**3**) in $CDCl_3$ (500 MHz)

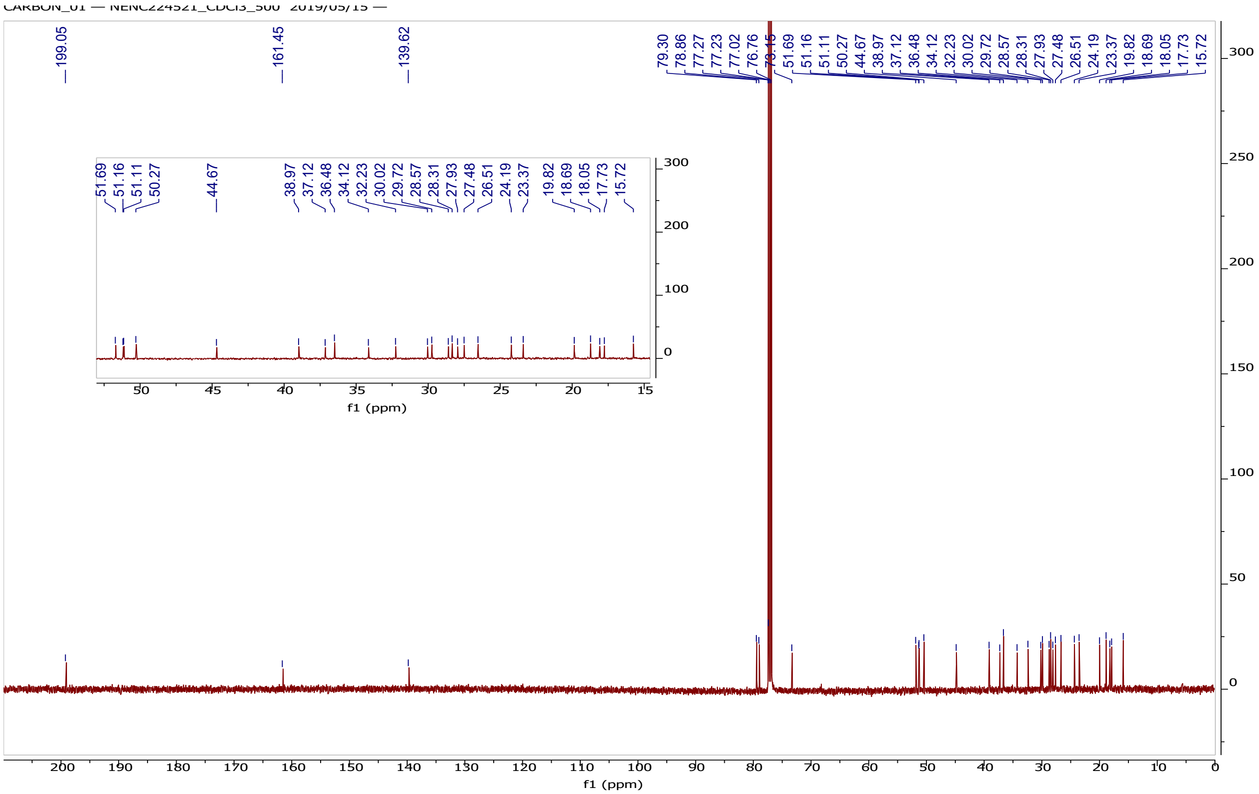

Figure S18. ^{13}C-NMR spectrum of neritriterpenol J (**3**) in $CDCl_3$ (125 MHz)

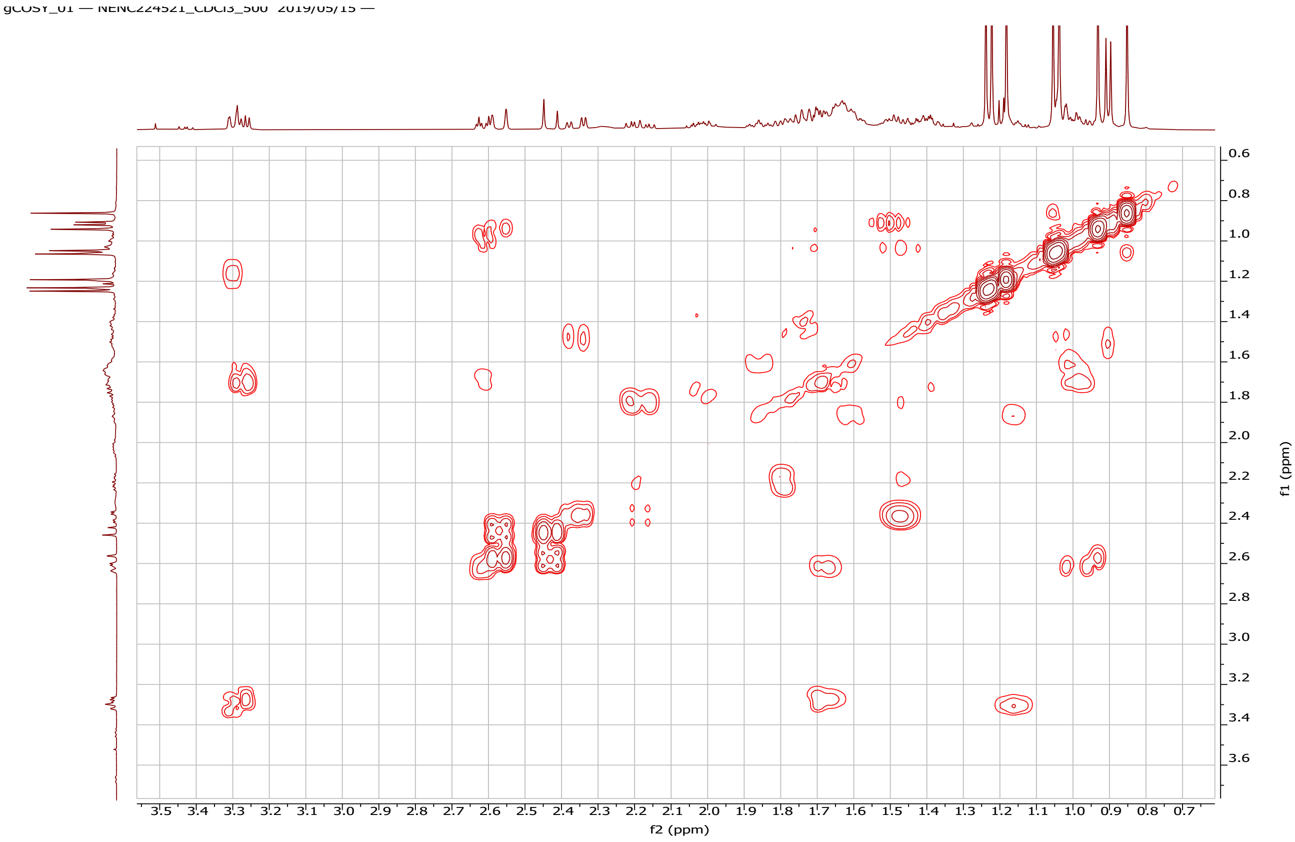

Figure S19. ^{1}H-^{1}H COSY spectrum of neritriterpenol J (**3**) in $CDCl_3$

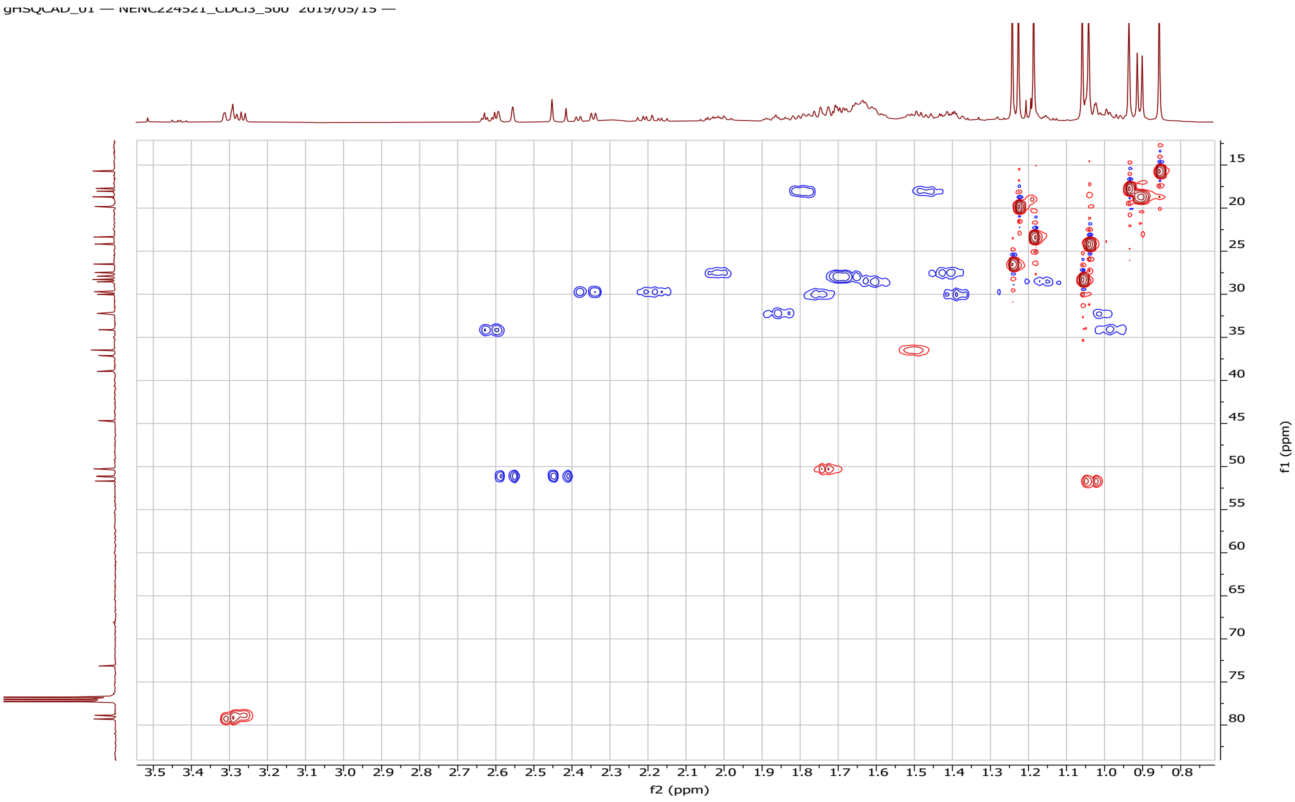

Figure S20. HSQC spectrum of neritriterpenol J (**3**) in $CDCl_3$

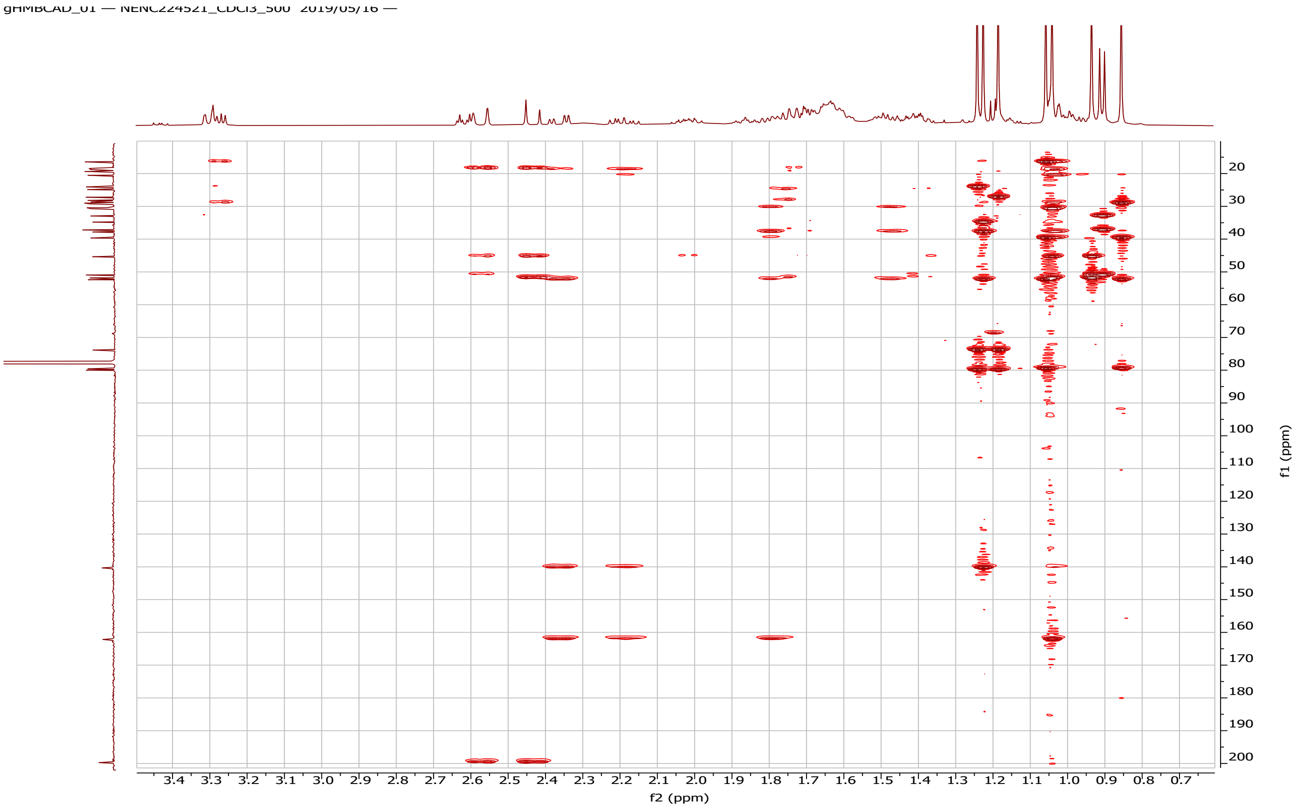

Figure S21. HMBC spectrum of neritriterpenol J (**3**) in $CDCl_3$

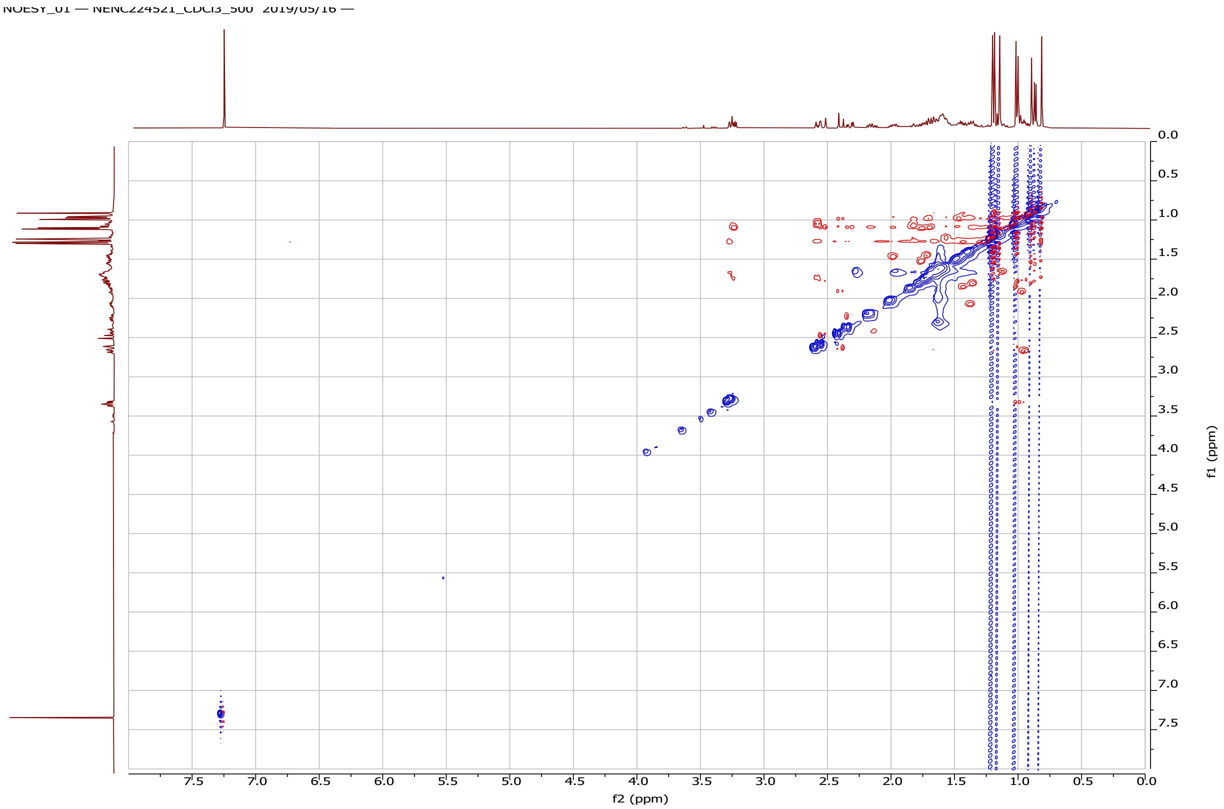

Figure S22. NOESY spectrum of neritriterpenol J (**3**) in $CDCl_3$

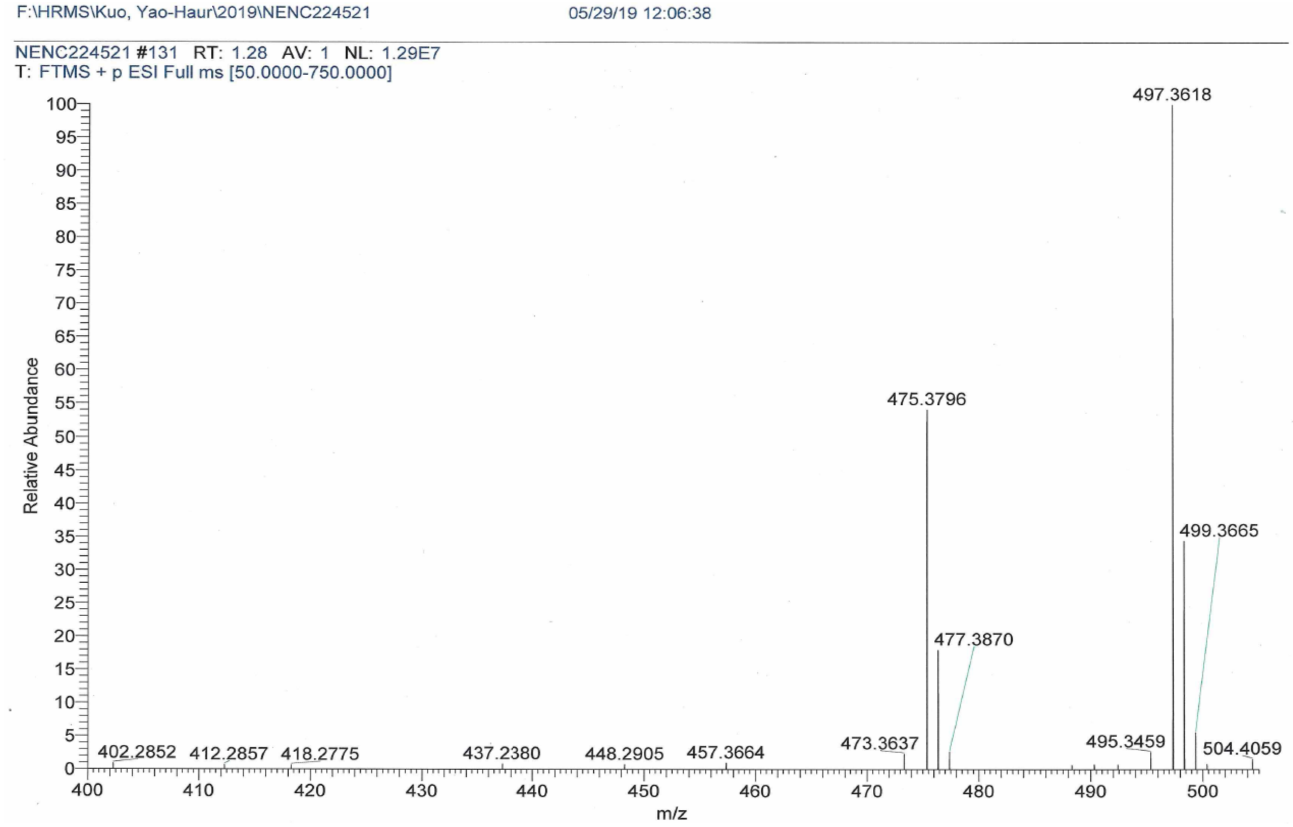

Figure S23. HRESIMS spectrum of neritriterpenol J (**3**)

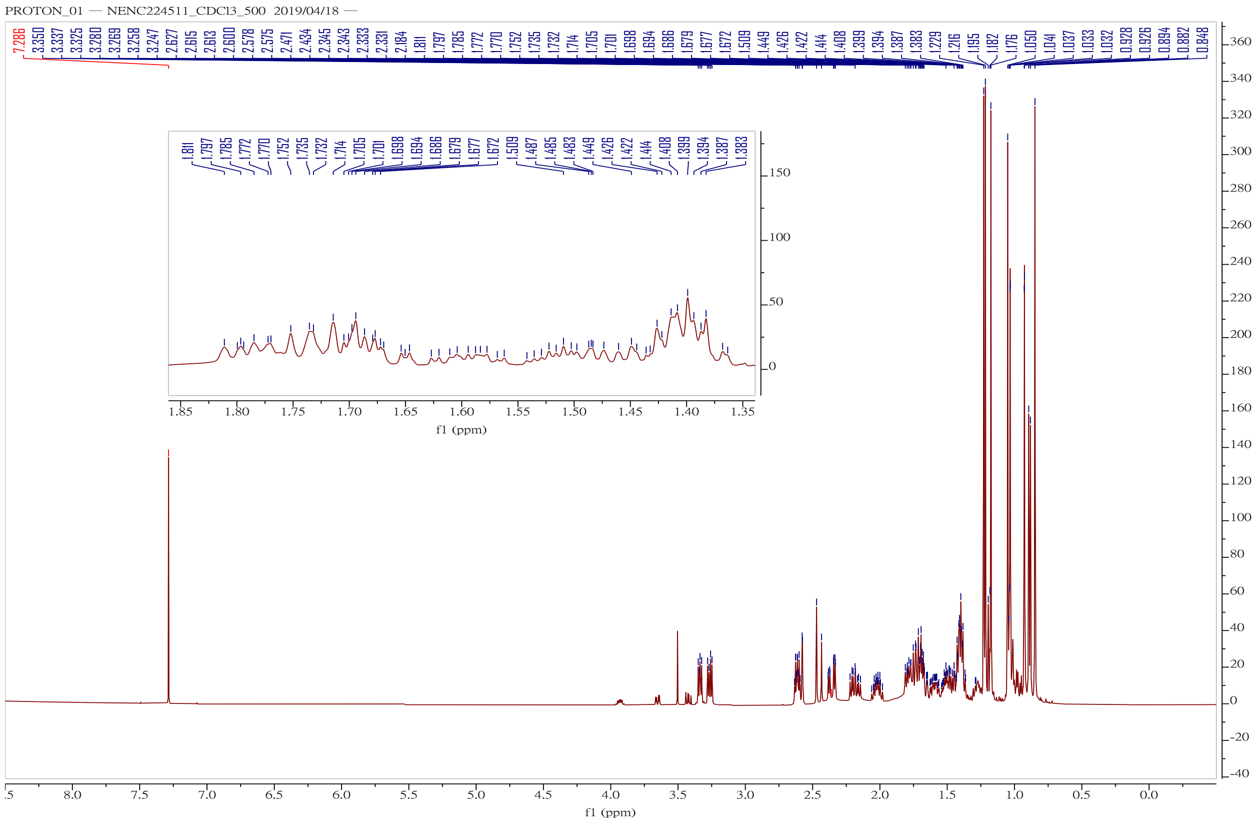

Figure S24. ^{1}H NMR spectrum of neritriterpenol K (**4**) in $CDCl_3$ (500 MHz)

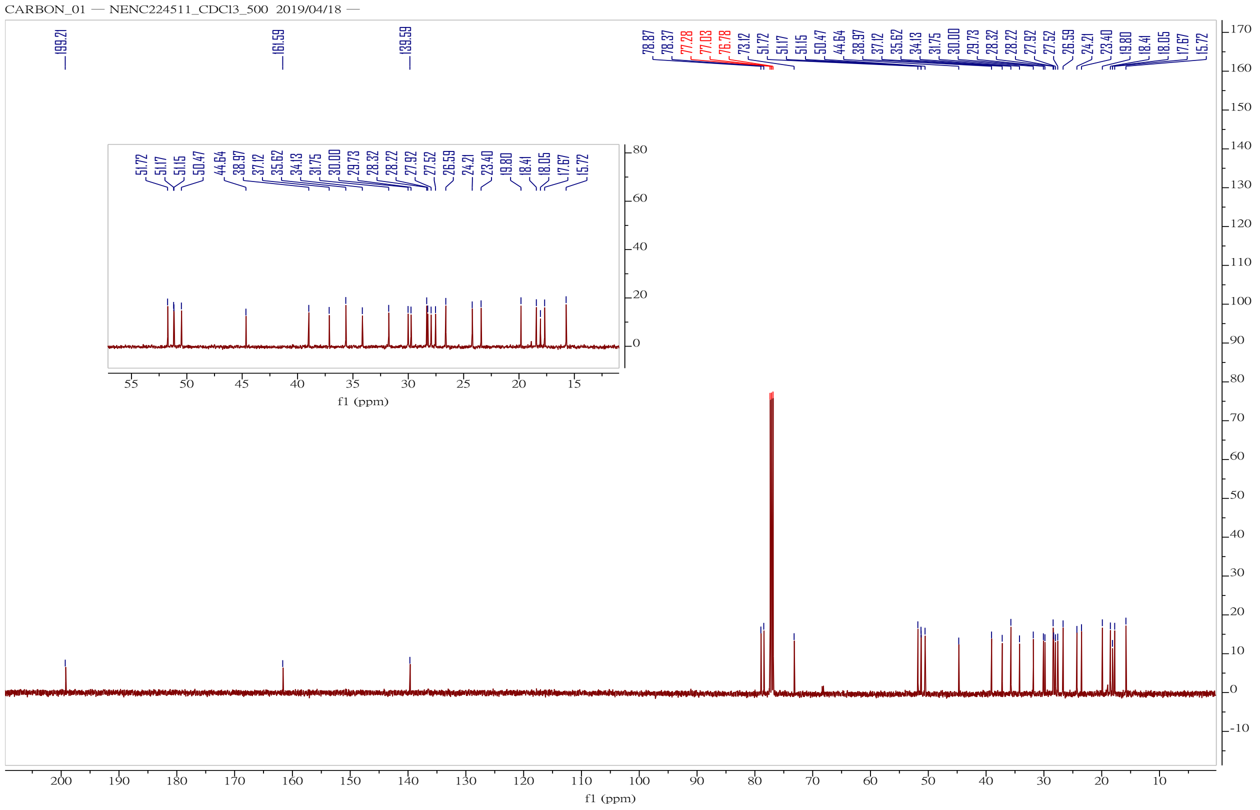

Figure S25. ^{13}C NMR spectrum of neritriterpenol K (**4**) in $CDCl_3$ (125 MHz)

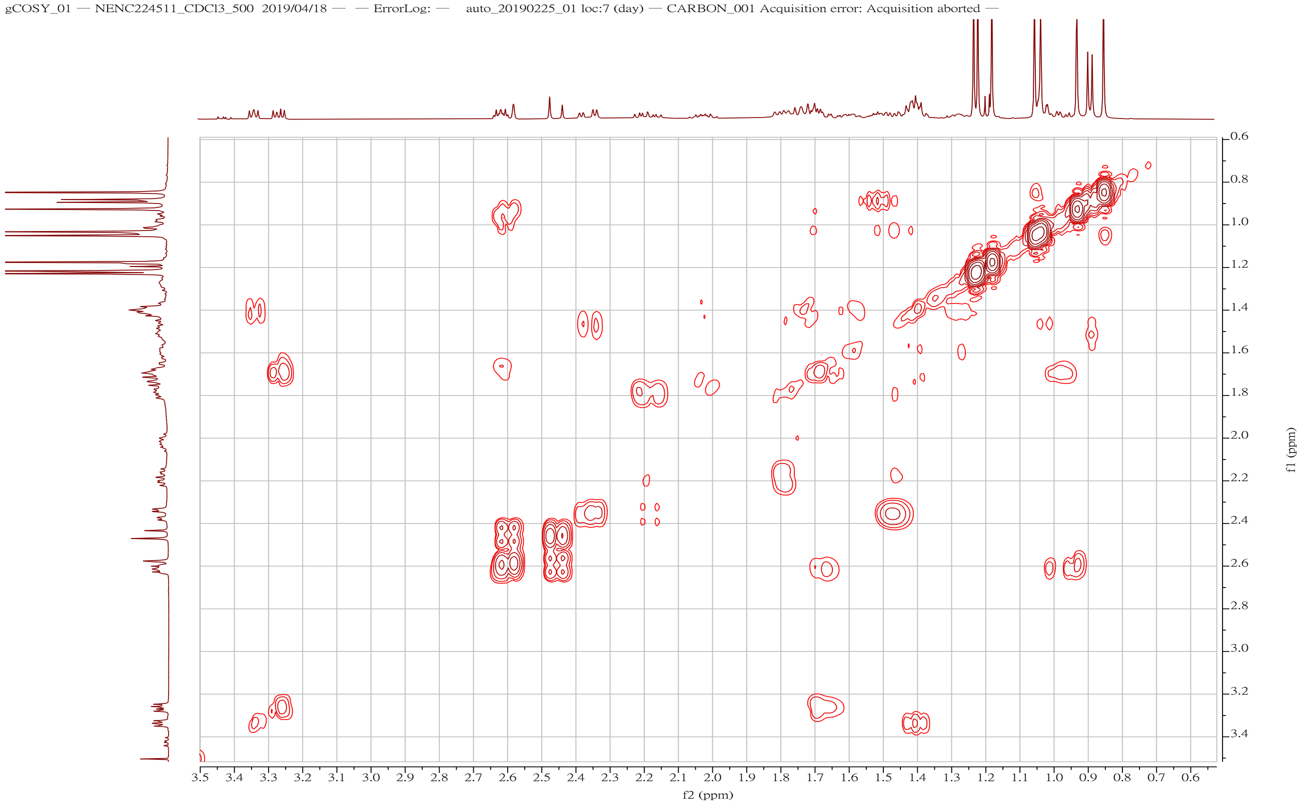

Figure S26. ^{1}H-^{1}H COSY spectrum of neritriterpenol K (**4**) in $CDCl_3$

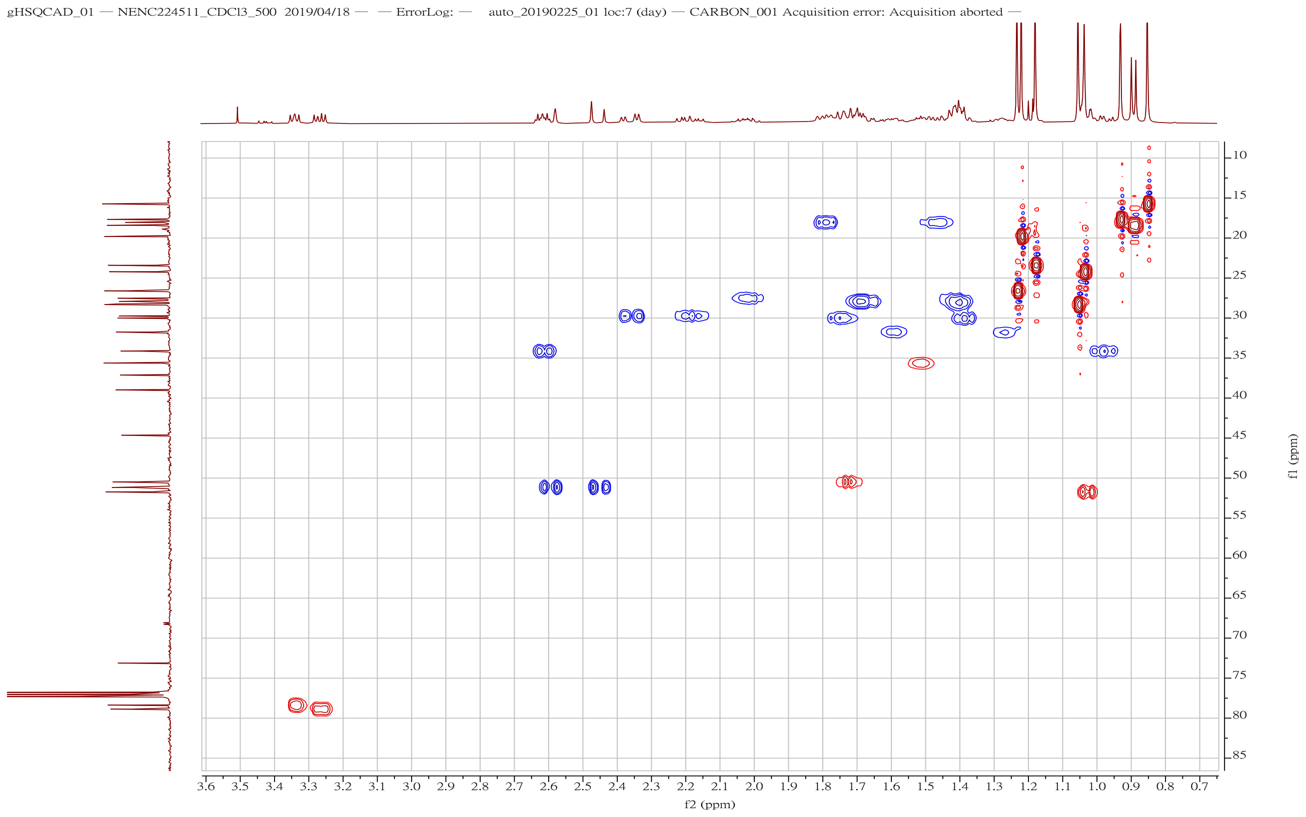

Figure S27. HSQC spectrum of neritriterpenol K (**4**) in $CDCl_3$

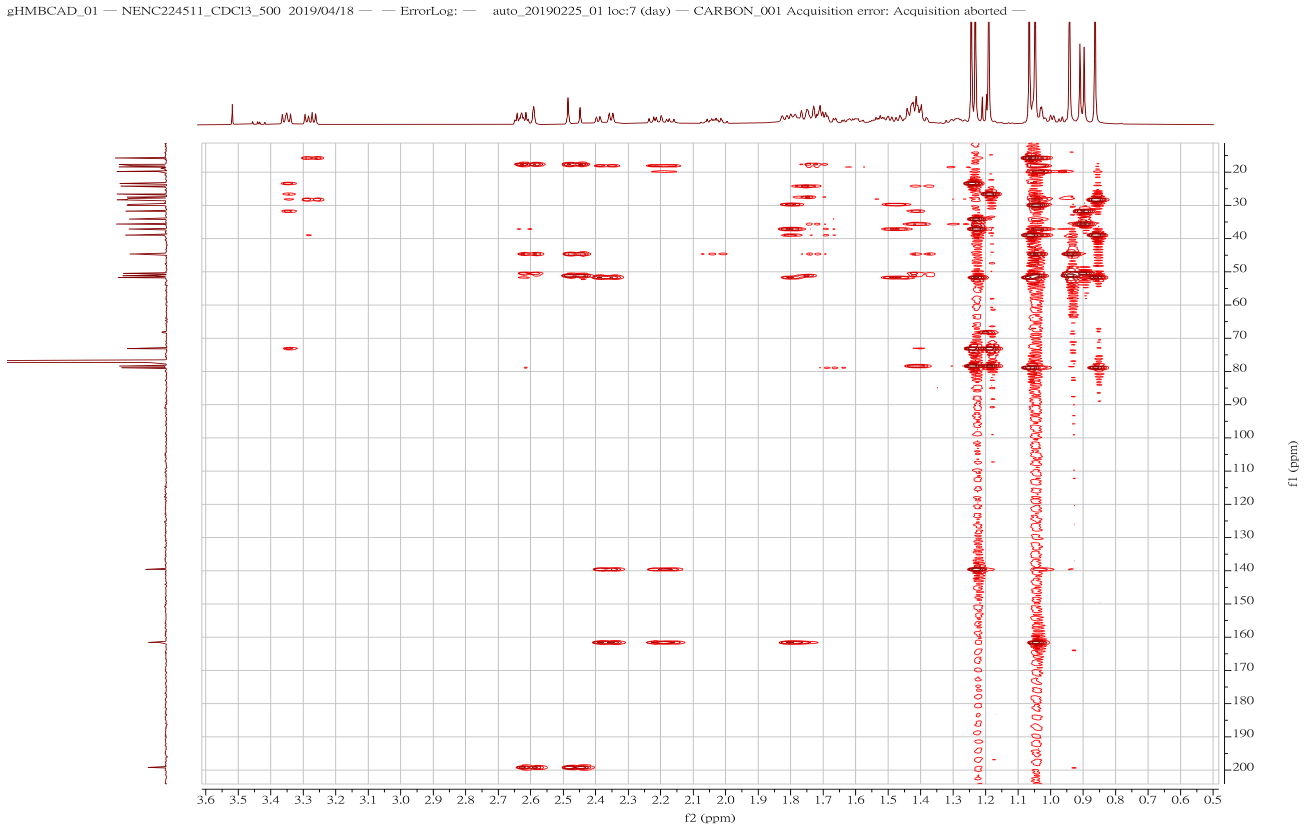

Figure S28. HMBC spectrum of neritriterpenol K (**4**) in $CDCl_3$

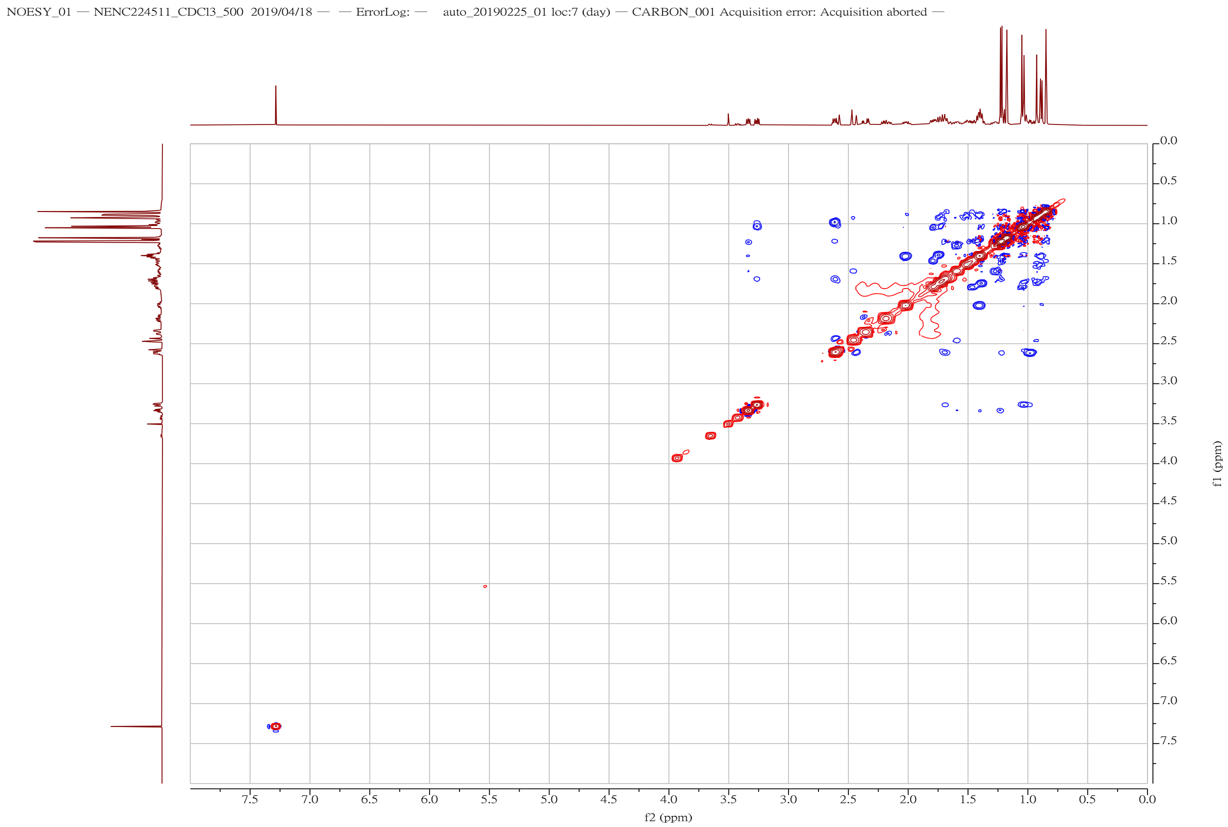

Figure S29. NOESY spectrum of neritriterpenol K (**4**) in $CDCl_3$

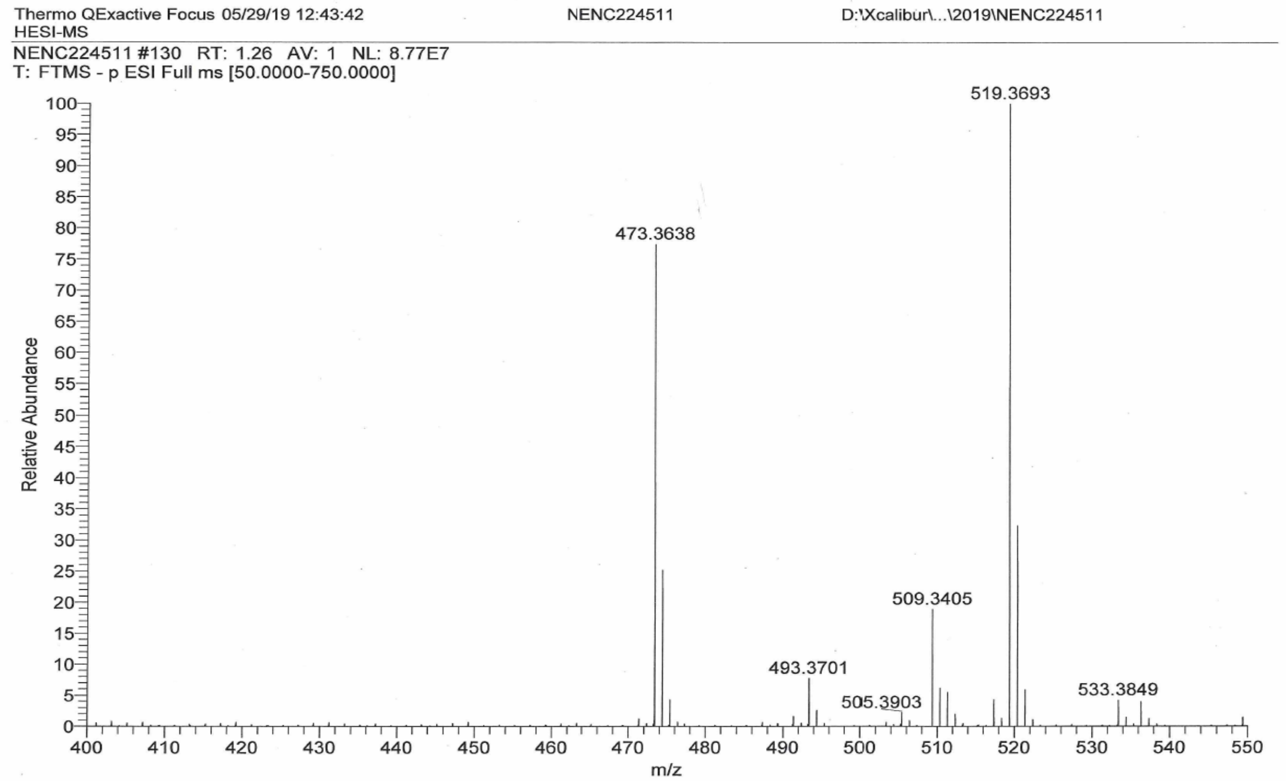

Figure S30. HRESIMS spectrum of neritriterpenol K (**4**)

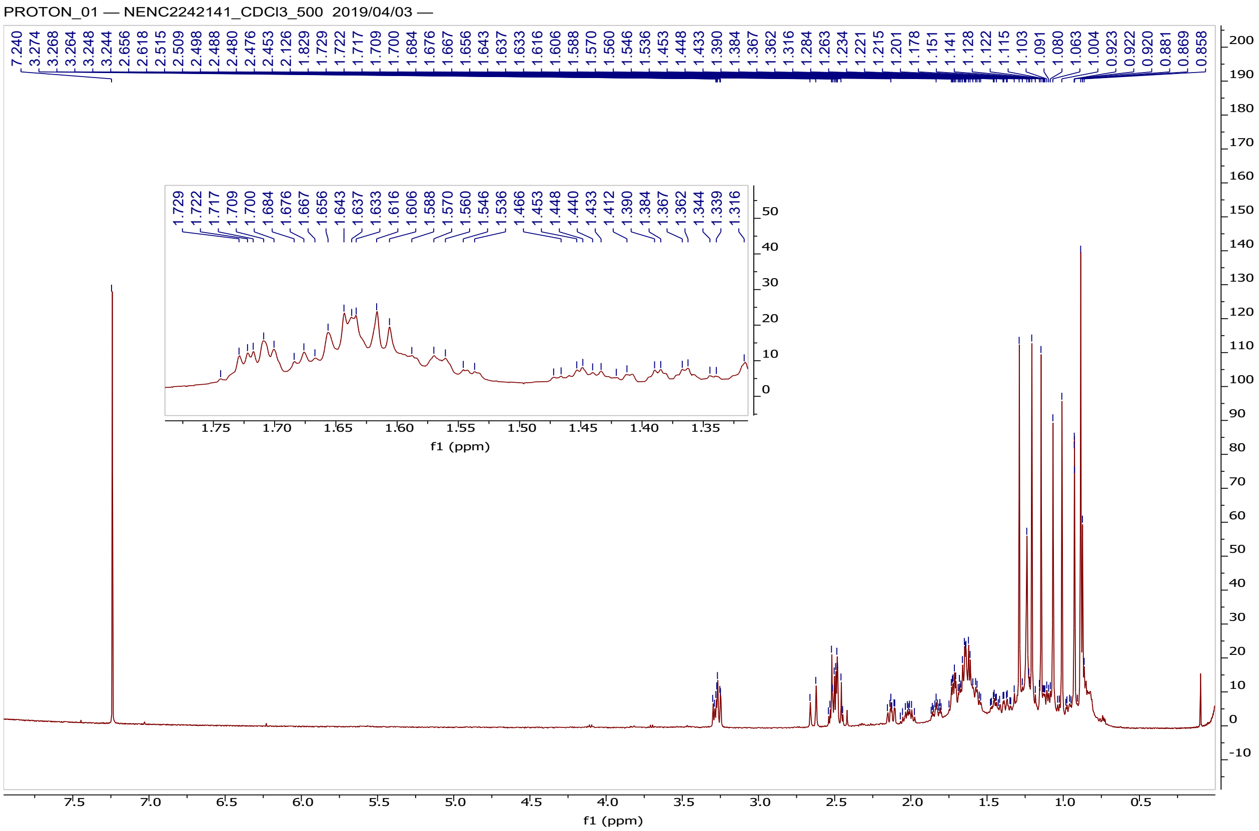

Figure S31. ^{1}H-NMR spectrum of neritriterpenol L (**5**) in $CDCl_3$ (500 MHz)

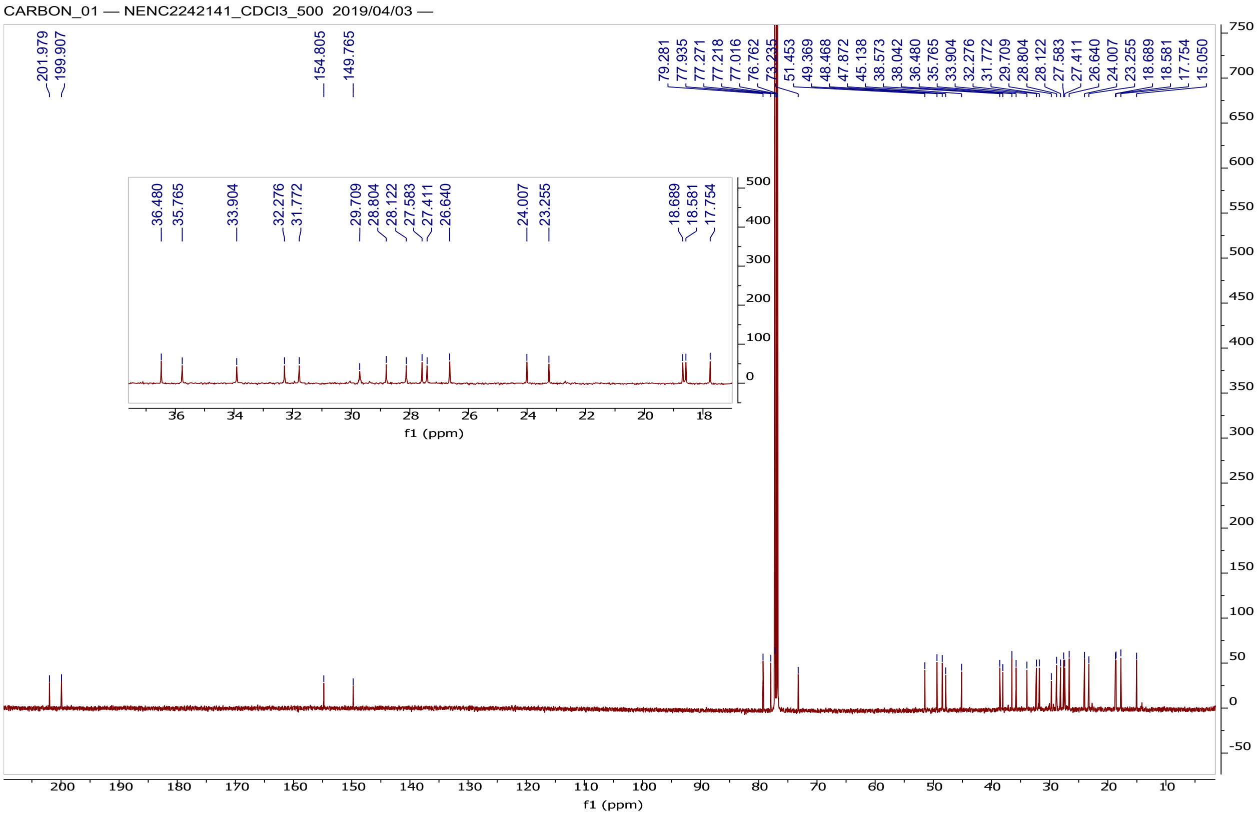

Figure S32. ^{13}C-NMR spectrum of neritriterpenol L (**5**) in $CDCl_3$ (125 MHz)

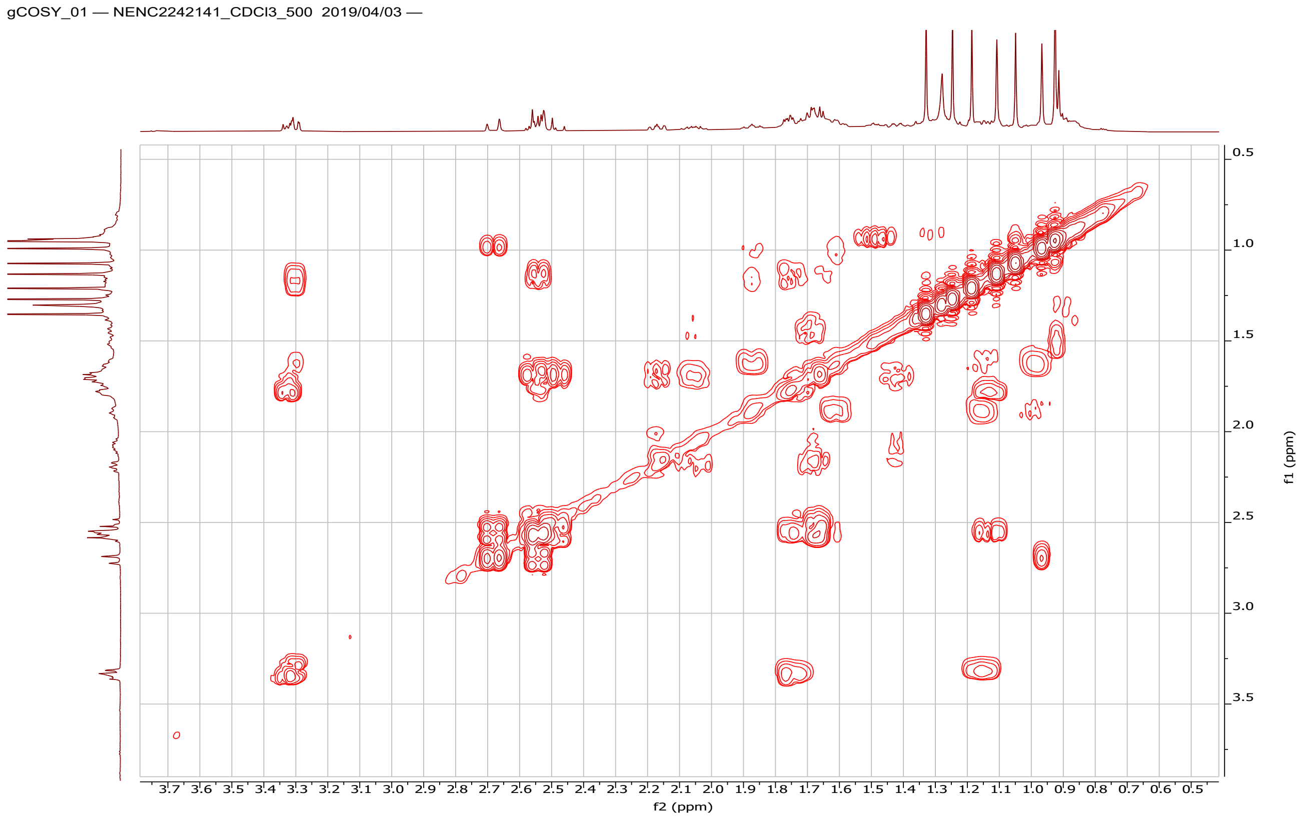

Figure S33. ^{1}H-^{1}H COSY spectrum of neritriterpenol L (**5**) in $CDCl_3$

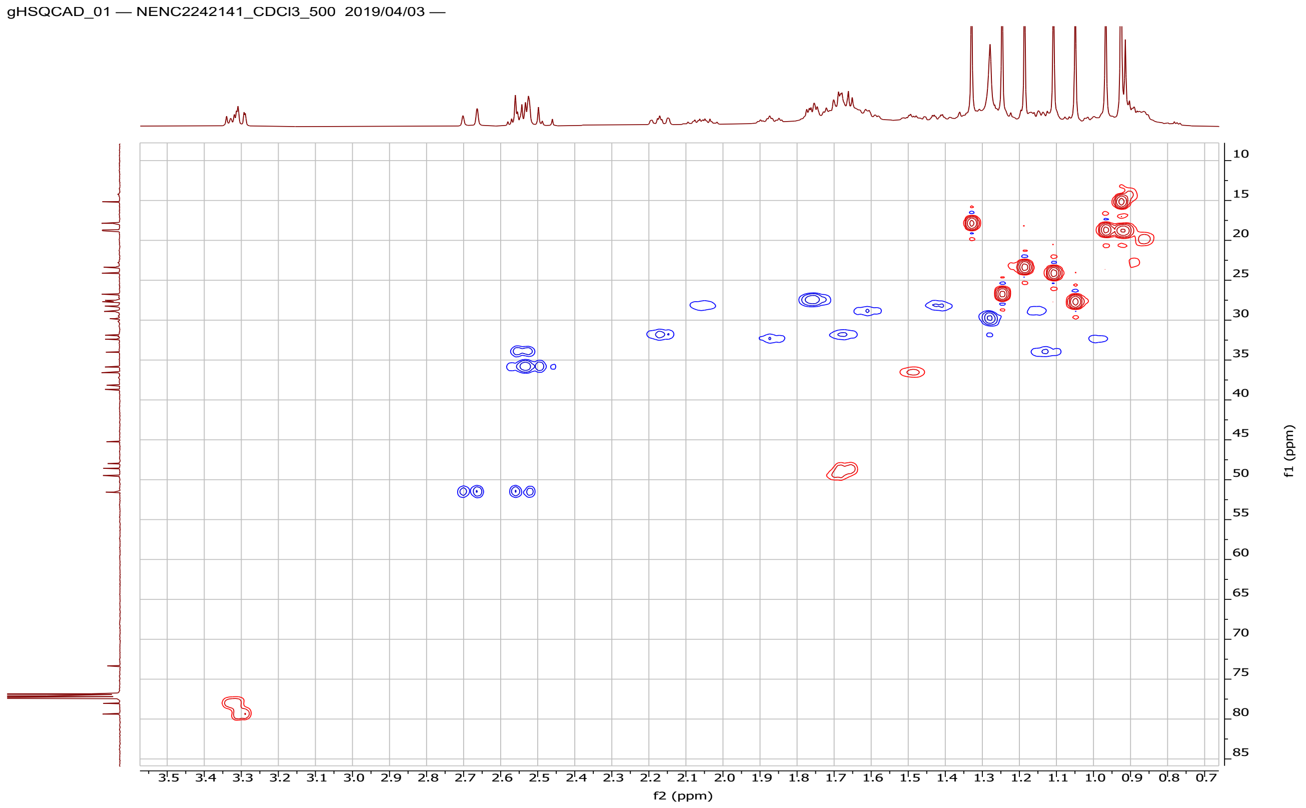

Figure S34. HSQC spectrum of neritriterpenol L (**5**) in $CDCl_3$

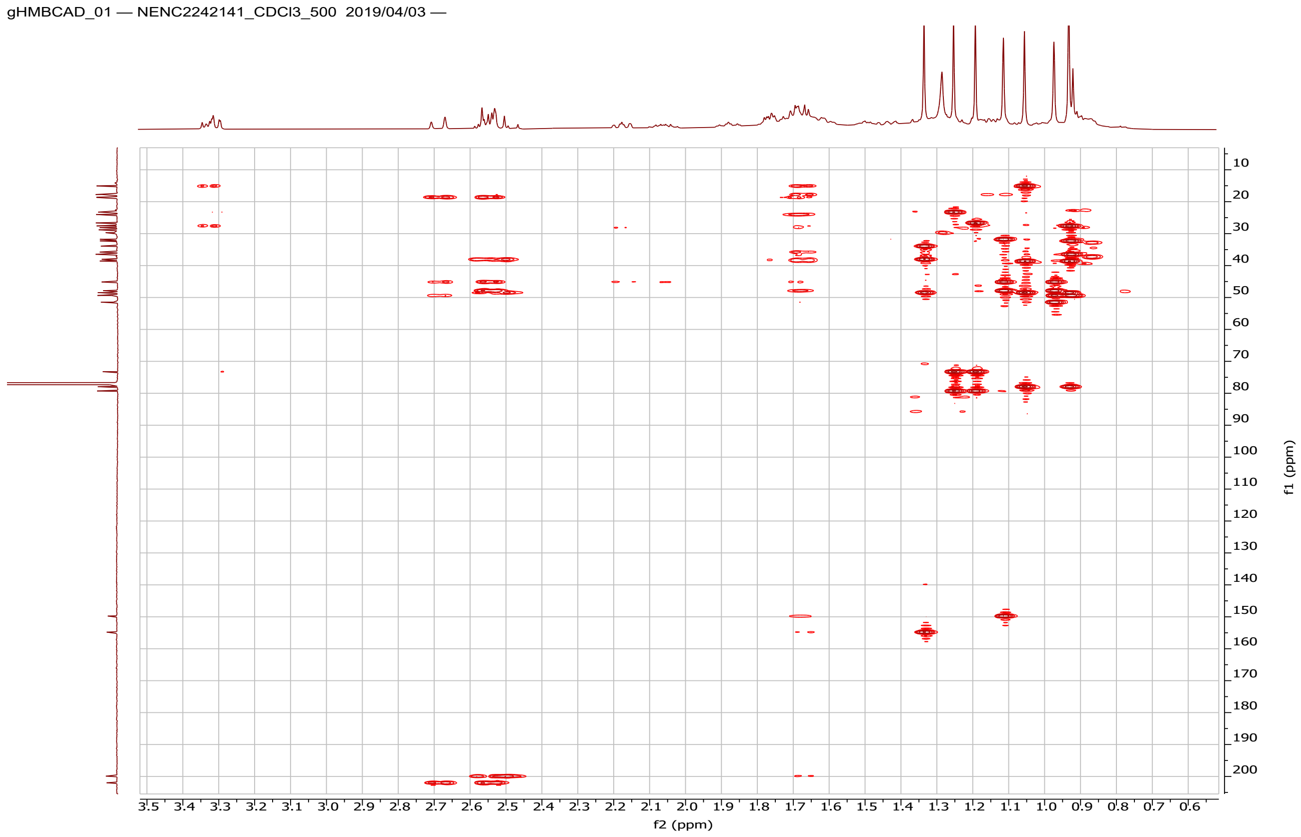

Figure S35. HMBC spectrum of neritriterpenol L (**5**) in $CDCl_3$

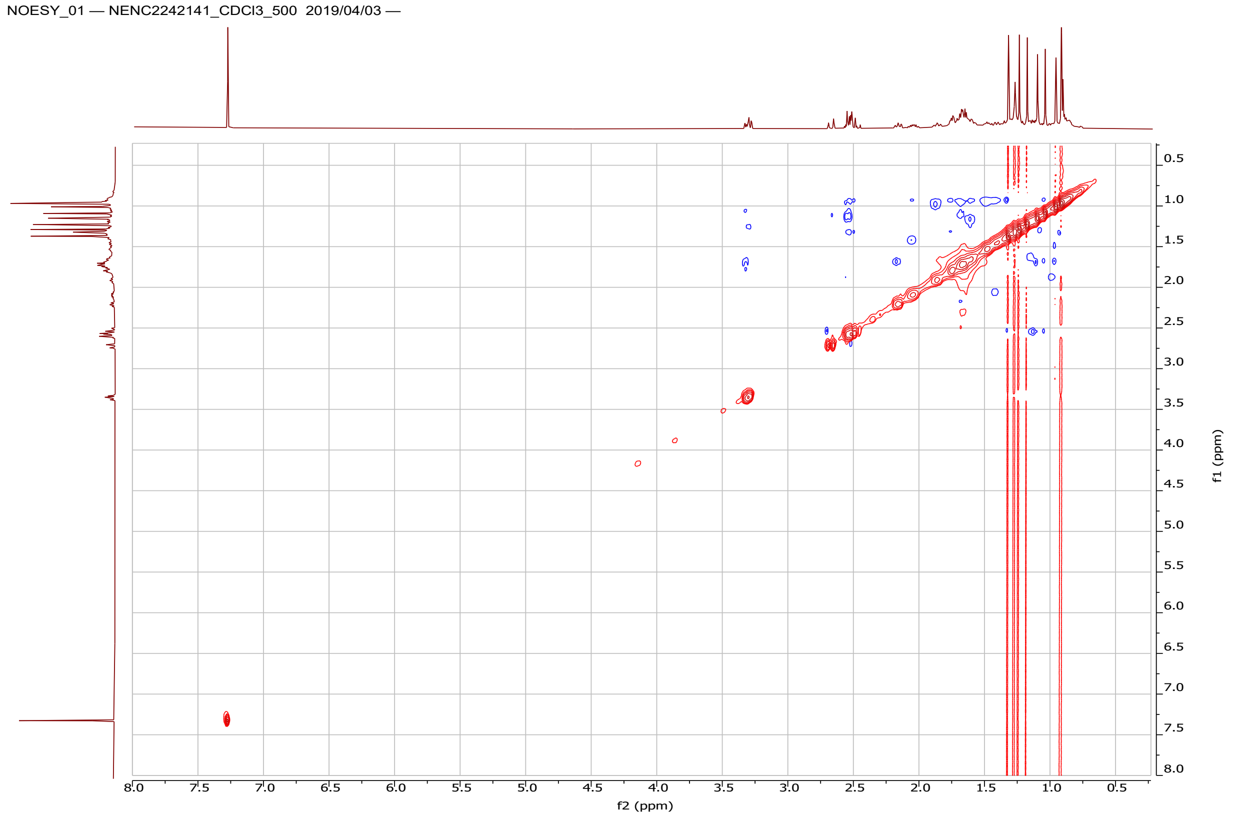

Figure S36. NOESY spectrum of neritriterpenol L (**5**) in $CDCl_3$

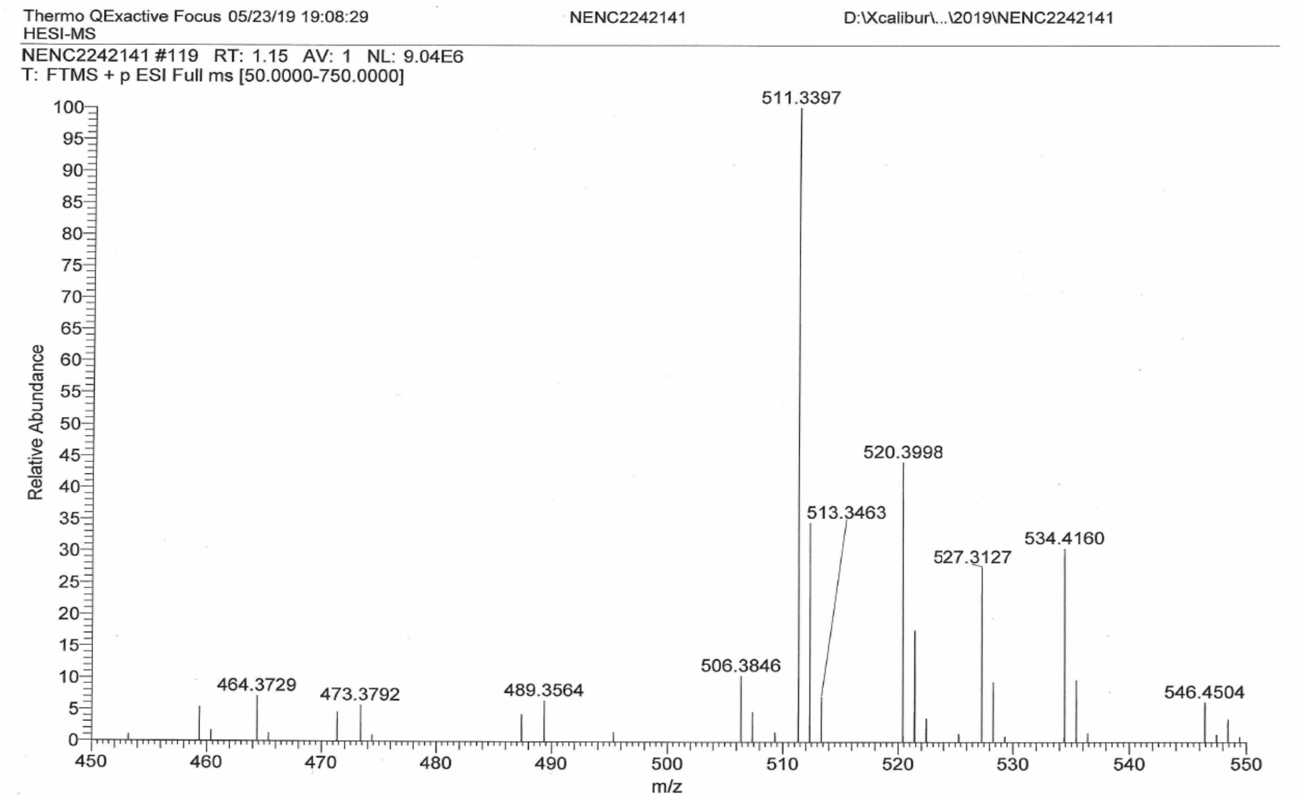

Figure S37. HRESIMS spectrum of neritriterpenol L (**5**) in $CDCl_3$

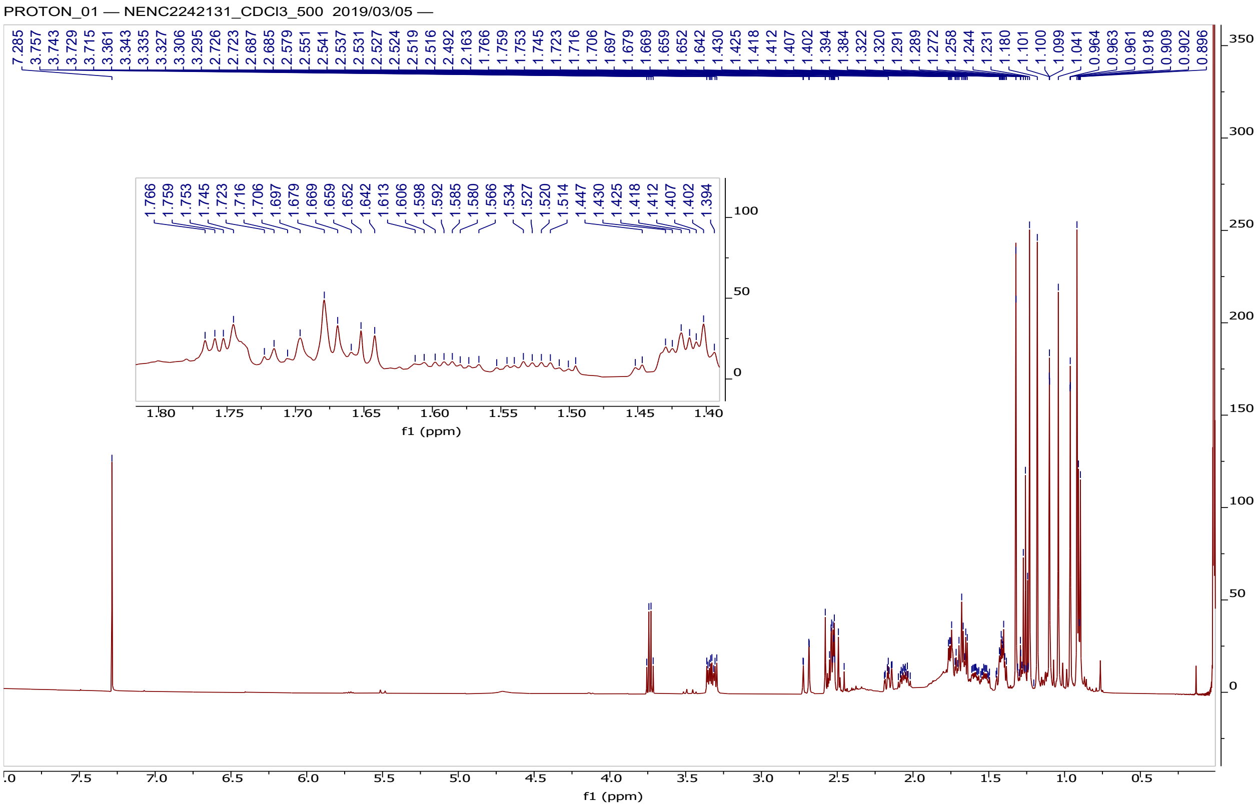

Figure S38. ^{1}H-NMR spectrum of neritriterpenol M (**6**) in $CDCl_3$ (500 MHz)

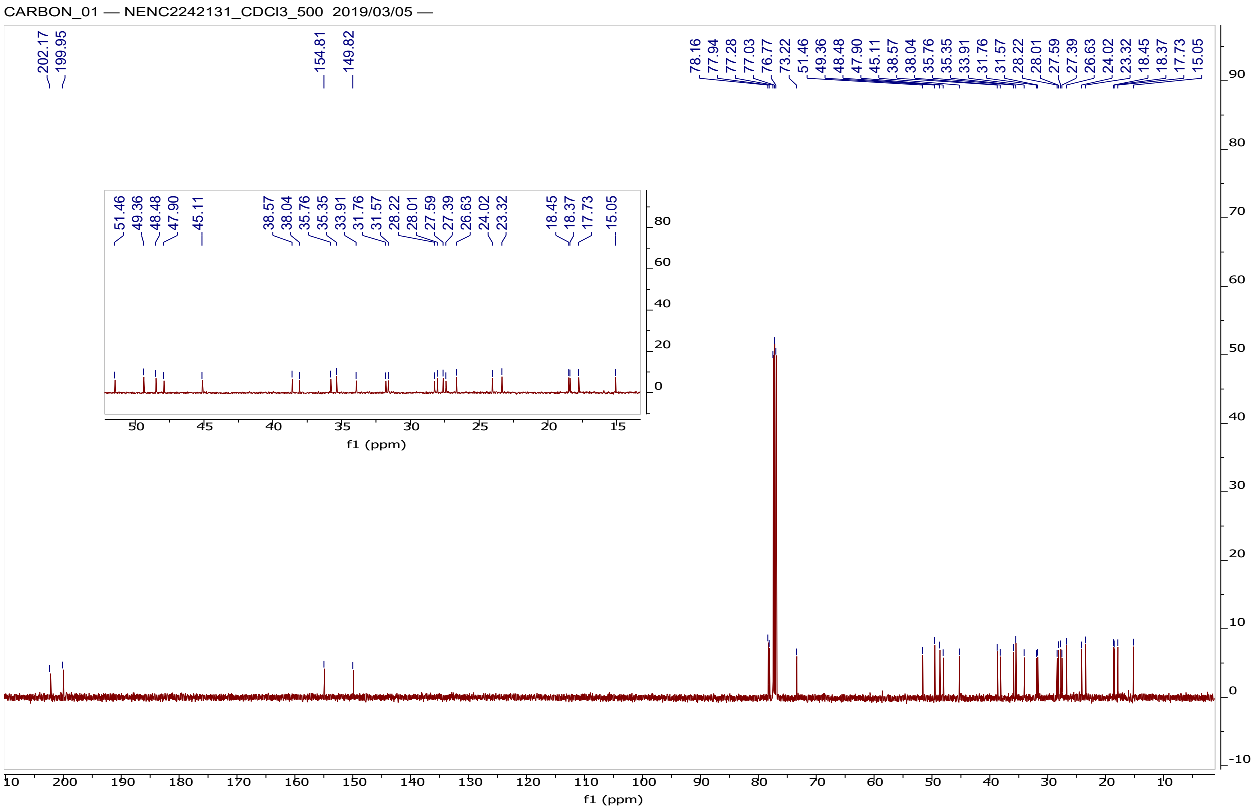

Figure S39. ^{13}C-NMR spectrum of neritriterpenol M (**6**) in $CDCl_3$ (125 MHz)

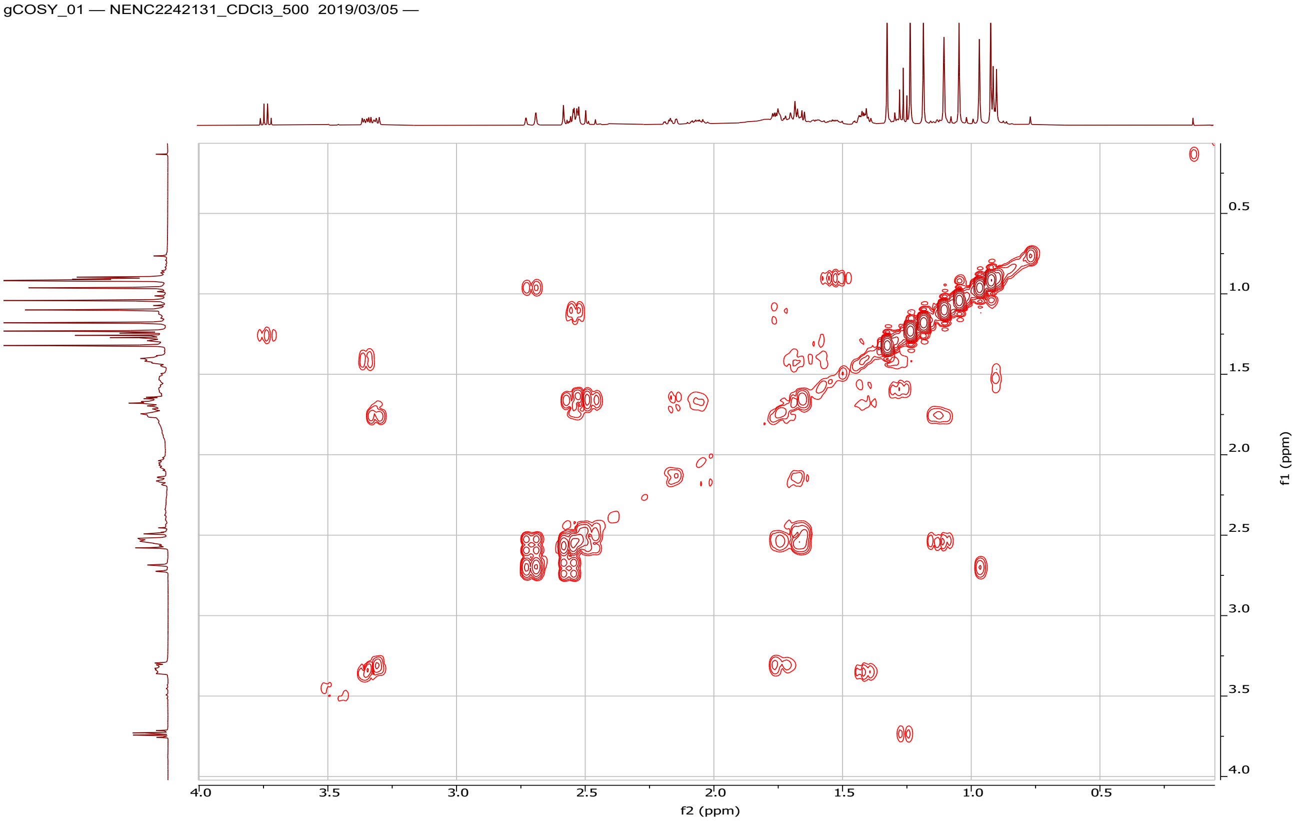

Figure S40. ^{1}H-^{1}H COSY spectrum of neritriterpenol M (**6**) in $CDCl_3$

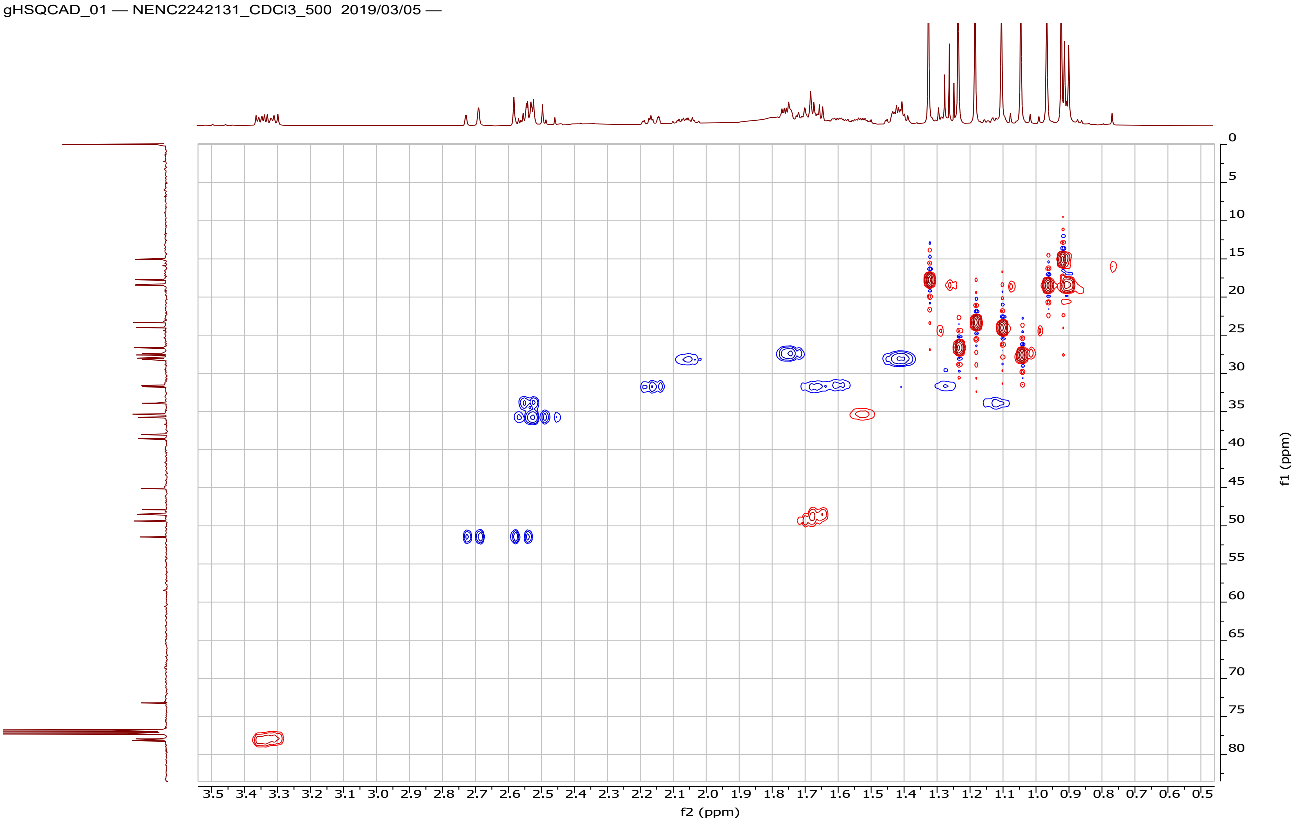

Figure S41. HSQC spectrum of neritriterpenol M (**6**) in $CDCl_3$

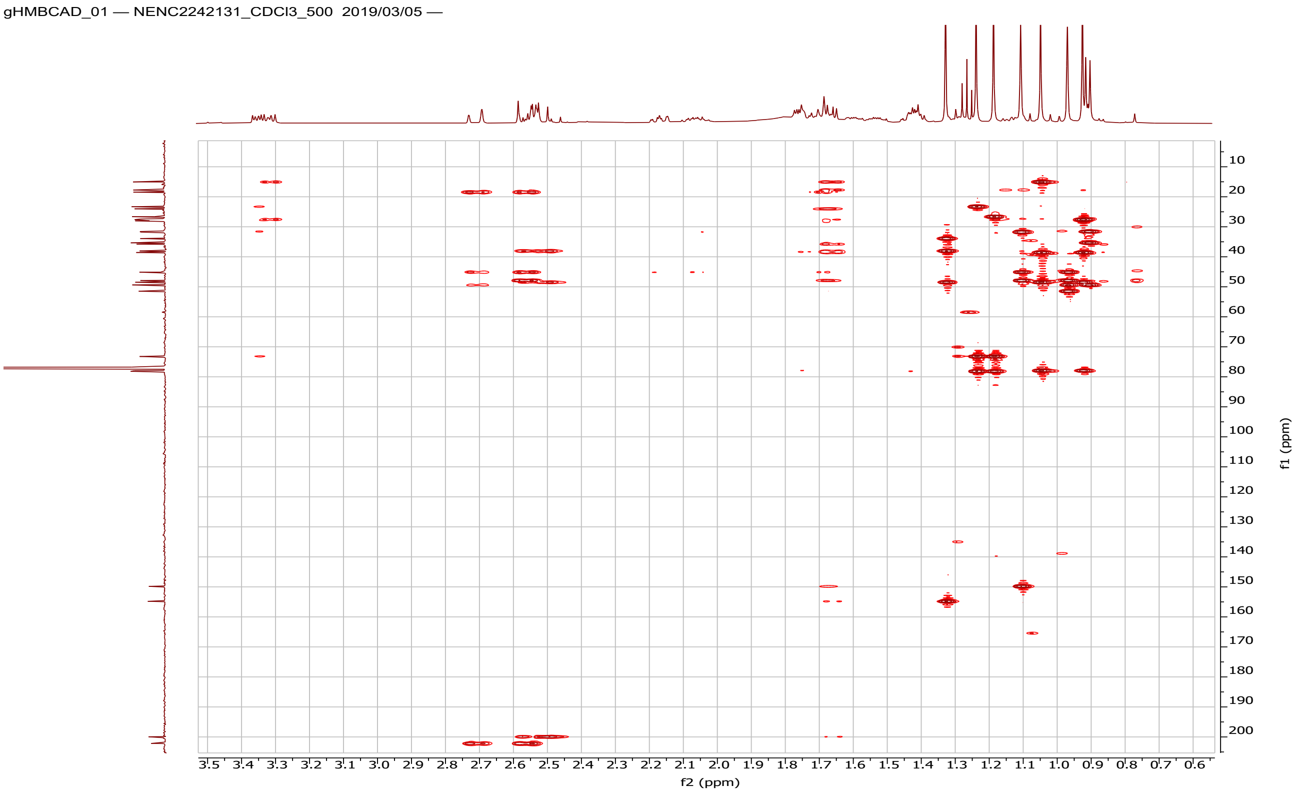

Figure S42. HMBC spectrum of neritriterpenol M (**6**) in $CDCl_3$

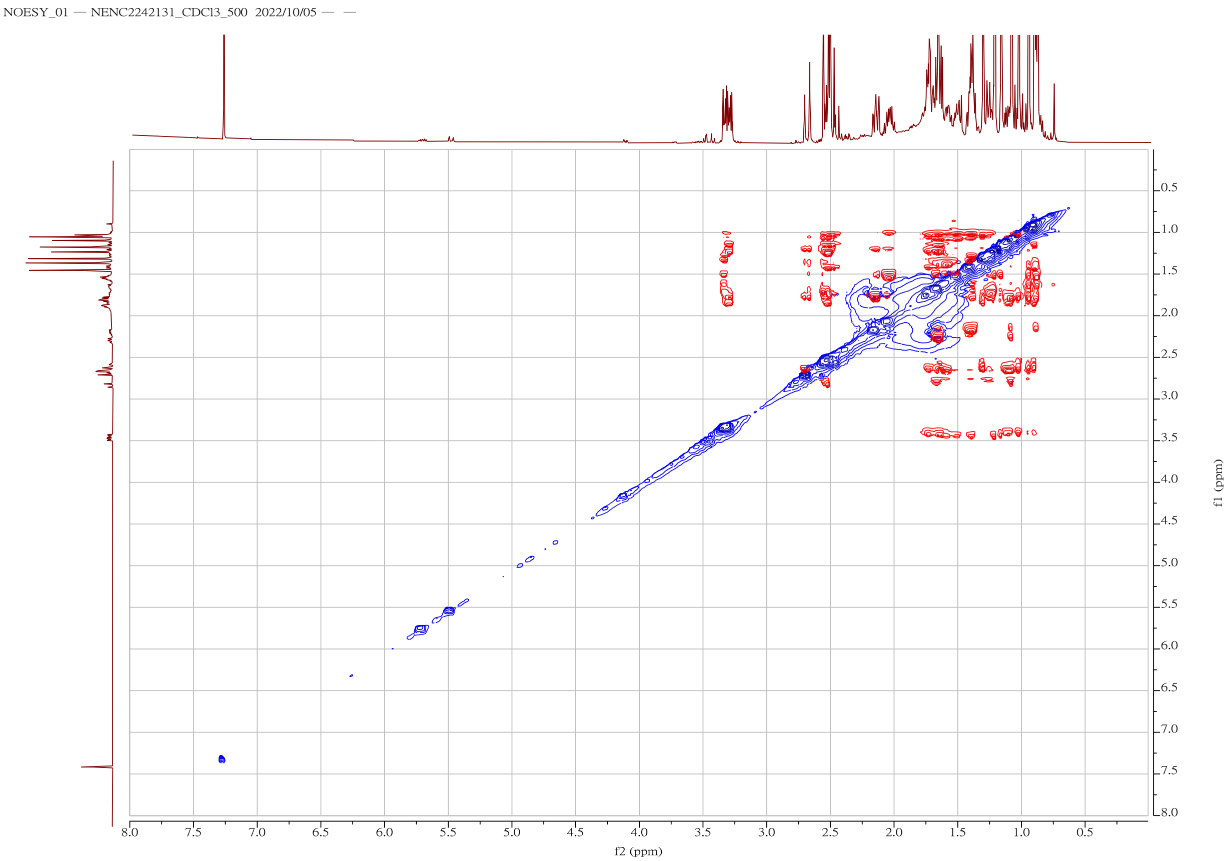

Figure S43. NOESY spectrum of neritriterpenol M (**6**) in $CDCl_3$

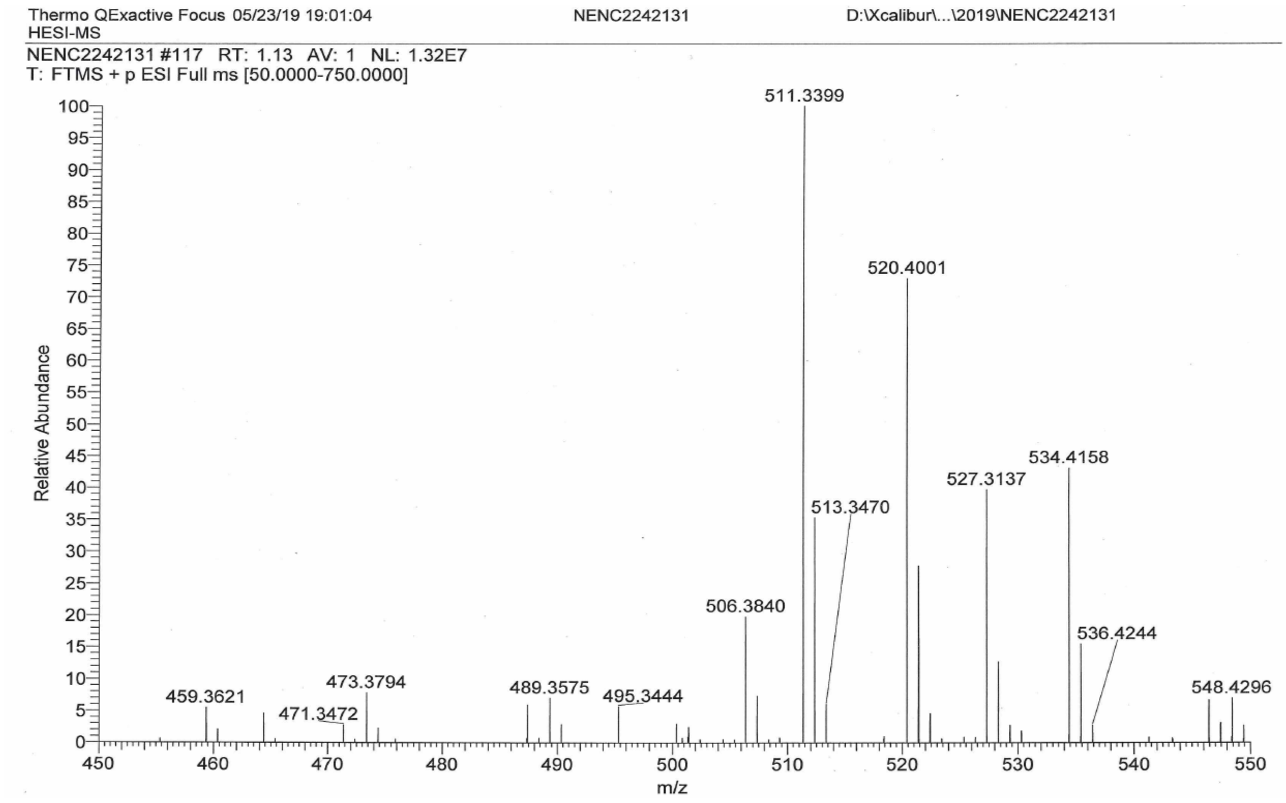

Figure S44. HRESIMS spectrum of neritriterpenol M (**6**)

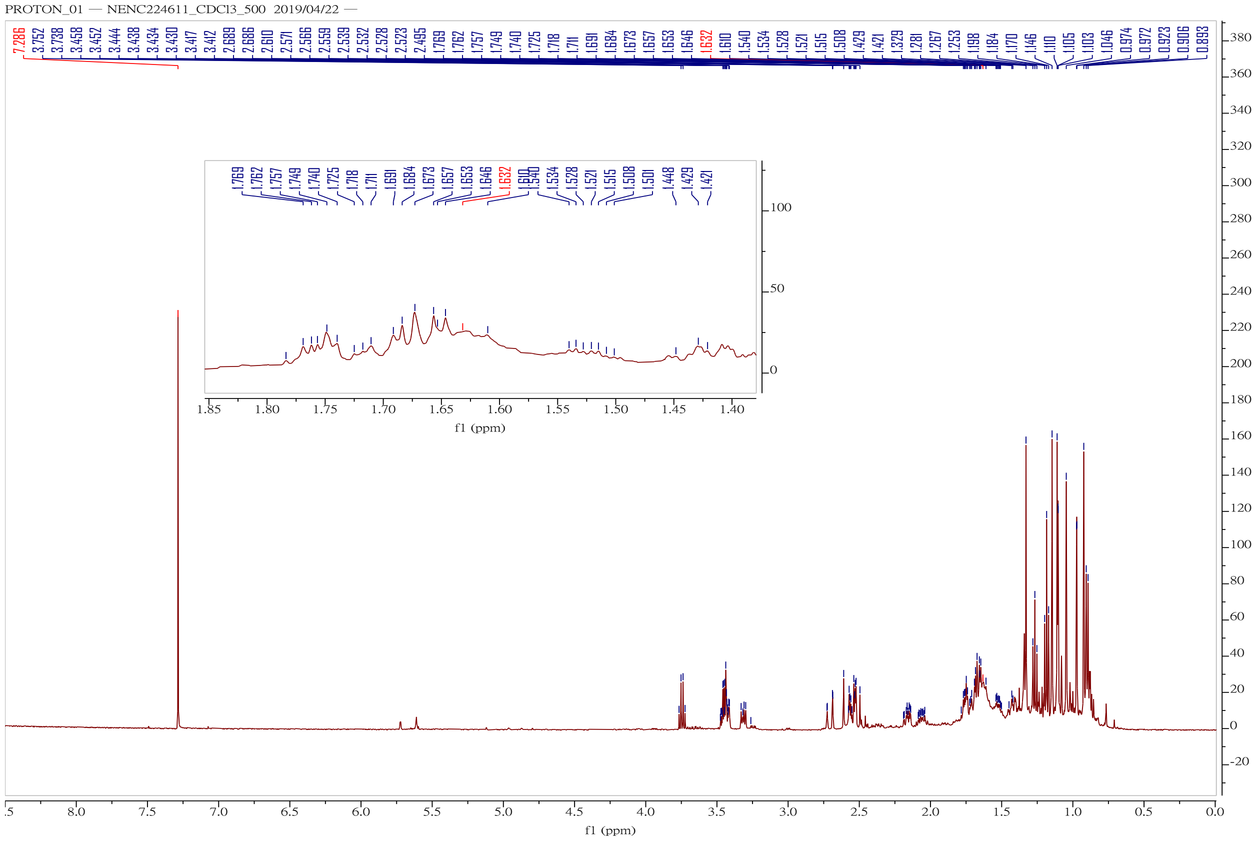

Figure S45. ^{1}H-NMR spectrum of neritriterpenol N (**7**) in $CDCl_3$ (500 MHz)

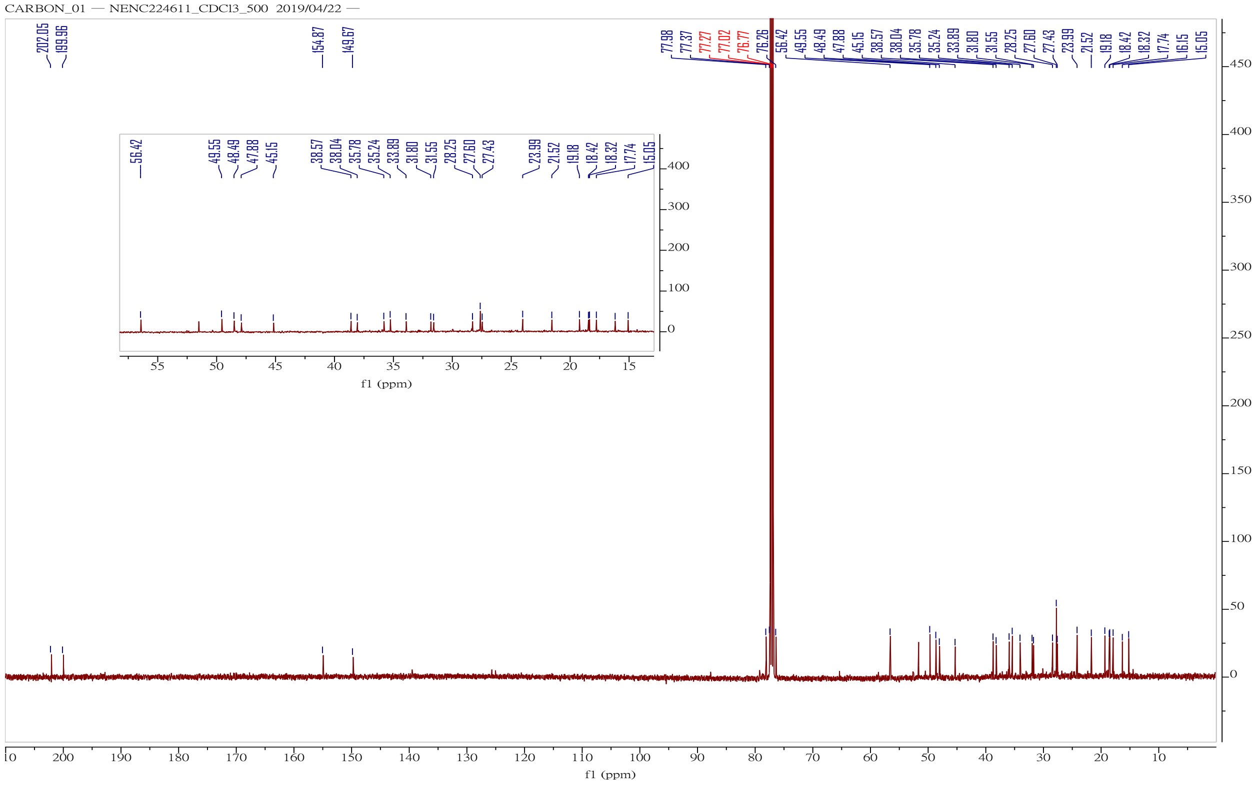

Figure S46. ^{13}C-NMR spectrum of neritriterpenol N (**7**) in $CDCl_3$ (125 MHz)

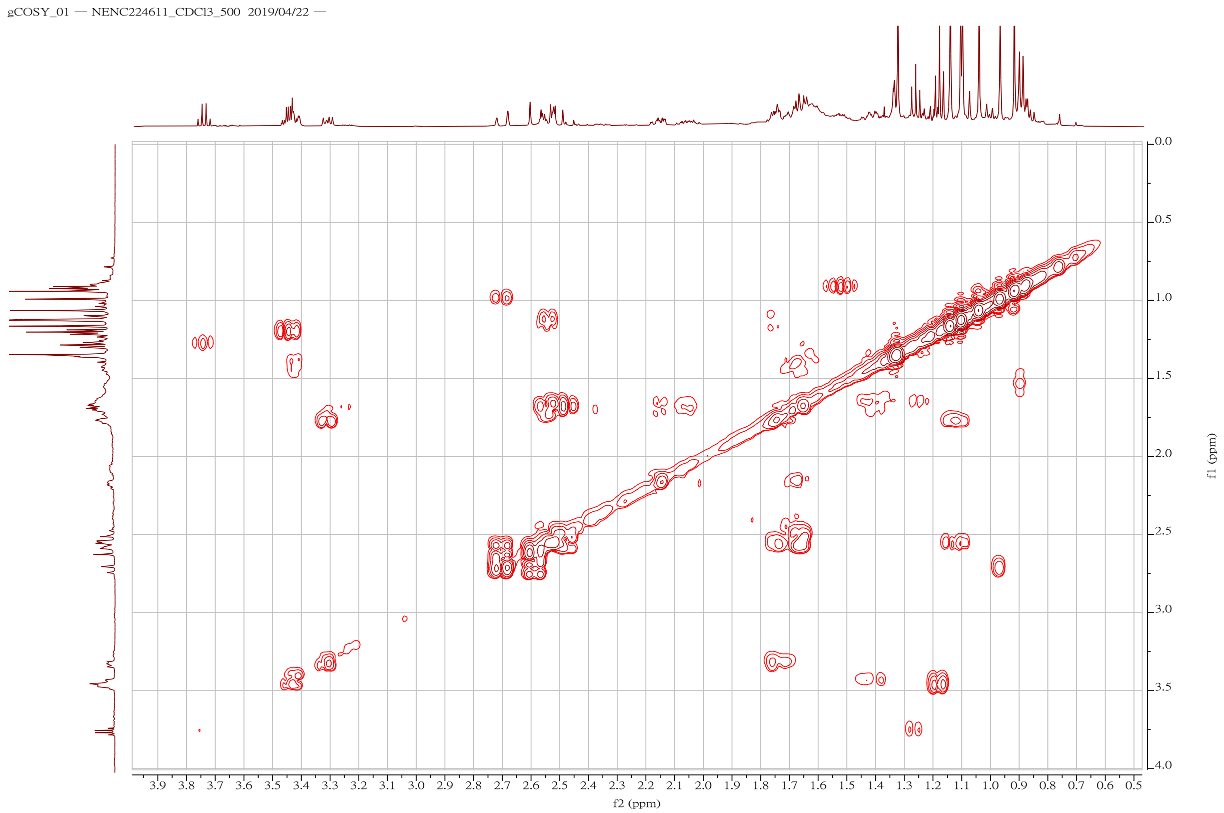

Figure S47. ^{1}H-^{1}H COSY spectrum of neritriterpenol N (**7**) in $CDCl_3$

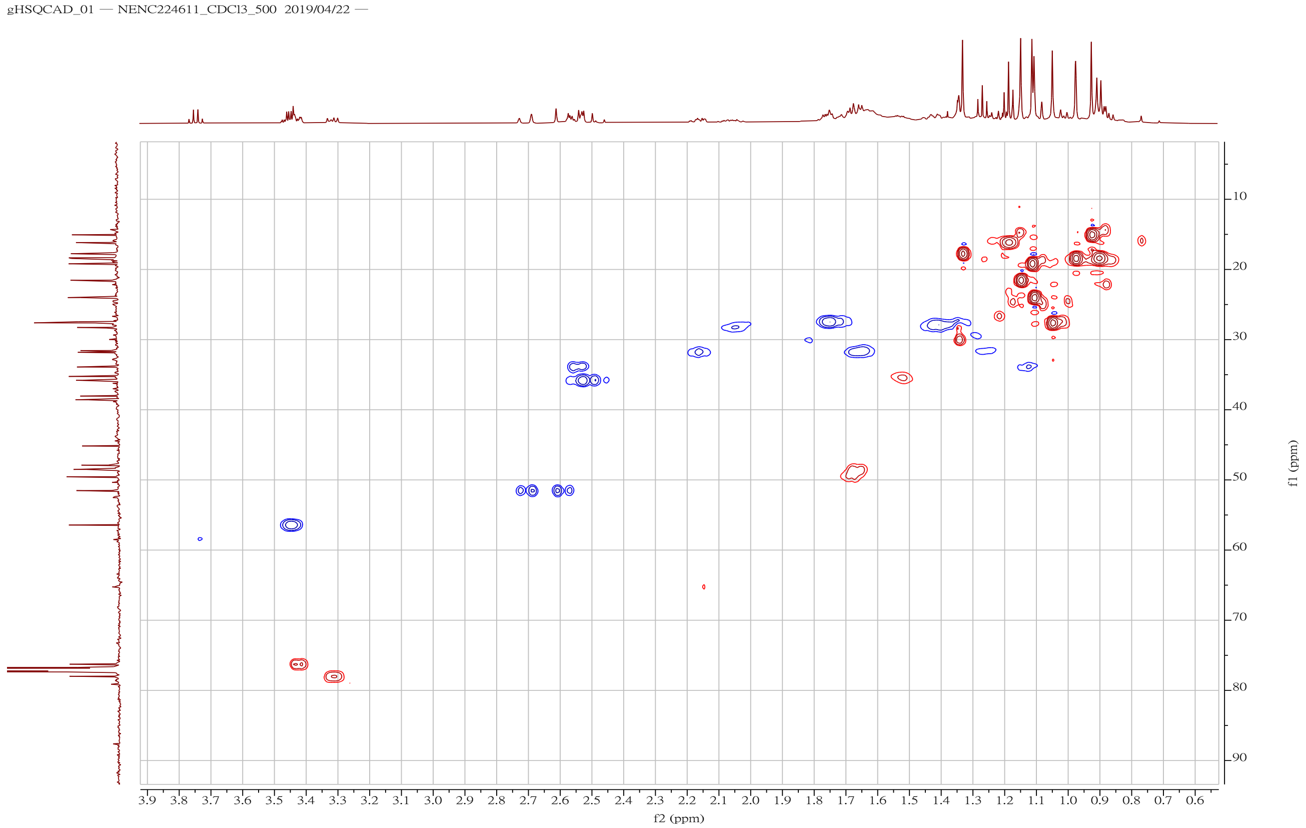

Figure S48. HSQC spectrum of neritriterpenol N (**7**) in $CDCl_3$

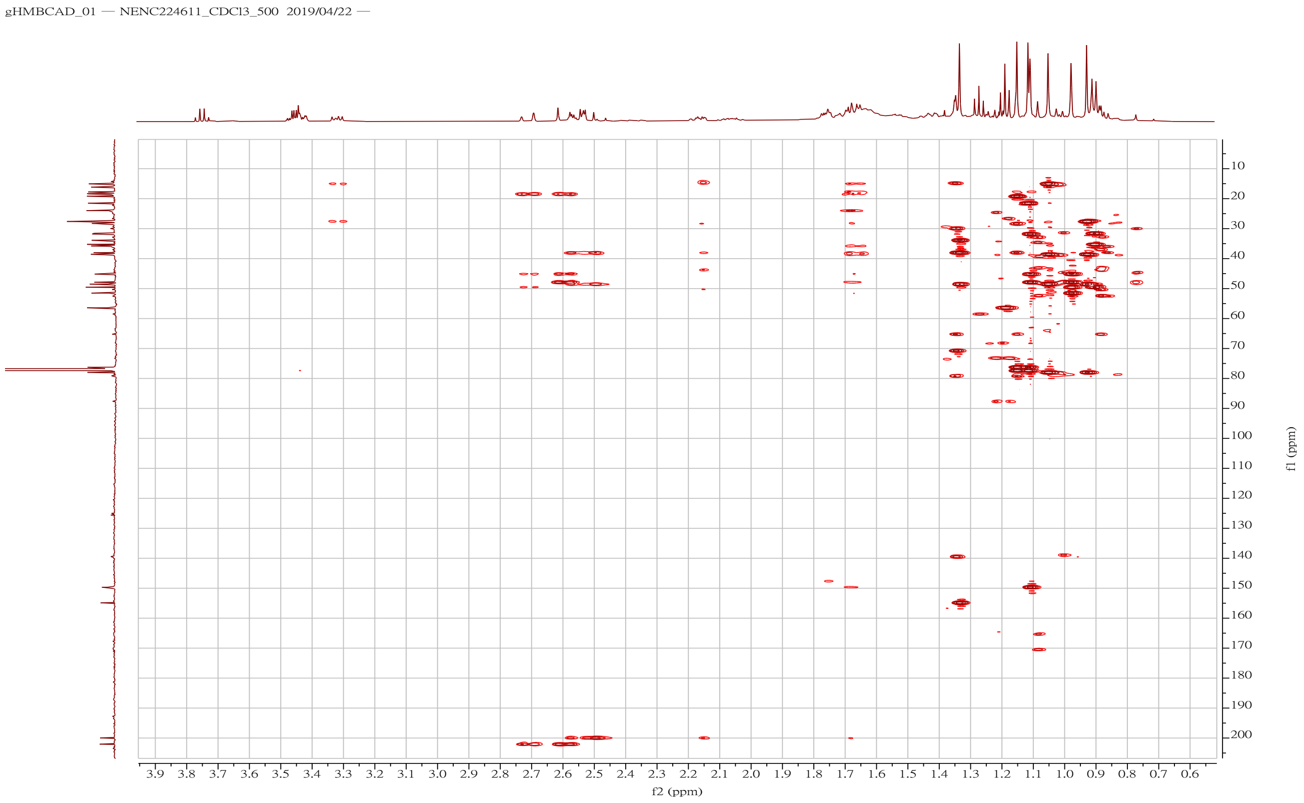

Figure S49. HMBC spectrum of neritriterpenol N (**7**) in $CDCl_3$

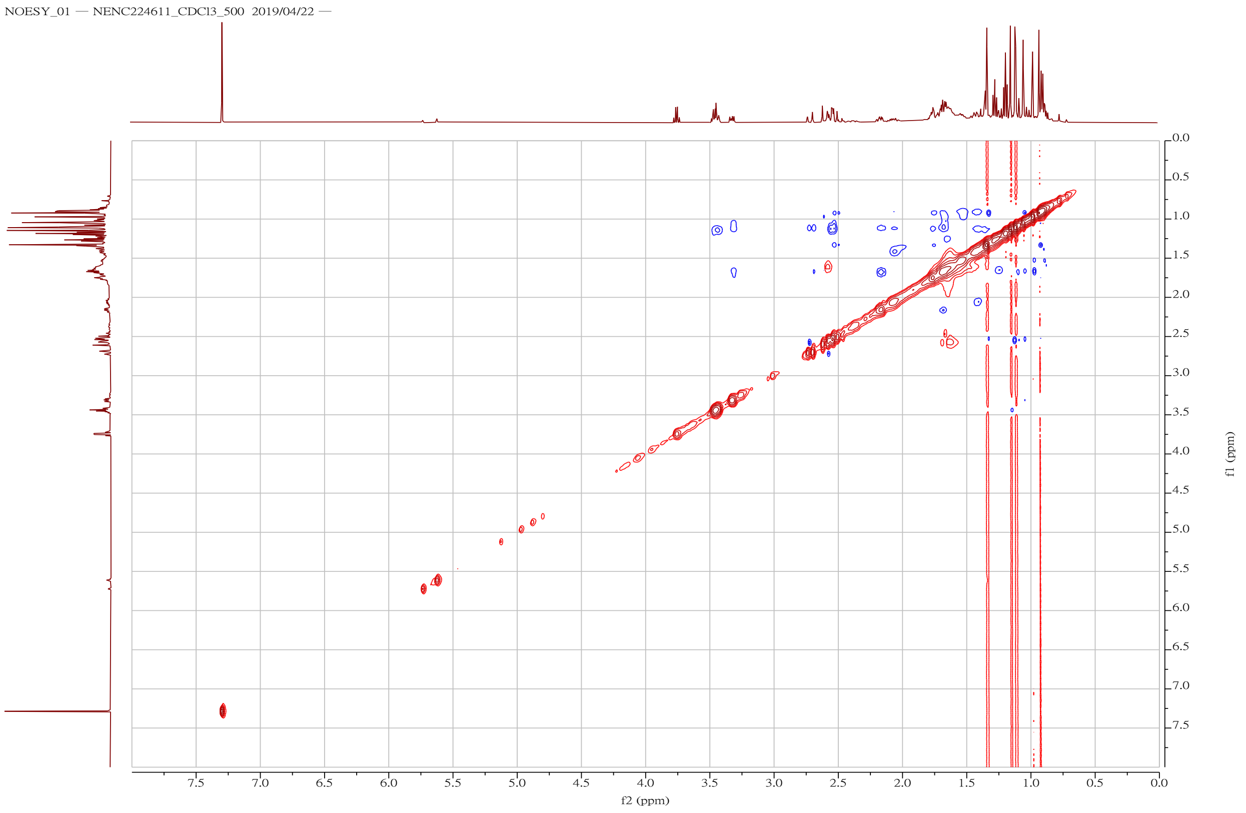

Figure S50. NOESY spectrum of neritriterpenol N (**7**) in $CDCl_3$

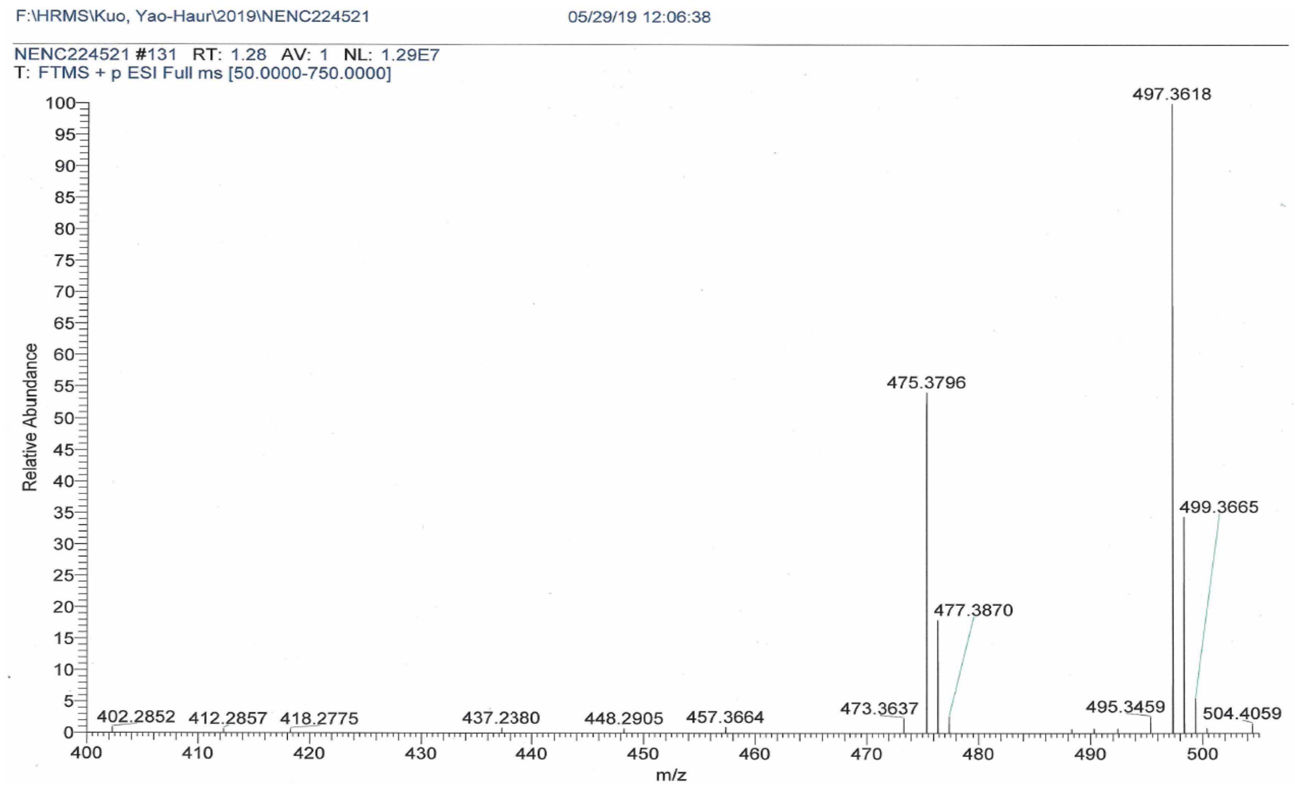

Figure S51. HRESIMS spectrum of neritriterpenol N (**7**)

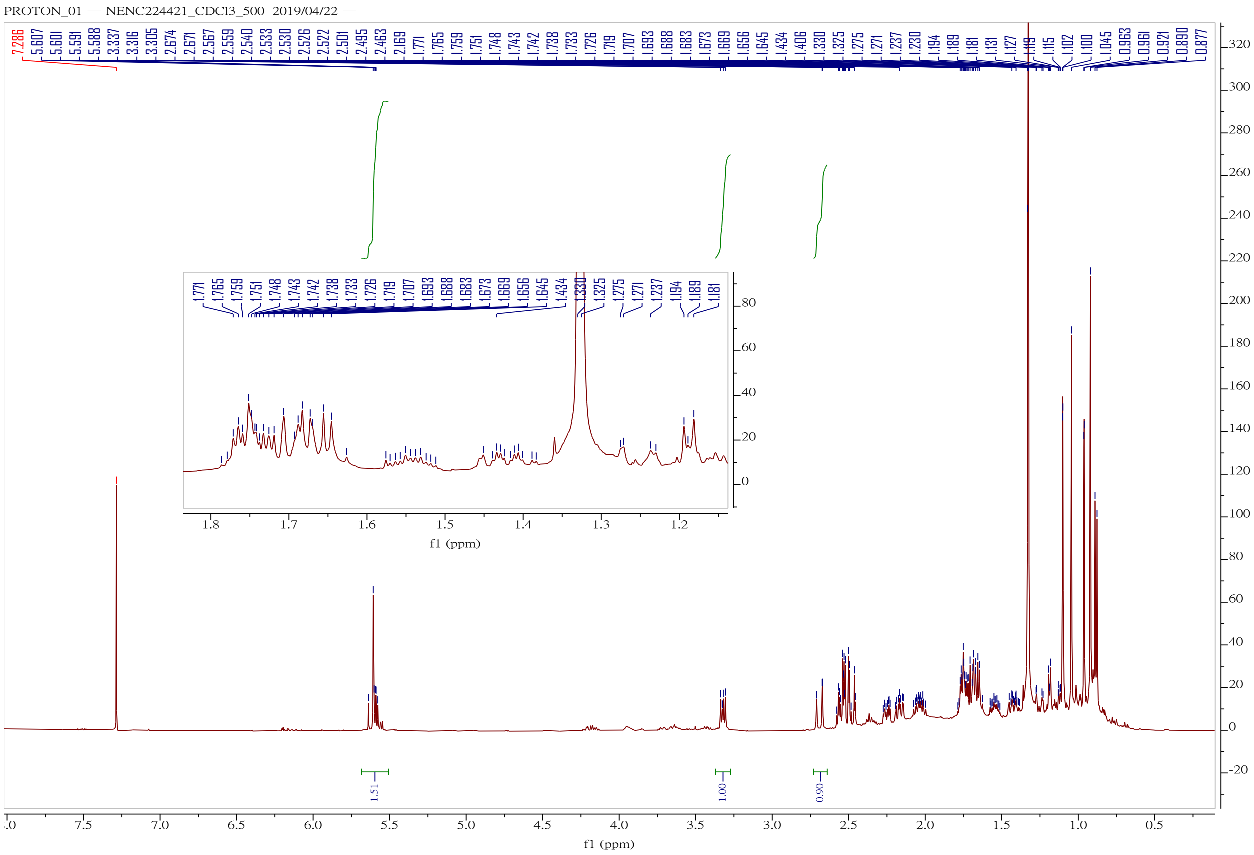

Figure S52. ^{1}H-NMR spectrum of 11-oxo-kansenonol (**8**) in $CDCl_3$ (500 MHz)

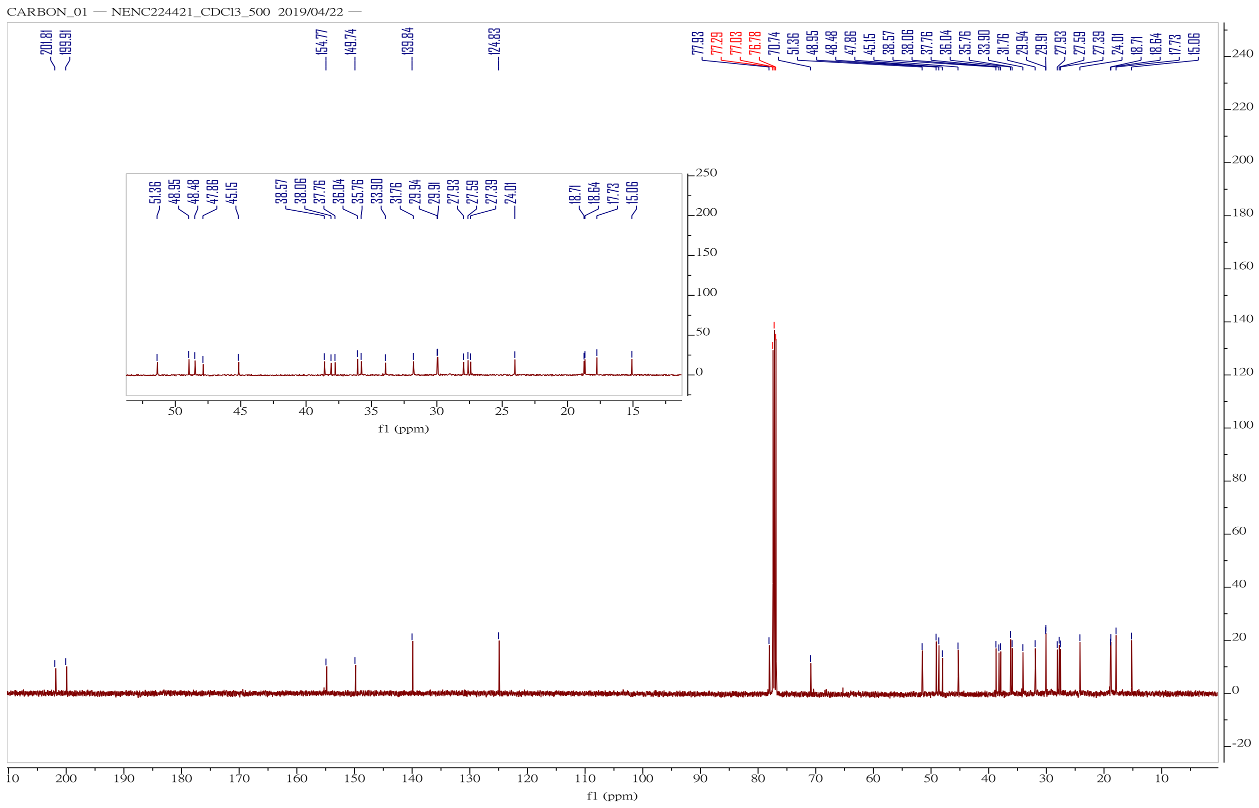

Figure S53. ^{13}C-NMR spectrum of 11-oxo-kansenonol (**8**) in $CDCl_3$ (125 MHz)

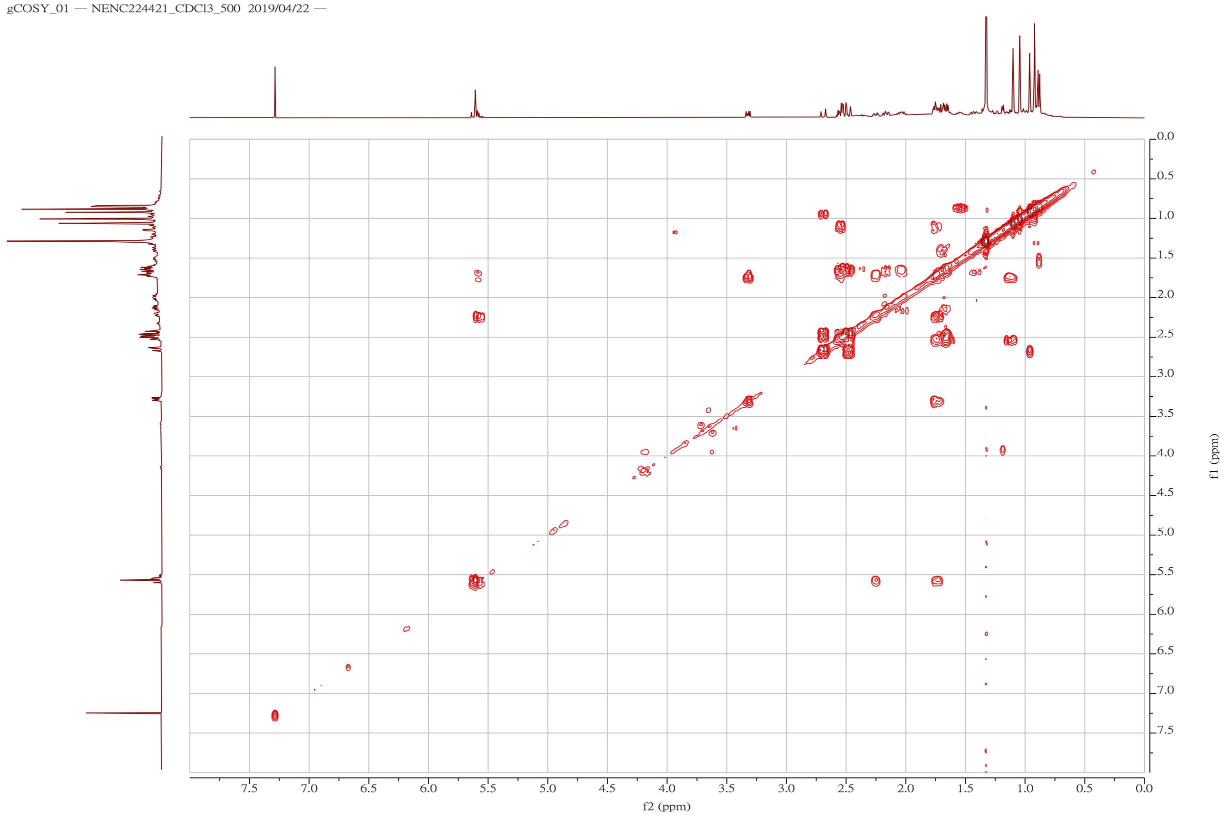

Figure S54. ^{1}H-^{1}H COSY spectrum of 11-oxo-kansenonol (**8**) in $CDCl_3$

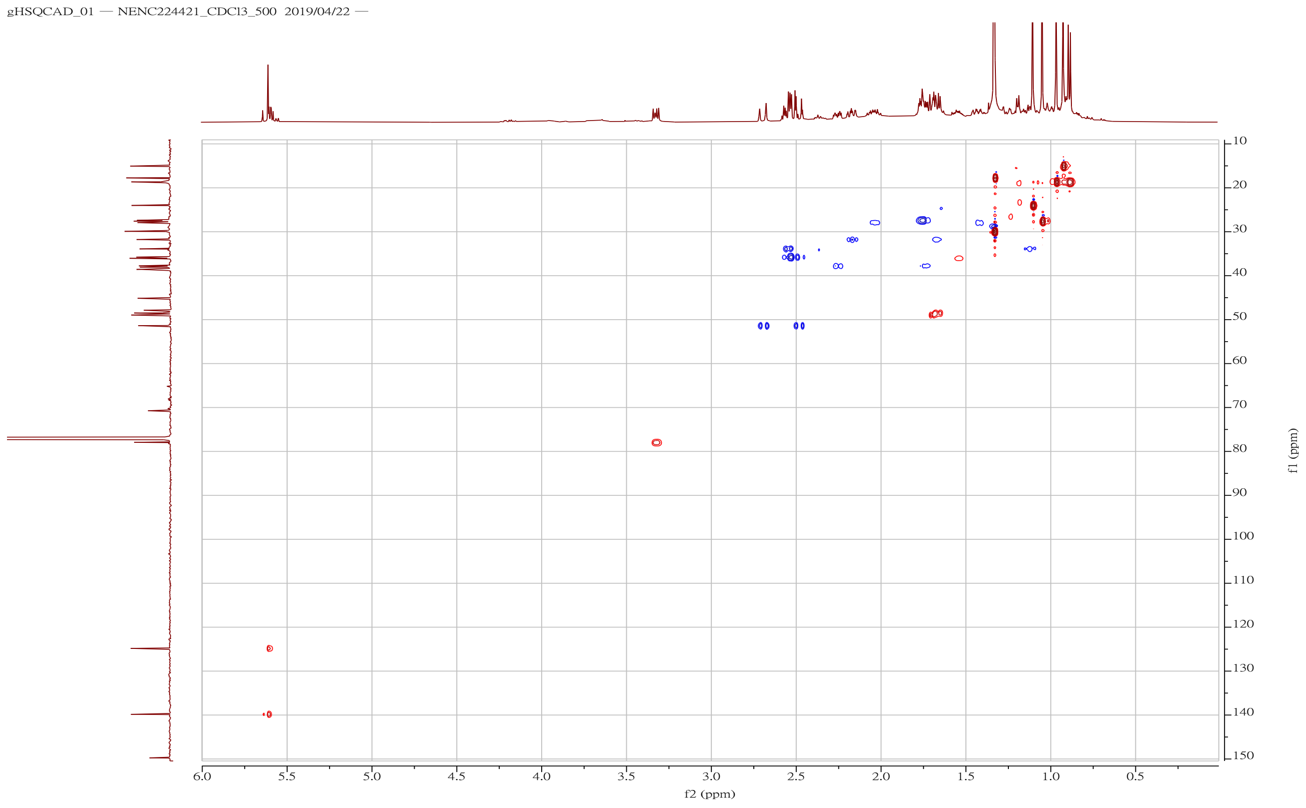

Figure S55. HSQC spectrum of 11-oxo-kansenonol (**8**) in $CDCl_3$

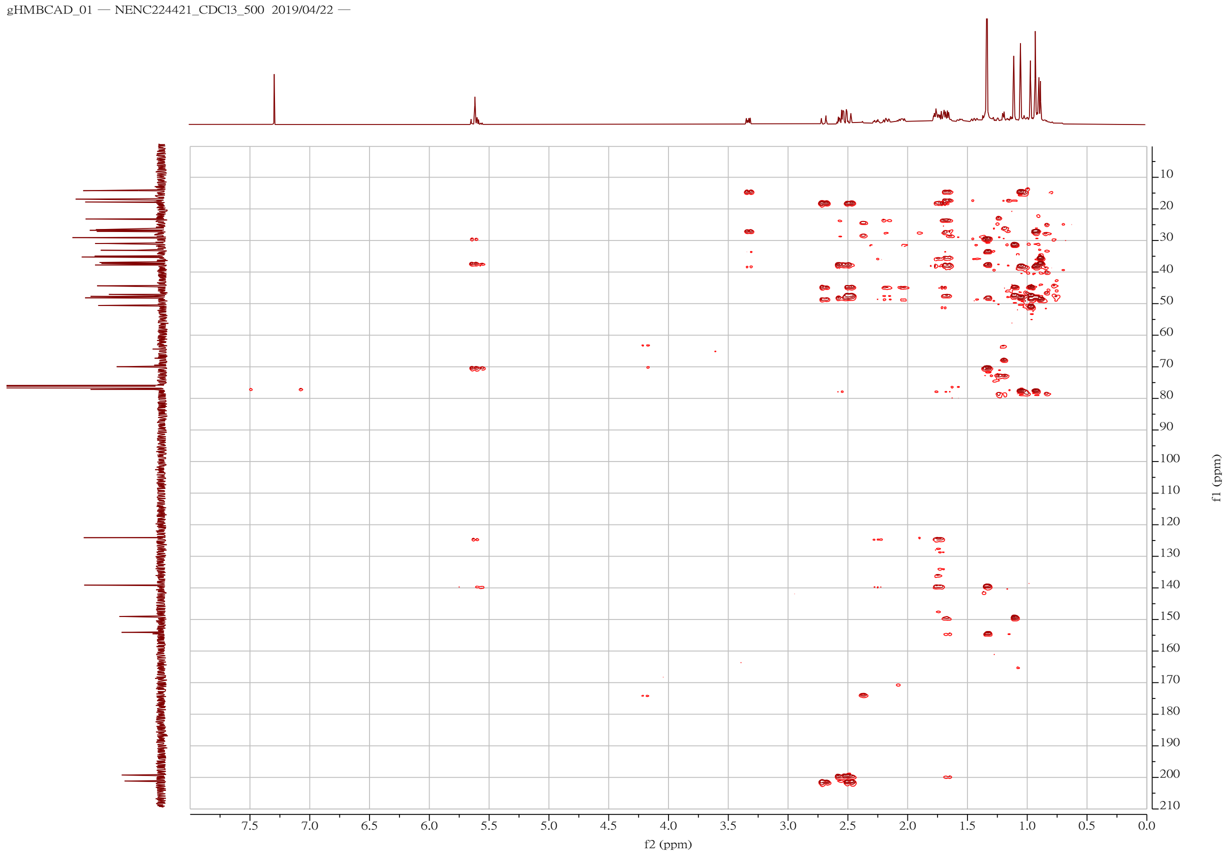

Figure S56. HMBC spectrum of 11-oxo-kansenonol (**8**) in $CDCl_3$

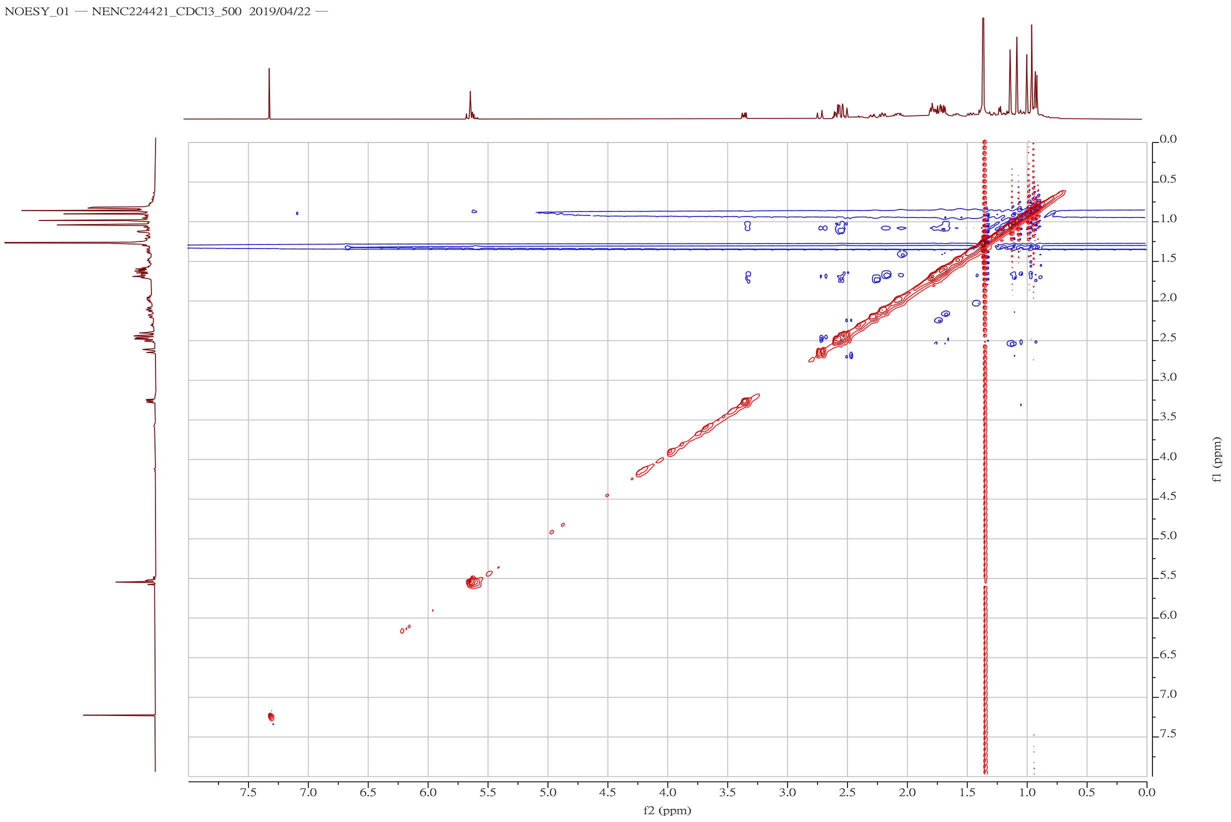

Figure S57. NOESY spectrum of 11-oxo-kansenonol (**8**) in $CDCl_3$

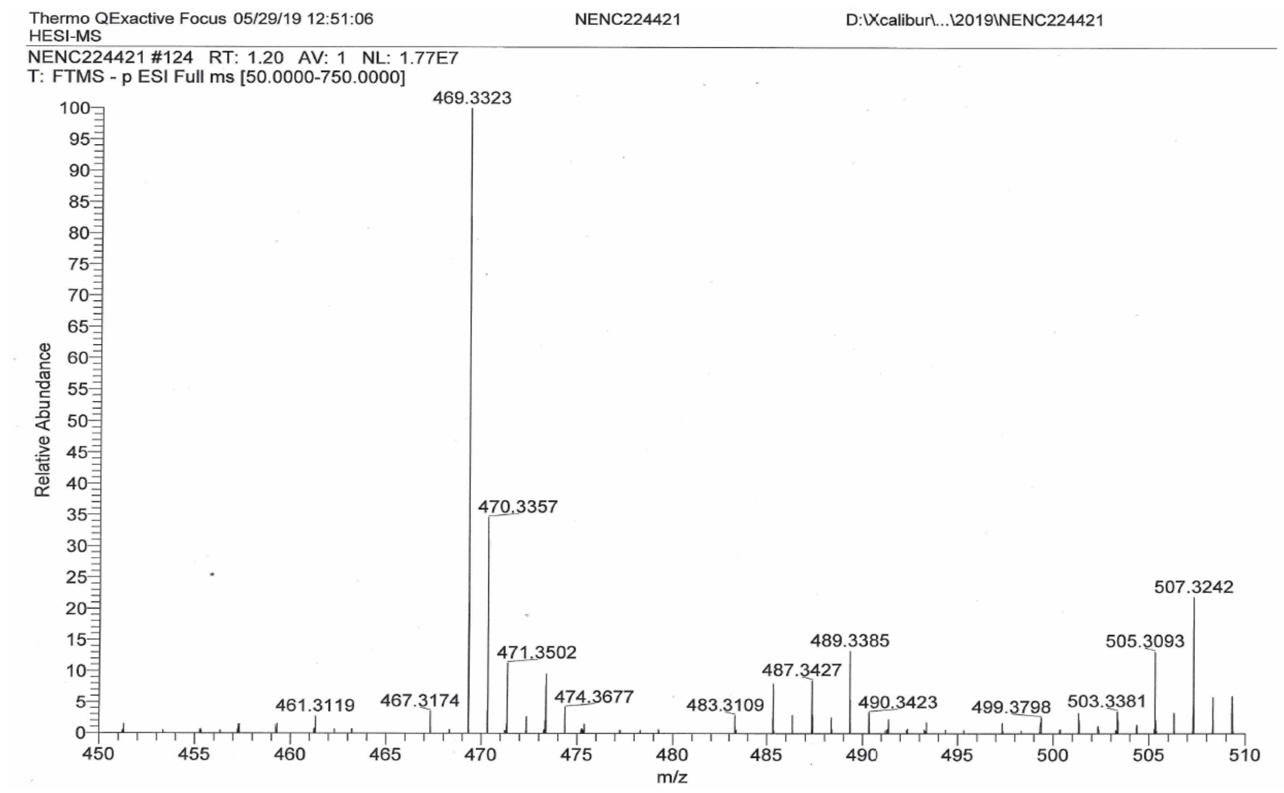

Figure S58. HRESIMS spectrum of 11-oxo-kansenonol (**8**)
